# Haplogenome assembly reveals structural variation in *Eucalyptus* interspecific hybrids

**DOI:** 10.1093/gigascience/giad064

**Published:** 2023-08-26

**Authors:** Anneri Lötter, Tuan A Duong, Julia Candotti, Eshchar Mizrachi, Jill L Wegrzyn, Alexander A Myburg

**Affiliations:** Department of Biochemistry, Genetics and Microbiology, Forestry and Agricultural Biotechnology Institute (FABI), University of Pretoria, Private bag X20, Pretoria 0028, South Africa; Department of Biochemistry, Genetics and Microbiology, Forestry and Agricultural Biotechnology Institute (FABI), University of Pretoria, Private bag X20, Pretoria 0028, South Africa; Department of Biochemistry, Genetics and Microbiology, Forestry and Agricultural Biotechnology Institute (FABI), University of Pretoria, Private bag X20, Pretoria 0028, South Africa; Department of Biochemistry, Genetics and Microbiology, Forestry and Agricultural Biotechnology Institute (FABI), University of Pretoria, Private bag X20, Pretoria 0028, South Africa; Department of Ecology and Evolutionary Biology, Institute for Systems Genomics: Computational Biology Core, University of Connecticut, Storrs, CT 06269, USA; Department of Biochemistry, Genetics and Microbiology, Forestry and Agricultural Biotechnology Institute (FABI), University of Pretoria, Private bag X20, Pretoria 0028, South Africa

**Keywords:** *Eucalyptus*, trio-binning, phased genome assembly, Nanopore, structural variant

## Abstract

**Background:**

*De novo* phased (haplo)genome assembly using long-read DNA sequencing data has improved the detection and characterization of structural variants (SVs) in plant and animal genomes. Able to span across haplotypes, long reads allow phased, haplogenome assembly in highly outbred organisms such as forest trees. *Eucalyptus* tree species and interspecific hybrids are the most widely planted hardwood trees with F1 hybrids of *Eucalyptus grandis* and *E. urophylla* forming the bulk of fast-growing pulpwood plantations in subtropical regions. The extent of structural variation and its effect on interspecific hybridization is unknown in these trees. As a first step towards elucidating the extent of structural variation between the genomes of *E. grandis* and *E. urophylla*, we sequenced and assembled the haplogenomes contained in an F1 hybrid of the two species.

**Findings:**

Using Nanopore sequencing and a trio-binning approach, we assembled the separate haplogenomes (566.7 Mb and 544.5 Mb) to 98.0% BUSCO completion. High-density SNP genetic linkage maps of both parents allowed scaffolding of 88.0% of the haplogenome contigs into 11 pseudo-chromosomes (scaffold N50 of 43.8 Mb and 42.5 Mb for the *E. grandis* and *E. urophylla* haplogenomes, respectively). We identify 48,729 SVs between the two haplogenomes providing the first detailed insight into genome structural rearrangement in these species. The two haplogenomes have similar gene content, 35,572 and 33,915 functionally annotated genes, of which 34.7% are contained in genome rearrangements.

**Conclusions:**

Knowledge of SV and haplotype diversity in the two species will form the basis for understanding the genetic basis of hybrid superiority in these trees.

## Background

There is considerable pressure to improve crop yields to provide food, fibre, shelter and renewable energy for the growing human population [1] in a sustainable manner. Fast-growing *Eucalyptus* tree species provide an important renewable feedstock for biomaterial (timber, fibre and lignocellulosics) and bioenergy production, relieving pressure on native forests [2]. These species, commonly referred to as eucalypts, constitute the most widely planted hardwood fibre crop globally. The most productive plantation areas are planted with interspecific F1 hybrid clones that combine favourable characteristics of parental species and generally lead to increased forest productivity and product quality, and reduced production costs [2, 3]. The most widely planted hybrid combination in subtropical regions, *E. grandis* x *E. urophylla*, is primarily bred to combine the disease resistance of the tropical species *E. urophylla* with the fast growth of the subtropical to temperate species *E. grandis*. To further improve plantation productivity, wood quality and resilience, more efficient breeding strategies have been pursued in the past decade, primarily through genomic selection using genome-wide SNP markers [4, 5].

Discriminating the maternal and paternal chromosome copies (defined by haplotypes or blocks of allelic variants that are inherited together; [6]) allows identification of haplotype and structural variants that may be associated with crop productivity and resilience [7, 8]. Haplotype-based molecular breeding has been shown to be a more accurate and effective breeding strategy [9, 10] compared to SNP based strategies. Haplotypes can often be inferred accurately in offspring by using the parental genomes and previously defined SNP tag-markers to impute haplotypes [11]. SNP tag-markers can then be used in molecular breeding strategies by aiding the selection of progeny for propagation and deployment, or identification of superior parents for further breeding [12].

Access to multiple high-quality reference genome assemblies facilitates the identification of haplotypes and structural variants, both of which underlie pan-genome variation in plants. Genome assembly in highly outbred organisms such as forest trees is often hampered by high levels of heterozygosity and the frequent occurrence of non-syntenic DNA sequences in intergenic regions leading to mixed phase contigs. As a consequence, many of the available reference sequences of outbred plants do not accurately reflect the haplogenomes carried by the reference individuals [13]. Long-read sequencing (LRS) technologies such as Oxford Nanopore (ONT) and Pacific Biosciences (PacBio) can mitigate the challenges associated with assembling outbred plant genomes. Long reads can span across multiple syntenic (gene) regions and connect intergenic haplotypes, allowing separate, phased assembly of haplotype and structural variant alternatives. The growing number of phased genome assemblies, especially those assembled with LRS data, has revealed that a single flat reference genome misses a substantial portion of the genotypic diversity in outbred species [14]. As such, there is a movement towards assembly of pan-reference genomes, which incorporates variants from multiple individuals as has been reported in humans (reviewed by [14]) and plants (reviewed by [15]).

Studies on pan-genomic (including haplotype and structural) variation are still lacking in *Eucalyptus*, with most information on genome synteny still derived from comparative genetic linkage mapping. These studies have suggested high collinearity between eucalypt species, including *E. grandis* and *E. urophylla* [16–19]. However, the degree of fine scale synteny between *E. grandis* and *E. urophylla* is unknown as there is no *de novo* reference assembly available for *E. urophylla*, one of the most important hybrid parent partners. The current reference genome, *E. grandis* v2.0 [20], was sequenced using Sanger sequencing. These technologies have limited capability to resolve haplotype and structural variants (reviewed by [21]). The lack of available LRS based genome assemblies for *E. grandis* and *E. urophylla* have precluded studies of pan-genome variation in these species and their F1 hybrids.

Combining SRS and LRS data with a parent-offspring trio-sequencing approach has been demonstrated to allow assembly of high-quality haplo-reference genomes representing the two parents, at a lower cost than generating two independent reference quality genomes [22–24]. Similarly, trio-sequencing of an interspecific F1 hybrid of *E. grandis* and *E. urophylla*, paired with LRS technologies will generate high-quality assemblies of the haplogenomes contained in the F1 hybrid. Such phased reference genome assemblies will ultimately provide a basis for pursuing haplotype-based molecular breeding of eucalypt trees and will provide insights into the abundance and distribution of structural variants (SVs) of relevance to hybrid genetics breeding. Thus, the aim of this study was to create a starting point for defining pan-genome, haplotype and structural variation in *E. grandis* (NCBI:txid71139), *E. urophylla* (NCBI:txid99020) and their F1 hybrids.

## Methods

### Sample background

**Figure 1:**
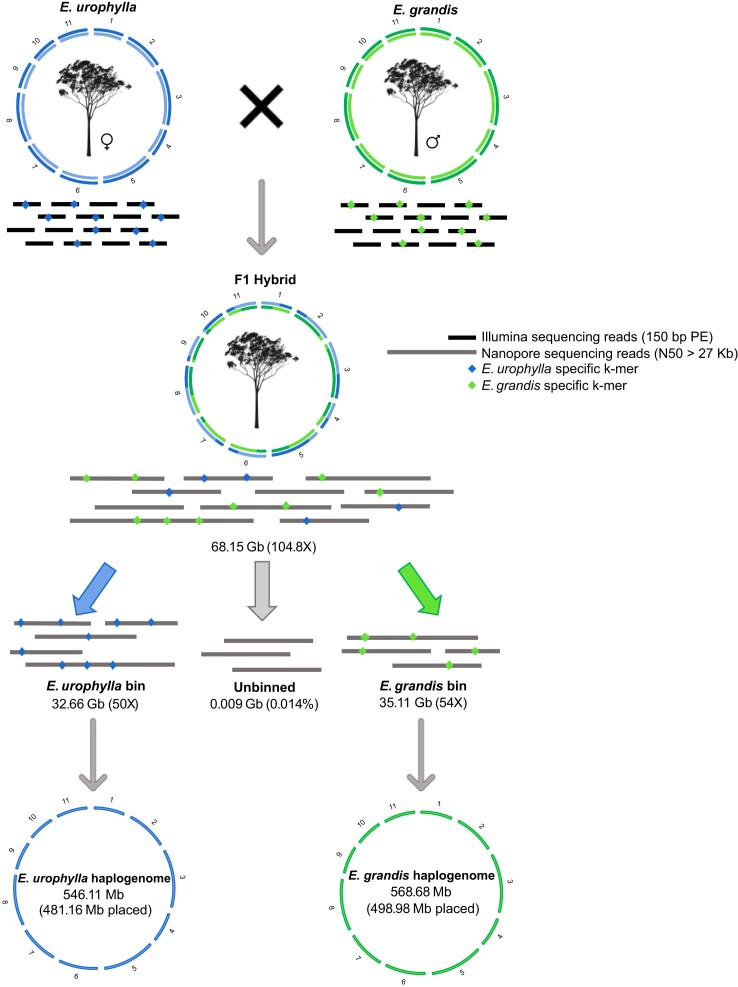
Separate assembly of *E. urophylla* and *E. grandis* haplogenomes in the F1 hybrid using a trio-binning strategy. Using whole-genome Illumina short-read sequencing data of the parental genomes, long-read sequencing data of the F1 hybrid offspring was separated based on unique parental k-mers into *E. urophylla* and *E. grandis* haplotype bins (amount of Nanopore sequencing data is indicated in gigabases (Gb) below each bin, as well as the estimated genome coverage). Reads that contained no unique k-mers were unbinned and kept in a separate bin. Long reads were subsequently assembled independently, resulting in fully assembled *E. urophylla* and *E. grandis* haplogenomes (total assembly size is shown below the relevant haplogenome and size of assembly scaffolded into eleven chromosomes are indicated in brackets). This figure is adapted from [70] and tree images are from [80].

Leaf tissues of an *E. urophylla* x *E. grandis* F1 hybrid individual and its parents (*E. urophylla* seed parent and *E. grandis* pollen parent) were collected and used for DNA extraction. The F1 individual forms part of a large nested association mapping trial and SNP data for the F1 full-sib family was used to generate high-density genetic linkage maps for both parents. Sequencing both parents enables inference of the parental haplotypes inherited by the F1 hybrid through haplotype binning for and phased genome assembly (Fig. 1).

### DNA isolation

#### Illumina sequencing

Genomic DNA was extracted from 50 mg of leaf tissue for the *E. urophylla* and *E. grandis* parents using the NucleoSpin^®^ Plant II Kit (Machery-Nagel, Germany). Gel electrophoresis was performed using a 0.8% w/v agarose gel to assess DNA quality. DNA quality was also assessed using a NanoDrop^®^ ND-1000 spectrophotometer (Thermo Fisher Scientific) and quantified using a Qubit 2.0 Fluorometer (Thermo Fisher Scientific). Whole-genome sequencing of the F1 hybrid and its parents was performed on an Illumina NovaSeq 6000 platform (Illumina NovaSeq 6000 Sequencing System, RRID:SCR_016387) by Macrogen (Macrogen Inc., Seoul, Korea).

#### High molecular weight DNA extraction

HMW genomic DNA was extracted from the F1 hybrid using 1.2 g of flash frozen ground leaf tissue. The ground tissue was suspended in 25 ml Guanidine buffer (20 mM EDTA, 100 mM NaCl, 1% Trition^®^ X-100, 500 mM Guanidine-HCl and 10 mM Tris, pH 7.9), supplemented with 50 mg cellulase (Sigma-Aldrich) and 50 mg lysing enzyme (Sigma-Aldrich) incubated at 42^○^C with gentle agitation. After 2.5 h, 10 μl RNase A (20 μg/ml) was added and the sample was incubated for 30 min at 37^○^C, after which 50 mg proteinase K was added, and the mixture was incubated for another 2 h at 50^○^C. The mixture was then centrifuged for 20 min at 12 000 x g and the clarified lysate transferred to an appropriate buffer QBT-equilibrated QIAGEN Genomic-tip 100/G column (Qiagen), after which the column was washed three times with 7 ml Buffer QC and HMW DNA was eluted with 5 ml Buffer QF. The DNA was precipitated by adding 0.7 V of isopropanol and centrifuged at 12 000 x g for 20 min. The DNA pellet was washed twice with 70% ethanol and resuspended in an appropriate volume of low salt TE (10 mM Tris-HCL pH 8.0; 0.1 mM of EDTA). Gel electrophoresis was performed using a 0.8% w/v agarose gel to assess DNA quality, and DNA quantity was assessed using a Qubit 2.0 Fluorometer (Thermo Fisher Scientific).

#### Nanopore sequencing

HMW DNA of the F1 hybrid was prepared for initial MinION (RRID:SCR_017985) sequencing following the manufacturer’s protocol using the genomic sequencing kit SQK-LSK109 (Oxford Nanopore Technologies, Oxford, UK). Approximately 3.3 μg of HMW DNA was used without exogenous shearing or size selection. HMW DNA was first repaired with NEBNext FFPE Repair Mix (New England Biolabs) and 3^′^-adenylated with NEBNext Ultra II End Repair/dA-Tailing Module (NEB). The DNA was then purified with AMPure XP beads (Beckmann Coulter) and ligated with sequencing adapters (ONT) using NEBNext Quick T4 DNA Ligase (NEB). After purification with AMPure XP beads (Beckman Coulter), the library was mixed with sequencing buffer (ONT) and library loading beads (ONT) and then loaded on primed MinION R9.4 SpotOn flow cells (FLO-MIN106). MinION sequencing was performed with a MinION Mk1B sequencer running for 48 h.

The resulting FAST5 files were base-called and reads with a QV < 7 were removed with Oxford Nanopore Technologies’ Guppy base-calling software v3.4.5 (Guppy basecaller, RRID:SCR_023196, ONT) using parameters for FLO-MIN106 and SQK-LSK109 library type. The Guppy base-caller may not remove all the sequence adapters, so to ensure all sequence adapters are removed Porechop v0.2.4 (Porechop, RRID:SCR_016967) was used. All scripts used in this study are available on GitLab. The resulting adapter-less reads were combined into a single FASTQ file for further use.

PromethION (RRID:SCR_017987) sequencing was performed by the Centre for Genome Innovation (University of Connecticut, Connecticut, USA) on a FLO-PRO002 PromethION flow cell as per the PromethION sequencing protocol (ONT) using the SQK-LSK109 (ONT) sequencing kit after size selection using the Circulomics Short Read Eliminator XS (Circulomics Inc.). The flow cell was washed and reloaded after 38 h and run for an additional 6 h of sequencing. Base-calling was performed using the Guppy v3.4.5 basecaller and adapter removal was performed as described above.

### Genome assembly

#### Trio-binning and haplogenome assembly

K-mer based (21-mer) genome size estimation was performed using llumina short-reads as input for Jellyfish v2.2.6 (Jellyfish, RRID:SCR_005491) [25] and visualised with GenomeScope v2.0 (GenomeScope, RRID:SCR_017014) [26]. Long-reads of the F1 hybrid were binned into *E. urophylla* and *E. grandis* haplotype bins (corresponding to the origin of the parental short-reads) using the Trio-Canu module in Canu v1.8 (Canu, RRID:SCR_015880) [22]. Read contaminants were identified using Centrifuge v1.0.4-beta (Centrifuge Classifier, RRID:SCR_016665) [27] and removed from the binned reads with a custom script. Similarly, contaminant reads were identified and removed from short read data with Kraken v2.0.8-beta (Kraken, RRID:SCR_005484) [28]. The remaining raw reads were used for all assembly and alignment steps.

The binned reads corresponding to each of the parents were assembled separately, along with the corresponding parental short reads, using the MaSuRCA v3.3.4 (MaSuRCA, RRID:SCR_010691) [29] genome assembler. MaSuRCa was chosen as initial testing of multiple genome assemblers (based on the BUSCO completion score, contig N50 and total assembly size) indicated that the MaSuRCA genome assembler performed the best for our data. The quality of the resulting assemblies was assessed using QUAST v5.0.2 (QUAST, RRID:SCR_001228) [30, 31] and BUSCO v5.2.2 (BUSCO, RRID:SCR_015008) using the embryophyta_odb10 library [32–34]. To verify the genome coverage of the assemblies, Illumina reads from each of the parental haplotypes were mapped to the corresponding and alternative assembled haplogenomes using BWA v0.7.5a-r405 (BWA, RRID:SCR_010910) [35] and mapping rate calculated using the flagstat module from Samtools v1.9 (SAMTOOLS, RRID:SCR_002105) [36].

#### Genome scaffolding

To improve assembly contiguity, scaffolding was performed for the MaSuRCa assembled *E. urophylla* and *E. grandis* haplogenomes using high-density SNP genetic linkage maps previously constructed for each of the parents. To resolve possible chimeric contigs that were assembled by MaSuRCa, Polar Star (Polar Star, RRID:SCR_023009) was used to infer breakpoints and split contigs based on identification of read-depth outliers from the binned long-reads. After breakpoints were inferred and contigs split, all contigs smaller than 3 kb were removed. A BLAST database was created for the assembled haplogenomes to identify the position of 1,588 *E. grandis* and 1,575 *E. urophylla* SNP probes used to construct the genetic maps. A consensus map was constructed with ALLMAPS (ALLMAPS, RRID:SCR_021171) [37], consisting of SNPs that mapped to the assembled haplogenomes, to perform genome scaffolding. For the consensus map construction, a weight of two was given to the parental genetic linkage map corresponding to the species haplogenome to be scaffolded, while a weight of one was given for the alternative parental linkage map from the other species. Chromosome scaffold sizes from the two haplogenomes were compared to one another and to that of the *E. grandis* v2.0 genome to see whether the size difference between the haplogenomes and the *E. grandis* v2.0 reference was due to a potential bias in scaffolding of particular chromosomes. To validate if unplaced contigs/scaffolds were from a particular chromosome, unplaced contigs/scaffolds were aligned to the *E. grandis* v2.0 genome using MiniMap2 (RRID:SCR_018550) [38] and alignments visualized with D-Genies (RRID:SCR_018967) [39]. To complement the genome-wide assessment of contiguity and accuracy provided by the BUSCO scores and scaffold N50, we used the LTR assembly index (LAI), to determine the proportion of intact LTR sequences. LAI scores were generated with the LTR_Retriever pipeline (LTR_Retriever, RRID:SCR_017623) [40].

### Genome annotation

Custom libraries of repetitive elements were constructed for the *E. urophylla* and *E. grandis* haplogenomes with RepeatModeler v1.0.8 (RepeatModeler, RRID:SCR_015027) [41]. Repetitive elements were annotated with RepeatMasker v4.0.9 (RepeatMasker, RRID:SCR_012954) [42]. To eliminate the chance of missing repeat elements in either haplogenome, the combined species library was used as input for RepeatMasker. Lastly, to identify the abundance of LTR retrotransposons, LTR retrotransposon candidates were identified with LTR_retriever (RRID:SCR_017623) [43] for both haplogenomes and their distribution visualised with Circos (RRID:SCR_011798) [44].

RNA-Seq reads from previous studies were used for structural genome annotation. RNA-Seq reads used for the *E. grandis* haplogenome assembly were from the original genome assembly paper and included six different tissues from an *E. grandis* individual [45, 46] (all data is available on EucGenIE [47]). For the *E. urophylla* haplogenome, RNA-Seq data from mature leaf and xylem tissues of three-year-old *E. grandis* x *E. urophylla* F1 hybrids backcrossed with *E. urophylla* trees were used (Bioproject: PRJNA354497) [48]. RNA-Seq reads were trimmed with Trimmomatic v0.39 (Trimmomatic, RRID:SCR_011848) [49] and only paired reads were used for further work. Trimmed RNA-Seq reads were aligned to the relevant haplogenome assemblies with Hisat2 v2.1.0 (HISAT2, RRID:SCR_015530) [50]. GenomeThreader v1.7.1 (GenomeThreader, RRID:SCR_023172) [51] was used to align predicted protein sequences from the *E. grandis* v1.0 genome annotation to the haplogenome assemblies. We used BRAKER2 v2.0.5 (BRAKER, RRID:SCR_018964) [52] for structural gene prediction. To predict protein coding regions in the genome, Braker2 first converts RNA-Seq alignments to exon support with GeneMark-ET v4.38 (GeneMark, RRID:SCR_011930) [53]. This output is combined with protein alignments for two rounds of training with AUGUSTUS v3.2.3 (Augustus, RRID:SCR_008417) [54–56]. The predicted gene spaces were then filtered with gFACs v1.1.3 (gFACs, RRID:SCR_022017) [57]. Mono-exonic genes were filtered with InterProScan v5.35-74.0 (InterProScan, RRID:SCR_005829) to keep only those with known protein domains. Completeness of the structural annotations were assessed with BUSCO v5.2.2.

Functional genome annotation was performed with EnTAP v0.9.0 (EnTAP, RRID:SCR_023010) [58] using the following public databases: NCBI RefSeq complete and EMBL-EBI UniProt. This pipeline integrates similarity search and other annotation resources including gene family (eggNOG), protein domains (Pfam), gene ontology and KEGG pathway assignment. To validate proposed structural gene models, coding sequences and peptide sequences were extracted from the haplogenome assembly using the relevant GFF3 annotation file with gffead v0.12.7 (gffread, RRID:SCR_018965) [59] after gene synteny analyses. Genes with a premature stop codon were truncated to the stop codon, and sequences shorter than 30 amino acids were removed.

### Structural variant identification

To check for regions that were not assembled in the haplogenome assemblies compared to the *E. grandis* v2.0 reference genome, the *E. grandis* and *E. urophylla* haplogenomes were each aligned to the *E. grandis* v2.0 reference genome, with MiniMap2 [38] and alignments visualised using D-Genies [39]. Using the same method, the eleven assembled *E. grandis* and *E. urophylla* chromosomes were aligned to each other to visually identify genomic regions with possible large structural variants (SVs). We identified structural rearrangements (inversions, translocations and duplications) and local variations (SNPs, InDels, copy gains/losses, highly diverged regions and tandem repeats) between *E. grandis* and *E. urophylla*, by aligning the haplogenome assemblies to each other using nucmer from the MUMmer3 toolbox (MUMmer, RRID:SCR_018171) [60] with alignment parameters “—maxmatch –c 100 -b 500 -l 50”. The resulting alignments were further filtered for alignment length (>100) and identity (>90). Identification of structural rearrangements and local variations was performed using the Synteny and Rearrangement Identifier (SyRI) pipeline (SyRI, RRID:SCR_023008) [61]. The same method was also used to identify regions that differed between the *E. grandis* haplogenome and the *E. grandis* v2.0 reference genome. As the linear visualisation of syntenic regions and variants from SyRI prohibits us from depicting inter-chromosomal events, synteny and structural variants of greater than 10 kb were visualised with Circos.

Syntenic gene pairs were identified in the *E. grandis* and *E. urophylla* haplogenomes using a python version of MCScan, JCVI v1.1.18 (jcvi, RRID:SCR_021641) [62]. Coding sequence and annotation gff3 files were used as input data to identify the syntenic blocks for each pair of species with the ‘jcvi.compara.catalog ortholog’ command and a c-score parameter of –cscore=0.95. Syntenic blocks were filtered with ‘jcvi.compara.synteny screen’ with parameters –minspan=30 –simple. The pattern of synteny was detected with jcvi.compara.synteny depth –histogram. Smaller syntenic blocks were also filtered with ‘jcvi.compara.synteny screen’ with parameters –minspan=10 –simple. Genes within inverted and translocated syntenic blocks that spanned ten or more gene pairs were checked for gene ontology and Kyoto Encyclopedia of Genes and Genome (KEGG, [63]) enrichment terms using OmicsBox v3.0.29 (OmicsBox, RRID:SCR_023676) [64] and results were visualized using Tableau Professional Edition (Tableau Desktop, RRID:SCR_013994, Tableau Software Inc., Seattle, WA, USA). This was repeated for genes that did not have a pairwise gene alignment.

## Results

### Genome sequencing

Illumina sequencing of an F1 hybrid individual (SAP_F1_FK118) and its pure-species *E. grandis* (SAP_GRA_FK1758) and *E. urophylla* (SAP_URO_FK1756) parents (Sappi Forests Research, Planning and Nurseries, South Africa) resulted in more than 116 Gb of PE150 data per individual (Supplementary Table S1). Using GenomeScope2.0, we estimated the genome size to be 443.2 Mb, 482.3 Mb and 477.8 Mb for the *E. urophylla*, *E. grandis* parents and the F1 hybrid respectively (Supplementary Figure S1). These short read-based estimates were substantially smaller than previous estimates based on flow cytometry [65] and the reported size of *E. grandis* reference genome [20]. Recently, [66] reported a lower flow cytometry size estimate (497.7 Mb) for *E. grandis* supporting our findings. Levels of heterozygosity in the short-read data were 2.1%, 2.6% and 3.5% for the *E. grandis*, *E. urophylla* and the F1 hybrid (Supplementary Figure S1) providing ample genetic diversity for trio-binning of the long-reads (see below).

A total of 75.3 Gb of Nanopore sequencing data was generated (read N50 ∼27 kb), of which 68.2 Gb (90.5%) passed QC (Q-value > 7, Supplementary Table S2) and was used for trio-binning corresponding to ∼104.8X coverage of the F1 hybrid genome and ∼50X coverage per haplogenome (Fig. 1, Supplementary Table S2).

### Genome assembly

#### Phased hybrid genome assembly using trio-binning

To separately assemble the long reads originating from the two haplogenomes in the F1 hybrid, we performed trio-binning using the Illumina short-read data for the parents and the long-read data for the F1 individual. We were able to bin 1,876,816 long reads (32.7 Gb) for the *E. urophylla* haplogenome and 1,998,860 long reads (35.1 Gb) for the *E. grandis* haplogenome corresponding to 50.3X and 54.0X coverage of the two haplotypes, respectively (Fig. 1, Supplementary Table S3). Only 6,693 reads (0.014%) could not be binned and were excluded from further analyses.

Assembly of the binned reads for the *E. urophylla* haplogenome resulted in 654 contigs and a total size of 546.1 Mb, with a contig N50 of 4.4 Mb (Table 1). A BUSCO completeness score of 98.0% was obtained of which 94.7% were single-copy genes and only 3.3% were duplicate-copy genes (Supplementary Figure S2). The reads binned for the *E. grandis* haplogenome assembled into 793 contigs with a total size of 568.5 Mb and a contig N50 of 3.9 Mb (Table 1). For this assembly we obtained a BUSCO completeness score of 98.2%, of which 93.6% were single copy genes and 4.6% were duplicate genes (Supplementary Figure S2). The low duplicate percentages reflected efficient trio-binning and haplogenome assembly. In addition, the LAI score for *E. urophylla* and *E. grandis* was 18.1 and 20.6, respectively, which is similar to other reference and gold level genome assemblies [40] further validating the high quality of the haplogenome assemblies.

**Table 1:** Assembly and annotation statistics for the *E. urophylla* and *E. grandis* haplogenomes compared to the previous *E. grandis* reference genome assembly v2.0 [20]

	*E. grandis* v2.0	*E. grandis*	*E. urophylla*
Type of sequencing	Whole genome shotgun + BAC end Sanger (ABI)	Illumina + ONP	Illumina + ONP
Genome coverage^a^	6.73x	54.01x (ONP)	50.25x (ONP)
Primary assembly:			
Number of contigs	32,724	793	654
Total number bases in contigs	691.43 Mb	568.46 Mb	546.11 Mb
Contig N50 length	67.25 kb	3.91 Mb	4.41 Mb
Contig L50	2,261	38	36
Total contigs >50 kb	288	387	368
Validated contigs (Polar_Star):			
Number of contigs	-	1,579	1,418
Total number bases in contigs	-	566.72 Mb	544.51 Mb
Contig N50 length	-	2.42 Mb	1.93 Mb
Contig L50	-	74	83
Total contigs >50 kb	-	522	547
Assembly BUSCO completeness^b^	98.00%	98.30%	98.00%
Number scaffolds	4,951	1,279	1,078
Total number of bases scaffolded^c^	612.60 Mb	498.98 Mb	481.16 Mb
Scaffold N50	53.80 Mb	43.82 Mb	42.45 Mb
Scaffold L50	5	6	6
BUSCO completeness^d^	98.00%	98.30%	98.00%
GC content	39.99%	39.46%	39.44%
Repeat content	44.50%	49.06%	48.34%
LAI scores	-	20.55	18.06
Number of genes	36,376	39,837	37,933
Annotation BUSCO completeness^e^	99.10% (*v1.0*), 93.8% (*v2.0*)	94.60%	95.80%

^a^ Coverage based on 650 Mb genome size for *E. grandis* and *E. urophylla*.

^b^ BUSCO completeness scores of contig level assembly.

^c^ Total number of bases scaffolded onto one of the eleven chromosomes.

^d^ BUSCO completeness scores of all scaffolds (including unplaced scaffolds).

^e^ BUSCO completeness of gene annotation of the v1.0 [20], v2.0 [19] and haplogenome assemblies.

Next, we mapped the parental Illumina reads to the corresponding haplogenome to investigate whether the smaller than expected haplogenome assembly size might be due to unassembled genomic regions. We observed mapping rates of 98.7% and 99.1% (93.8% and 92.9% properly paired), respectively (Supplementary Table S3), suggesting that it is unlikely that major genomic regions are missing in the haplogenome assemblies.

#### Genome scaffolding

To curate incorrectly assembled contigs, contig breakpoints were inferred based on long-read depth support and used to split suspicious contigs before scaffolding. The parental genetic linkage maps yielded a set of 3,125 ( *E. urophylla* haplogenome) and 3,129 (*E. grandis* haplogenome) unique SNP markers to anchor contigs into pseudo-chromosome level scaffolds. The anchoring rate for both haplogenome assemblies was greater than 88.0% (Table 2) and a BUSCO completeness score of at least 95.3% was obtained for the anchored contigs. Dot-plot visualization of the haplogenome alignment confirmed high levels of collinearity between the assembled haplogenomes (Supplementary Figure S3 and Supplementary Figure S4). ALLMAPS was able to orientate 299 *E. urophylla* and 262 *E. grandis* contigs with two or more markers each, while 52 contigs for *E. urophylla* and 49 for *E. grandis* only had one marker and were placed without orientation (Table 2). A total of 1,067 contigs (corresponding to 63.4 Mb) of the *E. urophylla* and 1,268 contigs (67.8 Mb) of the *E. grandis* haplogenome assembly could not be anchored (Table 2) of which 863 (9.7 Mb) and 1,051 contigs (11.9 Mb) were smaller than 50 kb (Supplementary Table S4) and contained none of the mapped SNP markers.

**Table 2:** Summary statistics for parental linkage maps (gra_allmap and uro_allmap) and final consensus anchoring of the *E. urophylla* and *E. grandis* haplogenome contigs. A greater weight (indicated with w) was given to the linkage map of the species corresponding to the haplogenome being scaffolded. Scaffolds that contain no SNP markers or had ambiguous placements were counted as unplaced. Marker density (measured as number of markers per Mb) represents the sum of unique markers in the two linkage maps

*E. urophylla*	gra_allmap (w=1)	uro_allmap (w=2)	Anchored	Unplaced
Linkage Groups	11	11	11	n.a.
Markers (unique)	1,577	1,573	3,125	25
Average markers per Mb	3.5	3.5	6.5	0.4
N50 Scaffolds	76	79	81	2
Scaffolds	311	299	351	1,067
Scaffolds with 1 marker	83	80	52	13
Scaffolds with 2 markers	51	53	42	4
Scaffolds with 3 markers	41	37	44	0
Scaffolds with > = 4 markers	136	129	213	1
**Total bases**	**448,984,013**	**447,297,011**	**481,132,251**	**63,374,165**
Percent of genome	82.5%	82.1%	88.4%	11.6%
***E. grandis***	**gra_allmap (w=2)**	**uro_allmap (w=1)**	**Anchored**	**Unplaced**
Linkage groups	11	11	11	n.a.
Markers (unique)	1,588	1,575	3,129	34
Average markers per Mb	3.3	3.4	6.3	0.5
N50 Scaffolds	72	72	73	1
Scaffolds	283	263	311	1,268
Scaffolds with 1 marker	62	60	49	21
Scaffolds with 2 markers	46	33	26	3
Scaffolds with 3 markers	32	32	30	1
Scaffolds with > = 4 markers	143	138	206	1
**Total bases**	**477,075,775**	**464,179,728**	**498,948,047**	**67,775,781**
Percent of genome	84.2%	81.9%	88.0%	12.0%

The anchored assembly had a high level of congruence between the genetic and physical maps as indicated by the Pearson’s correlation coefficient (ρ) being close to −1 or 1, with the weakest correlation being ρ = 0.965 (Supplementary Figure S5) for *E. urophylla* and ρ = 0.938 for *E. grandis* (Supplementary Figure S6). Chromosome 3 and 5 differed from the *E. grandis* v2.0 reference genome by more than 20 Mb (Supplementary Figure S7). This could not be explained by a single missing genomic segment (Supplementary Figure S3). To investigate this, we aligned all unplaced scaffolds to the *E. grandis* v2.0 reference genome but did not observe any chromosomal preference for unplaced scaffolds (Supplementary Figure S8). This suggested that the chromosomal size differences were not due to scaffolds not being anchored to those chromosomes (Supplementary Figure S8).

### Genome annotation

**Figure 2:**
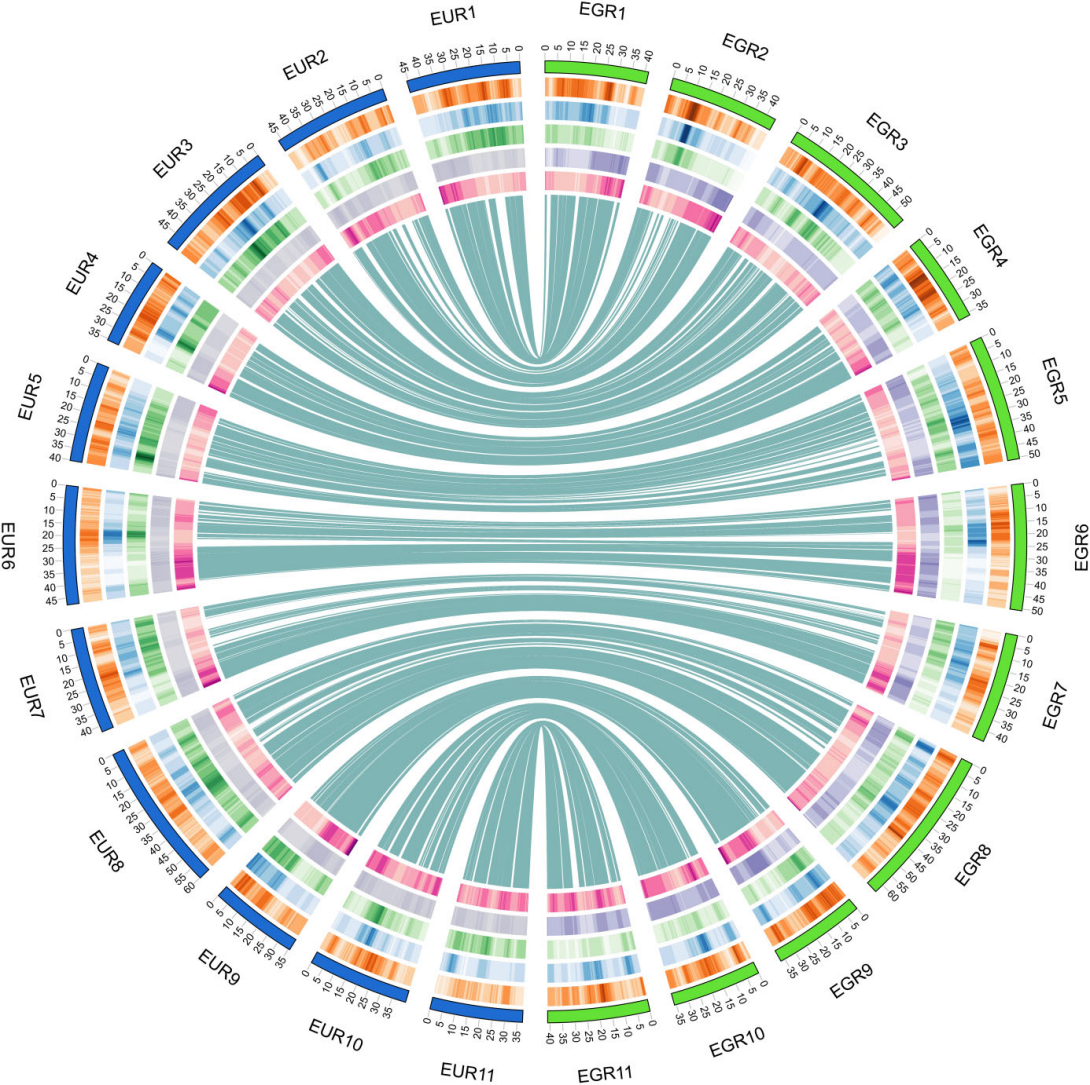
Synteny and distribution of LTR retrotransposons along the *E. grandis* and *E. urophylla* haplogenome assemblies for eleven scaffolded chromosomes. Syntenic regions are shown between the *E. urophylla* and *E. grandis* haplogenomes in the middle, based on SyRI (see Supplementary Figure S9). LTR retrotransposon distribution is shown for the *E. urophylla* (EUR) and the *E. grandis* (EGR) haplogenome assemblies. From outside to inside, the heatmaps show the distribution of Copia (orange, ranging from 6 to 21.5%), Gypsy (blue, ranging from 1.3 to 26.5%) and unknown (green, ranging from 2.8 to 16.6%) LTR retrotransposons, GC% (37.0 to 43.0%) and gene density (0 to 60.0%) with darker shades representing a higher percentage of retrotransposons within the bin. Chromosome number and size is indicated on the outer circle in megabases.

To further examine whether the smaller than expected haplogenome assembly size is due to a difference in repeat content, we annotated repeat elements with RepeatMasker. A total of 48.3% of the *E. urophylla* haplogenome assembly was comprised of repetitive elements, whereas it was 49.1% for the *E. grandis* haplogenome (Supplementary Table S5). In both cases, LTR retrotransposons were the most prevalent repetitive element, making up more than 21% of the assembled haplogenomes (Supplementary Table S5). DNA transposons comprised ∼6% of the haplogenomes. These results are similar to previous repeat annotations for the v2.0 *E. grandis* reference assembly [20] (Table 1). We used LTR retriever to visualize the distribution of various LTR retrotransposon types (in bins of 300 kb, Fig. 2). LTR retriever, which is more sensitive for detection of LTR retrotransposons than RepeatModeler, identified 29.1% and 29.3% of the *E. grandis* and *E. urophylla* haplogenomes respectively, as LTR retrotransposons. Direct comparison of the LTR retrotransposon distribution pattern between *E. grandis* and *E. urophylla* was not possible as the assembled chromosomes differ in size, but there was good relative conservation in pattern with few notable exceptions e.g., on Chromosome 2 (Fig. 2).

Structural (*de novo*) annotation resulted in 39,849 and 37,942 gene models for the *E. grandis* and *E. urophylla* haplogenomes, respectively (Table 1 and Supplementary Table S6). BUSCO completeness scores of 94.6% and 95.8% were obtained for the *E. grandis* and *E. urophylla* structural annotation models (Table 1). Validation of the final GFF3 file revealed sequences with in-frame stop codons within 119 and 96 *E. grandis* and *E. urophylla* predicted genes, respectively. Those with fewer than 30 amino acids were removed (Supplementary Table S13). Functional annotation based on similarity searches or gene family assignment was possible for 35,572 and 33,915 structural gene models of *E. grandis* and *E. urophylla* (Supplementary Table S6).

### Structural variant analysis

**Figure 3:**
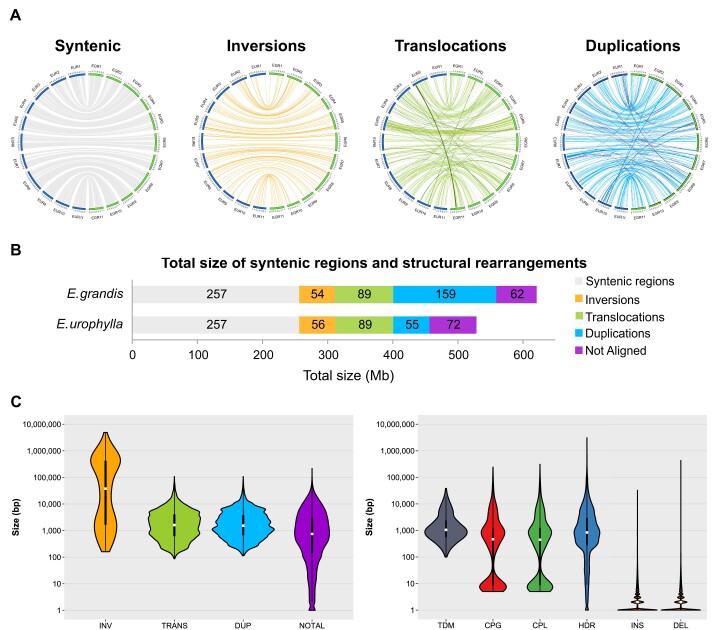
Size and distribution of structural rearrangements and local variants between the *E. grandis* and *E. urophylla* haplogenomes. (A) Distribution of syntenic regions and structural variants between the *E. grandis* and *E. urophylla* haplogenome assemblies. Links are shown between *E. urophylla* (EUR) chromosomes in blue and and *E. grandis* (EGR) chromosomes in green. Only variants of greater than 10 kilobases as identified by SyRI are shown. Darker links show change in chromosome number between EUR and EGR. (B) Total size of syntenic and rearranged regions in megabases (Mb) for the *E. grandis* and *E. urophylla* haplogenome (see Supplementary Table S7 and Supplementary Table S8). The size of syntenic or rearranged regions are indicated within the bar in Mb, while the bar colour represents the rearrangement type. (C) Size distribution of rearranged regions (left) and local variants (right) between the *E. grandis* and *E. urophylla* haplogenomes. Size is indicated in base pairs on the y-axis (ranging from one to 4.91 Mb for rearrangements and one to 3.09 Mb for local variants), and the rearrangement type on the x-axis; INV are inversions, DUP are duplications, TRANS are translocations, NOTAL are regions that are not aligned, TDM are tandem repeats, CPG and CPL are copy gains/losses, HDR are highly diverged regions, INS are insertions and DEL are deletions.

*E. grandis* and *E. urophylla* are in the same section (*Latoangulatae*) and subgenus *Symphyomyrtus* but have non-overlapping natural ranges with unique adaptations such as greater resistance to fungal pathogens in *E. urophylla*, which has a more tropical distribution. Genetic linkage mapping has suggested high collinearity of their genomes [18, 19, 67], but a direct fine-scale comparison of genome synteny between these species has not been possible. Using the SyRI whole-genome comparison tool, we revealed that a total of 256.8 Mb was syntenic between the two haplogenome assemblies, while 262.2 and 374.9 Mb were identified as rearranged in the *E. grandis* and *E. urophylla* haplogenomes, respectively (Figs 2–3, Supplementary Table S7, Supplementary Figure S9). In comparison, 317.7 Mb was syntenic between the *E. grandis* haplogenome and the *E. grandis* v2.0 reference genome (Supplementary Table S7, Supplementary Figure S9), but due to the difference in overall assembly size and methods used in the two studies, it is not possible to compare the genomic proportions. The regions rearranged between the haplogenomes included 189 inversions and 10,526 translocations (Fig. 3, Supplementary Figure S9, Supplementary Table S7, Supplementary Table S9 and Supplementary Table S10). In addition, there were 16,865 duplications in the *E. grandis* and 21,149 duplications in the *E. urophylla* haplogenome (Fig. 3, Supplementary Figure S9 and Supplementary Table S7). Together these results suggest that despite high collinearity previously reported for these species and observed here for the *E. grandis* and *E. urophylla* haplogenomes, extensive fine-scale rearrangements exist that have not been detected in previous studies.

Next, we investigated genome sequence divergence in syntenic regions, designated as “local variants” by SyRI, comprising 65.3 Mb and 66.4 Mb in the *E. grandis* and *E. urophylla* haplogenomes, respectively. These local variants (excluding SNPs) ranged from 1 bp (indels) to 3.1 Mb (highly diverged regions, HDR, Fig. 3 C). SNPs were the most prevalent class of local variants in terms of number, with 8.4 million SNPs between the *E. grandis* and *E. urophylla* haplogenomes, followed by small insertions and deletions (Supplementary Table S8). In terms of the total bases affected, highly diverged regions and copy gain/losses made up 9.6 Mb and 38.1–40.2 Mb of the haplogenome assemblies. Although there are a greater number of local variants compared to SVs, local variants made up 13.8% of the *E. urophylla* and 13.1% of the *E. grandis* chromosomal assembly compared to 54.5% and 75.1% in SV. This suggests that although local variants are more numerous, structural variants have a larger impact on genome architecture. This was also revealed in similar studies in tomato [8] and grape [68].

**Figure 4:**
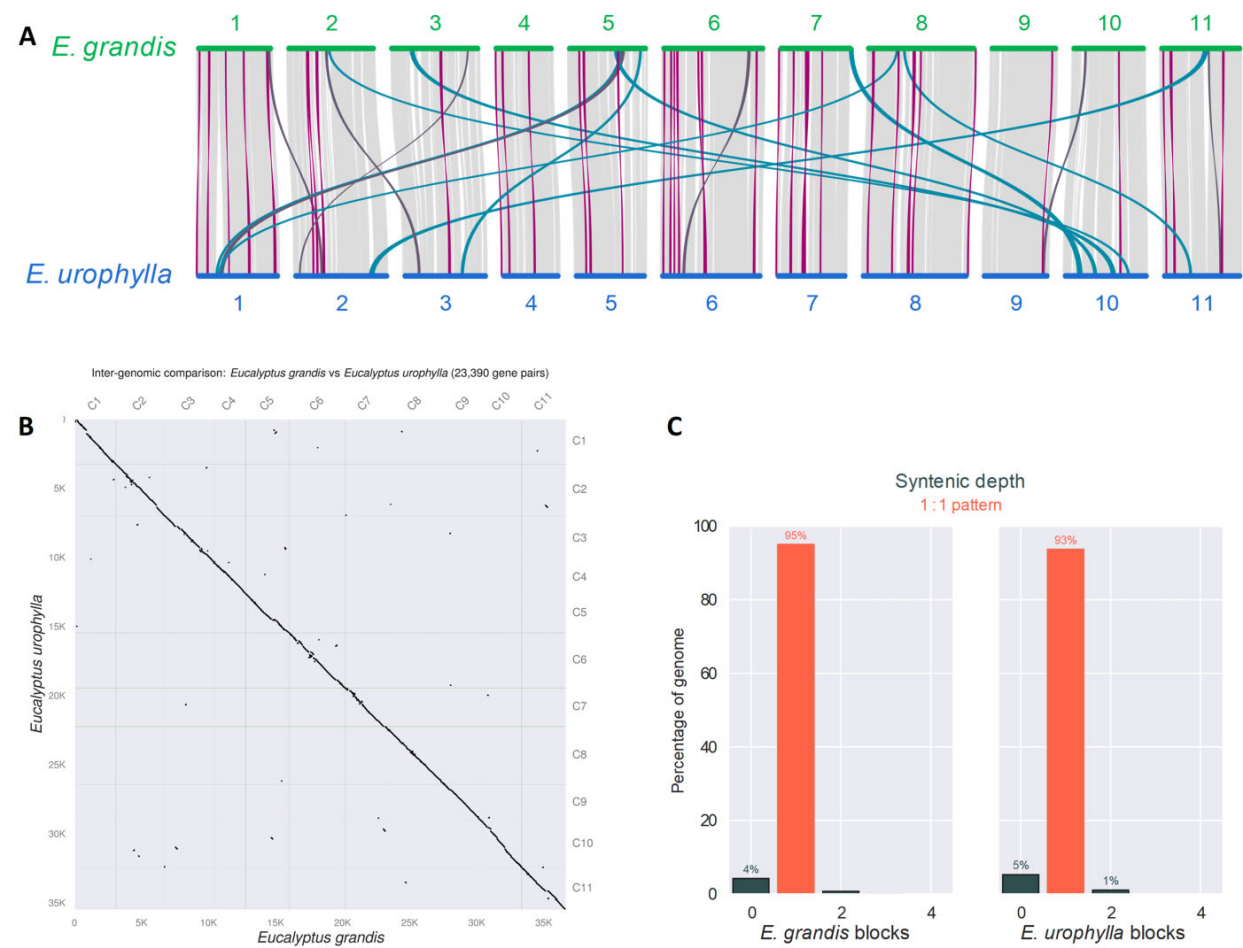
Gene synteny between *E. grandis* and *E. urophylla* haplogenome assemblies. (A) Chromosome-scale collinearity between *E. grandis* and *E. urophylla* haplogenome annotations. Lines in light grey indicate syntenic gene blocks, lines in purple indicate inverted gene blocks, blue indicated translocated gene block and dark grey inverted translocated gene blocks. Only blocks that span greater than 30 gene pairs are shown. (B) Dot-plot alignments of 23,390 gene pairs between the *E. grandis* and *E. urophylla* haplogenome annotations. (C) Bar graph showing syntenic depth of *E. grandis* and *E. urophylla* syntenic blocks. The majority of genes are in a 1 to 1 synteny pattern. All of the graphs were produced in MCScan JCVI v1.1.18 [62].

We performed gene-based synteny analysis between the *E. grandis* and *E. urophylla* haplogenomes, which confirmed high collinearity between the haplogenomes, with 23,390 gene pairs in 238 syntenic blocks (average 98.3 gene pairs per syntenic block with min = 4 gene pairs and max = 1296 gene pairs, Fig. 4). A total of 227 blocks had 10 or more homologous gene pairs and 175 blocks had 30 or more gene pairs derived from the two haplogenomes. Of the 227 blocks, 86 blocks (8,114 genes and 37.9% of gene synteny blocks) are rearranged between the haplogenomes as inversions or translocations. The top GO enrichment terms for genes within these blocks belonged to regulation of transcription, anatomical structure development, DNA binding, transcription regulator activity and RNA binding (Supplementary Figure S10 and Supplementary Figure S11). KEGG pathway analyses indicated enrichment of ribosomal pathway genes in both haplogenomes. The *E. grandis* haplogenome also has enrichment for genes involved in the Glycosaminoglycan biosynthesis - chondroitin sulfate / dermatan sulfate pathway (Supplementary Table S11). For genes that did not have a pairwise alignment (11,130 for *E. grandis* and 10,077 for *E urophylla*), multiple enriched GO terms were found (Supplementary Figure S12), but no significant enriched KEGG pathway genes were found (Supplementary Table S12).

## Discussion

We have assessed the use of a trio-binning strategy to assemble high-quality haplogenomes in an F1 hybrid of two important eucalypt tree species as a starting point towards investigating pan-genome variation within and between these species. The high level of heterozygosity in the F1 hybrid enabled discrimination of almost all parental long reads and independent assembly of the two parental haplogenomes. These haploid assemblies, the first of their kind for a forest tree species, allowed us to circumvent the problem of co-assembly of alternative haplotypes which has presented a challenge for the assembly of highly heterozygous tree genomes, especially in intergenic DNA where complex structural variants from partially overlapping haplotypes may be co-assembled into a mosaic sequence [19, 20]. Furthermore, the high coverage of long reads (50X per haplogenome) and the long-read length (N50 >27 kb) allowed us to assemble across complex repeat structures leading overall to highly contiguous assemblies (contig N50 of 2.4 Mb for *E. grandis* and 1.9 Mb for *E. urophylla*). Intriguingly, we find that, despite having very high BUSCO completeness scores (>98.0%), the assembled haplogenomes (566.7 Mb and 544.5 Mb) were substantially smaller than the previous diploid reference genome assembly of 691.4 Mb [19, 20] and the ∼640 Mb flow cytometry estimate [65]. High-density SNP genetic linkage maps enabled further improvement of haplogenome assembly contiguity (scaffold N50 >42.5 Mb). Finally, we performed the first fine-scale structural and gene-based comparison for any two eucalypt genomes and show that SVs are more prevalent than detected in previous studies, but follow a similar class distribution pattern as in other plants with inversion events the least frequent, followed by translocation events and duplications being the most frequent [61, 69].

### Trio-binning of a highly heterozygous F1 hybrid genome

The trio-binning strategy [70] allowed successful discrimination of the long reads derived from the *E. urophylla* and *E. grandis* haplogenomes. A total of 99.98% of the sequenced read bases could be assigned to one of the two haplo-bins, with only a small proportion (0.014%) of mostly shorter nanopore reads not assigned to bins (N50 = 1,385 bp for un-binned vs N50 ∼27.5 kb for binned reads). The long-read data was split 51.80% vs 48.18% for *E. grandis* and *E. urophylla*, respectively (Supplementary Table S3), matching the assembly sizes, but it is not clear whether this can be generalized for individuals of the two species. Stringent cross-mapping of the parental short-read data to the two haplogenomes revealed, as expected, lower mapping rates to the opposite haplogenome (average 93.4% vs 84.9%, Supplementary Table S3) supporting that we have efficiently separated the haplogenome reads from the two species. The low level of BUSCO duplication in the assembled haplogenomes (less than 4.0%; Supplementary Figure S2) compared to 13.9% reported for a recent *E. pauciflora* assembly [71], supports that the haplotype binning was highly efficient. We further validated the size of phased blocks, as well as phase origin (Supplementary Note 1) and found that the haplogenome assemblies had very low haplotype switch error rates (lower than 0.033%) confirming the accuracy of haplotype separation. Together these results suggest that the trio-binning approach was highly efficient and accurate in the heterozygous F1 hybrid genome.

Haplotype separation is known to improve with higher levels of heterozygosity [70, 72]. We observed high heterozygosity for both pure-species parents (2.1% for *E. grandis* and 2.6% for *E. urophylla*), and as expected, heterozygosity was substantially higher in the F1 hybrid offspring (estimated to be 3.5%; Supplementary Figure S1). Such high heterozygosity levels are expected for outcrossed organisms such as eucalypts [73, 74]. Successful haplotype separation of an F1 hybrid of species within the same section of Myrtaceae (*Latoangulatae*) suggests that application of trio-binning for haplotype separation should be successful for most other viable *Eucalyptus* F1 hybrid combinations. In addition, the high heterozygosity observed in the pure species parents suggests that haplotype binning will also be successful in intraspecific crosses of *Eucalyptus* as the trio-binning strategy has been demonstrated to be efficient at much lower levels of heterozygosity (0.9% in the case of a F1 Brahman x Angus cattle hybrid and 1.4% for *A. thaliana*; [70]).

We note that the haplogenome assembly sizes, 546.1/481.2 Mb for *E. urophylla* and 568.5/498.9 Mb for *E. grandis* (total/scaffolded size) were much smaller than that of the current *E. grandis* v2.0 reference genome (691.4/612.6 Mb, [19, 20]) and previous estimates (∼640 Mb) based on flow cytometry [65]. K-mer based genome size estimates of the parental reads predicted diploid genome sizes of 443.2 Mb for *E. urophylla*, 482.3 Mb for *E. grandis* and 477.8 Mb for the F1 hybrid (Supplementary Figure S1), which agreed with the scaffolded genome sizes of the two haplogenome assemblies. This apparent discrepancy was also observed in *E. pauciflora*, where k-mer based estimates were 408.2 Mb compared to the final 594.9 Mb assembly [71]. The total assembly sizes of the two haplogenomes were therefore approximately 70 - 100 Mb smaller than previous flow cytometry estimates for the two species and the total scaffolded sizes were 140 - 160 Mb smaller than expected. This size discrepancy may be explained by several factors, which we explore below.

First, to exclude the possibility that the smaller assembly size was due to a portion of sequencing reads not being assembled, i.e., that we failed to assemble parts of the haplogenomes, we aligned the parental Illumina reads to the corresponding parental haplogenome assembly. We also aligned the raw short- and long-reads and the haplogenome assemblies to the *E. grandis* v2.0 reference genome to make sure all v2.0 genomic regions had sequencing coverage (Supplementary Note 2). This revealed that some regions had very high sequencing depth relative to the *E. grandis* v2.0 reference genome (Supplementary Note 2) presumably due to highly repetitive sequence content in those regions. More than 98.7% of parental Illumina reads aligned to their corresponding parental haplogenome, which suggests that almost all of the sequences in the parental genomes (that are amenable to Illumina sequencing) are represented in the haplogenomes (Supplementary Table S2), although it is possible that the regions with high sequencing depth represent repetitive regions that are collapsed in the haplogenome assemblies. To further investigate this possibility, we confirmed that the repeat content of the haplogenomes was not lower than that reported in the *E. grandis* v2.0 diploid reference assembly. In fact, the repeat content for the *E. urophylla* and *E. grandis* haplogenomes (48.3% and 49.1%, respectively, Table 1) was higher than that reported for the *E. grandis* v2.0 assembly (44.5%, [20]) and for the more recent *E. pauciflora* assembly (44.8%, [71]). This suggests that the observed size difference is most probably not due to the collapse of repetitive regions during haplogenome assembly. Rather, the slightly higher repeat content of our haplogenome assemblies probably reflect our ability to better assemble across such repeats using long-read technology in haplo-assemblies vs short-read/Sanger sequencing previously used for these highly heterozygous genomes. Previous size estimates were probably somewhat inflated in size due to the possible co-assembly of partially overlapping alternative haplotypes in highly heterozygous regions distributed throughout the genome. Our analysis showed that Chromosomes 3 and 5 in the haplogenome assemblies were 20 Mb smaller than the corresponding chromosomes in the diploid *E. grandis* v2.0 assembly.

### SNP Genetic linkage maps support high scaffolding rates

Overall, 88.4% and 88.0% of the haplogenome assemblies were anchored into 11 pseudo-chromosomes for *E. urophylla* and *E. grandis*. However, there are some limits to using ALLMAPS for genome scaffolding as the program cannot identify and separate duplicated regions that are misassembled or collapsed by the genome assembler due to high similarity [37]. In addition, most genetic linkage maps contain regions such as centromeres with no or very low recombination and few DNA markers for anchoring and orientation of contigs. Many of the unanchored contigs may contain difficult to assemble, centromeric or other non-recombinogenic regions devoid of mapped DNA markers (average 0.4 and 0.5 markers per Mb for unanchored vs 6.5 and 6.3 markers per Mb for anchored *E. urophylla* and *E. grandis* contigs, respectively, Table 2). The N50 of the unanchored contigs was 324 kb, which was smaller than the average marker spacing in those regions (Supplementary Table S4). Thus, integration of additional proximity ligation or optical mapping data may lead to inclusion of some of the remaining unplaced contigs that had few markers to place or orient them. Despite this limitation we were able to produce eleven pseudo-chromosome scaffolds for each of the haplogenomes owing to the high density of SNP markers in the parental maps and the quality of the genetic maps as evidenced by the high collinearity of markers between the genetic map and the *de novo* assembled contigs, as well as high collinearity between the scaffolded assembly and the genetic linkage maps (Pearson’s correlation of ρ = 0.938 to ρ = 1.00; Supplementary Figure S5 and Supplementary Figure S6).

### Structural variants between *E. urophylla* and *E. grandis*

To our knowledge, this is the first genome-wide comparison of synteny and structural rearrangements between *E. urophylla* and *E. grandis*. In addition, we had the advantage of being able to directly compare the two haplogenomes from the same F1 hybrid individual assembled using the same method. Using SyRI we found that 53.4% (256.9 Mb) of the 481.2 Mb chromosomal assembly of *E. urophylla* and 51.5% (256.8 Mb) of the 498.97 Mb chromosomal assembly of *E. grandis* was syntenic (Supplementary Table S7). We were able to identify 48,729 SVs between the two haplogenomes, with a 103.6 Mb difference between the two haplogenomes due to duplications (Supplementary Table S7). As seen in previous studies using SyRI for SV calling, we found that inversions were the smallest group of SVs in terms of number, followed by translocations, with duplications being the most abundant (189 inversions, 10,526 translocations and 38,014 duplications, Supplementary Table S7; [61, 69]). The unfolded site frequency spectrum of SVs [68] suggested that there is purifying selection against SVs, and that there is stronger purifying selection against inversions and translocations compared to duplications as they have a more deleterious effect compared to duplications [68]. Stronger purifying selection against inversions and translocations in our haplogenome assemblies may therefore explain the lower frequency of these two classes of SV, however this will need to be tested in future sequencing projects including population-wide tracking of SVs.

With additional genome sequences for *E. grandis* and *E. urophylla*, a pan-genome reference assembly could be constructed as was done for *Arabidopsis* [69] and tomato [8, 75]. SyRI identifies SVs and local variants using three main steps: 1) identify syntenic alignments, 2) identify inverted, duplicated and translocated alignments and 3) identify “local variants” within alignment blocks. As such, there is a hierarchy of variation where local variants are found within alignment blocks, be they syntenic or rearranged regions. However, when looking for the functional effects of local and larger structural variants, it is important to note the hierarchy of genomic rearrangements, as local variants within rearranged regions show different inheritance patterns to those in syntenic regions. SVs can influence recombination as rearrangement hotspots typically have lower synteny and reduced recombination rates [69]. In addition, SVs can influence gene expression directly or indirectly making their functional interpretation harder [61].

### Smaller than expected haplogenome assembly size

Surprisingly, despite the high completeness, we found that the total assembled size of each of the haplogenomes was substantially smaller than that of the *E. grandis* v2.0 reference genome and previous flow cytometry estimates. We propose that the size difference is not due to collapse of the repeat content of the haplogenome assemblies, but rather due to possible overestimation of the *E. grandis* v2.0 genome assembly size as a result of inclusion of partially overlapping alternative haplotypes in highly heterozygous regions of the diploid genome assembly. However, resolving this discrepancy will require further *de novo* genome assemblies for *E. grandis*, possibly including resequencing using long read technology to update the genome assembly of the reference BRASUZ1 individual, as has been performed for some reference genomes that were originally assembled with Sanger sequencing data [76].

## Conclusions

We have produced phased, reference quality haplogenome assemblies of an interspecific F1 hybrid using a trio-binning approach and performed the first genome-wide analysis of genome synteny between two key tree species used in hardwood plantation forestry, *E. grandis* and *E. urophylla*. This revealed a large number of previously undescribed genome structural variants as a step towards understanding genome structural evolution in this iconic genus of fast-growing woody perennials. The haplogenome resource data provides the insights into haplotype diversity in F1 hybrids and, with additional haplogenomes to be sequenced, this will lead to a better understanding of the genetic basis of hybrid compatibility and superiority. This work is a pilot study towards understanding the nature of pan-genome variation in *Eucalyptus* that can be used for tree improvement. The project also produced the first near complete genome assembly for *E. urophylla*, a key tropical eucalypt with an interesting island colonization history.

## Availability of source code and requirements

- Project name: eucalyptus haplogenome synteny
- Project home page: https://gitlab.com/Anneri/eucalyptus-haplogenome-synteny
- Operating system(s): Platform independent
- Programming language: Bash
- Other requirements: numerous packages described in the Methods section
- License: MIT

## Supplementary Material

giad064_GIGA-D-22-00250_Original_Submission

giad064_GIGA-D-22-00250_Revision_1

giad064_GIGA-D-22-00250_Revision_2

giad064_Response_to_Reviewer_Comments_Original_Submission

giad064_Response_to_Reviewer_Comments_Revision_1

giad064_Reviewer_1_Report_Original_Submission Chao Bian -- 11/10/2022 Reviewed

giad064_Reviewer_1_Report_Revision_1 Chao Bian -- 2/22/2023 Reviewed

giad064_Reviewer_2_Report_Original_Submission Xupo Ding -- 11/16/2022 Reviewed

giad064_Reviewer_2_Report_Revision_1 Xupo Ding -- 3/1/2023 Reviewed

giad064_Supplemental_Files

## Data Availability

Illumina DNA sequencing data was uploaded at NCBI SRA under BioProject: PRJNA885070. High density genetic linkage maps are available on GitLab [78]. The haplogenome assemblies were uploaded to the NCBI database and can be accessed with accession no. JAOPUP000000000 and JAOPUO000000000. All supporting data such as repeat element libraries, genome annotation files, synteny analyses output files etc. are available in GigaDB [79].

## Additional files

**Supplementary Figure S1**: Genome size estimates for the (A) *E. urophylla*, (B) *E. grandis* and (C) the *E. urophylla* x *E. grandis* F1 hybrid genomes.

**Supplementary Figure S2**: Benchmarking Universal Single-Copy Orthologs (BUSCO) completeness scores for both haplogenome assemblies as well as the currently available *E. grandis* v2.0 reference genome.

**Supplementary Figure S3**: Alignment of placed haplogenome scaffolds to the *E. grandis* v2.0 reference genome.

**Supplementary Figure S4**: Alignment between the *E. grandis* and *E. urophylla* scaffolded haplogenome assemblies.

**Supplementary Figure S5**: Pseudochromosomes of *E. urophylla* haplogenome, reconstructed from two genetic linkage input maps – uro.allmap and gra.allmap, with unequal weights (2 and 1 respectively).

**Supplementary Figure S6**: Pseudochromosomes of *E. grandis* haplogenome, reconstructed from two genetic linkage input maps – gra.allmap and uro.allmap, with unequal weights (2 and 1 respectively).

**Supplementary Figure S7**: Scaffolded chromosome sizes of the *E. grandis* v2.0 and the scaffolded *E. grandis* and *E. urophylla* haplogenome assemblies.

**Supplementary Figure S8**: Alignment of unplaced *E. grandis* and *E. urophylla* haplogenome scaffolds to the *E. grandis* v2.0 reference genome.

**Supplementary Figure S9**: Syntenic and rearranged regions between the *E. grandis* v2.0, *E. grandis* and *E. urophylla* haplogenomes for all eleven chromosomes.

**Supplementary Figure S10**: Enriched gene ontology (GO) terms for inverted and translocated gene alignment blocks of the *E. grandis* haplogenome.

**Supplementary Figure S11**: Enriched gene ontology (GO) terms for inverted and translocated gene alignment blocks of the *E. urophylla* haplogenome.

**Supplementary Figure S12**: Enriched gene ontology (GO) terms genes that did not have pairwise alignment between the *E. grandis* and *E. urophylla* haplogenomes.

**Supplementary Figure S13**: Hap-mer blob plot of the *E. grandis* and *E. urophylla* haplogenome assemblies.

**Supplementary Figure S14**: Evaluation of haplotype phase blocks. All hap-mer information was generated with Merqury v1.1 [72].

**Supplementary Figure S15**: Genome coverage of the *E. grandis* v2.0 nuclear reference and plastid genomes.

**Supplementary Figure S16**: Summary of the total size and type of elements found in high genome coverage bins. Organellar introgression was identified through BLAST analysis to the *E. grandis* plastid genomes [77], while repeat elements were identified with RepeatMasker.

**Supplementary Note 1**: Hap-mer based phasing completeness assessment.

**Supplementary Note 2**: Read and assembly alignment and validation of high peak content

**Supplementary Table S1**: Illumina sequencing results.

**Supplementary Table S2**: Nanopore sequencing results for the F1 hybrid individual.

**Supplementary Table S3**: Summary statistics for long-read binning using the parental short reads.

**Supplementary Table S4**: Summary statistics of placed and unplaced contigs after scaffolding with ALLMAPS for the *E. urophylla* and *E. grandis* haplogenomes respectively.

**Supplementary Table S5**. Repeat element content of assembled haplogenomes.

**Supplementary Table S6**: Haplogenome annotation statistics.

**Supplementary Table S7**: Number and total length of syntenic and rearranged regions in the *E. grandis* and *E. urophylla* haplogenomes.

**Supplementary Table S8**: Number and total length of local sequence variation in syntenic and rearranged region in the *E. grandis* and *E. urophylla* haplogenomes.

**Supplementary Table S9**: Inversions larger than 50 kb between the *E. grandis* and *E. urophylla* haplogenomes.

**Supplementary Table S10**: Translocations between the *E. grandis* and *E. urophylla* haplogenomes that are larger than 50 kb.

**Supplementary Table S11**: KEGG pathway enrichment analyses for genes within inverted and translocated gene alignment blocks between the *E. grandis* and *E. urophylla* haplogenome assemblies.

**Supplementary Table S12**: KEGG pathway enrichment analyses for genes that do not have a pairwise alignment between the *E. grandis* (reference) and *E. urophylla* (test) haplogenome assemblies.

**Supplementary Table S13**: Altered position and length of genes with an in-frame stop codon.

**Supplementary Table S14**: Phase block statistics of the *E. grandis* and *E. urophylla* haplo-genome assemblies.

**Supplementary Table S15**: *E. grandis* and *E. urophylla* high coverage bin content.

## List of abbreviations

BAC: bacterial artificial chromosome; BLAST: Basic Local Alignment Search Tool; BUSCO: Benchmarking Universal Single-Copy Orthologs; BWA: BurrowsWheeler Aligner; CDS: coding sequence; Chr: Chromosome; DUP: duplication; EGR: *E. grandis*; EUR: *E. urophylla*; FDR: false discovery rate; Gb: Gigabase; GO: gene ontology; HDR: highly diverged regions; HMW: high molecular weight; INV: inversion; kb: kilobase; KEGG: Kyoto Encyclopedia of Genes and Genomes; LINE: long interspersed nucleotide element; LRS: Long-read sequencing; LTR: long terminal repeat; Mb: megabase; NCBI: National Center for Biotechnology Information; ONT: Oxford Nanopore Technologies, PacBio: Pacific Biosciences; PE: paired-end; QC: quality control; QUAST: quality assessment tool; QV: quality value; RNA-seq: RNA sequencing; SNP: single nucleotide polymorphism; SRA: Sequence Read Archive; SRS: short read sequencing; SVs: structural variants; SYN: syntenic region; SyRI: Synteny and Rearrangement Identifier; TE: transposable element; TRANS: translocation.
