# Supplementary material for: Haplogenome assembly reveals structural variation in Eucalyptus interspecific hybrids
Source: Gigascience. 2023 Aug 26;12:giad064. doi: 10.1093/gigascience/giad064 (PMC10460159; doi:10.1093/gigascience/giad064)
Supplement: giad064_GIGA-D-22-00250_Original_Submission [file giad064_giga-d-22-00250_original_submission.pdf]

GigaScience

Haplogenome assembly reveals interspecific structural variation in Eucalyptus hybrids

--Manuscript Draft--

**Manuscript Number:**	GIGA-D-22-00250
**Full Title:**	Haplogenome assembly reveals interspecific structural variation in Eucalyptus hybrids
**Article Type:**	Data Note
**Funding Information:**	Department of Science and Innovation, South Africa — Prof. Alexander Myburg<br>South African Forestry Sector Innovation Fund (FSIF) — Prof. Alexander Myburg<br>National Research Foundation (MND190406427887) — Ms Anneri Lotter<br>Technology Innovation Agency (TIA) South Africa — Prof. Alexander Myburg<br>Sappi South Africa — Prof. Alexander Myburg
**Abstract:**	Background: De novo phased (haplo)genome assembly using long-read DNA sequencing data has improved the detection and characterization of structural variants (SVs) in plant and animal genomes. Able to span across haplotypes, long reads allow phased, haplogenome assembly in highly outbred organisms such as forest trees. Eucalyptus tree species and interspecific hybrids are the most widely planted hardwood trees with F1 hybrids of Eucalyptus grandis and E. urophylla forming the bulk of fast-growing pulpwood plantations in subtropical regions. The extent of structural variation and its effect on interspecific hybridization is unknown in these trees. As a first step towards elucidating the extent of structural variation between the genomes of E. grandis and E. urophylla, we sequenced and assembled the haplogenomes contained in an F1 hybrid of the two species. Findings: Using Nanopore sequencing and a trio-binning approach, we assembled the separate haplogenomes (567 Mb and 545 Mb) to 97.9% BUSCO completion. High-density SNP genetic linkage maps of both parents allowed scaffolding of 88% of the haplogenome contigs into 11 pseudo-chromosomes (scaffold N50 of 43.82 Mb and 42.45 Mb for the E. grandis and E. urophylla haplogenomes, respectively). We identify 48,729 SVs between the two haplogenomes providing the first detailed insight into genome structural rearrangement in these species. The two haplogenomes have similar gene content, 35,572 and 33,915 functionally annotated genes, of which 34% are contained in genome rearrangements. Conclusions: Knowledge of SV and haplotype diversity in the two species will form the basis for understanding the genetic basis of hybrid superiority in these trees.
**Corresponding Author:**	Alexander Myburg<br>University of Pretoria<br>Pretoria, Gauteng SOUTH AFRICA
**Corresponding Author Secondary Information:**	
**Corresponding Author's Institution:**	University of Pretoria
**Corresponding Author's Secondary Institution:**	
**First Author:**	Anneri Lotter
**First Author Secondary Information:**	
**Order of Authors:**	Anneri Lotter<br>Julia Candotti<br>Tuan A. Duong<br>Eshchar Mizrachi<br>Jill L. Wegrzyn

Powered by Editorial Manager® and ProduXion Manager® from Aries Systems Corporation

	Alexander Myburg
**Order of Authors Secondary Information:**	
**Additional Information:**	
**Question**	**Response**
Are you submitting this manuscript to a special series or article collection?	No
**Experimental design and statistics**<br><br><br>Full details of the experimental design and statistical methods used should be given in the Methods section, as detailed in our Minimum Standards Reporting Checklist. Information essential to interpreting the data presented should be made available in the figure legends.<br><br><br>Have you included all the information requested in your manuscript?	Yes
**Resources**<br><br><br>A description of all resources used, including antibodies, cell lines, animals and software tools, with enough information to allow them to be uniquely identified, should be included in the Methods section. Authors are strongly encouraged to cite Research Resource Identifiers (RRIDs) for antibodies, model organisms and tools, where possible.<br><br><br>Have you included the information requested as detailed in our Minimum Standards Reporting Checklist?	Yes
**Availability of data and materials**<br><br><br>All datasets and code on which the conclusions of the paper rely must be either included in your submission or deposited in publicly available repositories (where available and ethically appropriate), referencing such data using a unique identifier in the references and in	Yes

the “Availability of Data and Materials” section of your manuscript.<br><br>Have you have met the above requirement as detailed in our Minimum Standards Reporting Checklist?	

LaTeX - Main Document (TeX file)

Click here to access/download;LaTeX - Main Document (TeX file);Haplogenome assembly in Eucalyptus hybrids.tex

Placeholder for journal logo gigascience-logo.pdf

GigaScience, 2022, 1–11

doi: xx.xxxx/xxxx
Manuscript in Preparation
Paper

PAPER

Haplogenome assembly reveals interspecific structural variation in *Eucalyptus* hybrids

Anneri Lötter[1], Tuan A. Duong[1], Julia Candotti[1], Eshchar Mizrachi[1], Jill L. Wegrzyn[2] and Alexander A. Myburg[1,*]

[1]Department of Biochemistry, Genetics and Microbiology, Forestry and Agricultural Biotechnology Institute (FABI), University of Pretoria, Private bag X20, Pretoria 0028, South Africa and [2]Department of Ecology and Evolutionary Biology, Institute for Systems Genomics: Computational Biology Core, University of Connecticut, 67 N. Eagleville Road, Storrs, Connecticut, USA

[*]**corresponding author:** zander.myburg@fabi.up.ac.za

Abstract

Background: *De novo* phased (haplo)genome assembly using long-read DNA sequencing data has improved the detection and characterization of structural variants (SVs) in plant and animal genomes. Able to span across haplotypes, long reads allow phased, haplogenome assembly in highly outbred organisms such as forest trees. *Eucalyptus* tree species and interspecific hybrids are the most widely planted hardwood trees with F1 hybrids of *Eucalyptus grandis* and *E. urophylla* forming the bulk of fast-growing pulpwood plantations in subtropical regions. The extent of structural variation and its effect on interspecific hybridization is unknown in these trees. As a first step towards elucidating the extent of structural variation between the genomes of *E. grandis* and *E. urophylla*, we sequenced and assembled the haplogenomes contained in an F1 hybrid of the two species. **Findings:** Using Nanopore sequencing and a trio-binning approach, we assembled the separate haplogenomes (567 Mb and 545 Mb) to 97.9% BUSCO completion. High-density SNP genetic linkage maps of both parents allowed scaffolding of 88% of the haplogenome contigs into 11 pseudo-chromosomes (scaffold N50 of 43.82 Mb and 42.45 Mb for the *E. grandis* and *E. urophylla* haplogenomes, respectively). We identify 48,729 SVs between the two haplogenomes providing the first detailed insight into genome structural rearrangement in these species. The two haplogenomes have similar gene content, 35,572 and 33,915 functionally annotated genes, of which 34% are contained in genome rearrangements. **Conclusions:** Knowledge of SV and haplotype diversity in the two species will form the basis for understanding the genetic basis of hybrid superiority in these trees.

Key words: *Eucalyptus*; trio-binning; phased genome assembly; Nanopore; structural variant

Background

There is considerable pressure to improve crop yields to provide food, fibre, shelter and renewable energy for the growing human population [1] in a sustainable manner. Fast-growing *Eucalyptus* tree species provide an important renewable feedstock for biomaterial (timber, fibre and lignocellulosics) and bioenergy production, relieving pressure on native forests [2]. These species, commonly referred to as eucalypts, constitute the most widely planted hardwood fibre crop globally. The most productive plantation areas are planted with interspecific F1 hybrid clones that combine favourable characteristics of parental species and generally lead to increased forest productivity and product quality, and reduced production costs [2, 3]. The most widely planted hybrid combination in subtropical regions, *E. grandis* x *E. urophylla*, is primarily bred to combine the disease resistance of the tropical species *E. urophylla* with the fast growth of the subtropical species *E. grandis*. To further improve plantation productivity, wood quality and resilience, more efficient breeding strategies have been pursued in the past decade, primarily through genomic selection using genome-wide SNP markers [4, 5].

Discriminating the maternal and paternal chromosome copies (defined by haplotypes or blocks of allelic variants that are inherited together; [6]) allows for identification of haplo-

Compiled on: October 25, 2022.
Draft manuscript prepared by the author.

type and structural variants that may be associated with crop productivity and resilience [7, 8]. Haplotype-based molecular breeding has been shown to be a more accurate and effective breeding strategy [9, 10] compared to SNP based strategies. Haplotypes can often be inferred accurately in offspring by using the parental genomes and previously defined SNP tag-markers and haplotype imputation with statistical methods [11]. SNP tag-markers can then be used in accelerated breeding strategies by aiding the selection of progeny for propagation and deployment, or identification of parents for further breeding [12].

Access to multiple high-quality reference genome assemblies facilitates the identification of haplotypes and structural variants, both of which underlie pan-genome variation in plants. Genome assembly in highly outbred organisms such as forest trees, is often hampered by high levels of heterozygosity and the frequent occurrence of non-syntenic DNA sequences in intergenic regions leading to mixed phase contigs. As a consequence, many of the available reference sequences of outbred plants do not accurately reflect the haplogenomes carried by the reference individuals [13]. Long-read sequencing (LRS) technologies such as Oxford Nanopore (ONT) and Pacific Biosciences (PacBio) can mitigate the challenges associated with assembling outbred plant genomes. Long reads can span across multiple syntenic (gene) regions and connect intergenic allelic variants between them, allowing separate, phased assembly of haplotype and structural variant alternatives. The growing number of phased genome assemblies, especially those assembled with LRS data, has revealed that a single flat reference genome misses a substantial portion of the genotypic diversity in outbred species [14]. As such, there is a movement towards assembly of a pan-reference genome, which incorporates variants from multiple individuals as has been reported in humans (reviews by [14]) and plants (reviewed by [15]).

Studies on pan-genomic (including haplotype and structural) variation are still lacking in *Eucalyptus*, with most information on genome synteny still derived from genetic linkage mapping. These studies have suggested that there is high collinearity between eucalypt species, including *E. grandis* and *E. urophylla* [16, 17, 18, 19] . However, the degree of fine scale synteny between *E. grandis* and *E. urophylla* is unknown as there is not a *de novo* reference assembly available for *E. urophylla*, one of the most important hybrid parent partners. The current reference genome, *E. grandis* [20], was sequenced with a combination of Sanger and SRS. These technologies have limited capability to identify haplotype and structural variants (reviewed by [21]). The lack of available LRS based genome assemblies for *E. grandis* and *E. urophylla* have precluded studies of pan-genome variation in these species and their F1 hybrids.

Combining SRS and LRS data with a parent-offspring trio-sequencing approach has been demonstrated to allow assembly of high-quality haplo-reference genomes for the two parents, at a lower cost than generating two independent reference quality genomes[22, 23, 24]. Similarly, trio-sequencing of an interspecific F1 hybrid of *E. grandis* and *E. urophylla*, paired with LRS technologies will generate high-quality assemblies of the haplogenomes contained in the F1 hybrid. These high-quality phased genome assemblies will ultimately provide a basis for pursuing haplotype-based molecular breeding of eucalypt trees and will provide preliminary insights into the abundance and distribution of structural variants (SVs) of consequence to breeding. Thus, the aim of this study was to create a starting point for defining pan-genome, haplotype and structural variation in *E. grandis* and *E. urophylla*.

Methods

Sample background

Leaf tissues of an F1 *E. urophylla* x *E. grandis* hybrid offspring and its parents (*E. urophylla* seed parent and *E. grandis* pollen parent) were collected and used for DNA extractions. These individuals form part of a large nested association mapping trial and SNP data was used to generate high-density genetic linkage maps for both the *E. grandis* and *E. urophylla* parents (Candotti *et al.*). Sequencing both parents will enable a) inference of both haplotypes for the parental genomes and b) haplotype binning for genome phasing (Figure 1).

DNA Isolation

Illumina sequencing

Genomic DNA was extracted from 50 mg of leaf tissue for the *E. urophylla* and *E. grandis* parents using the NucleoSpin® Plant II Kit (Machery-Nagel, Germany). Gel electrophoresis was performed using a 0.8% w/v agarose gel to assess DNA quality. DNA quality was also assessed using a NanoDrop® ND-1000 spectrophotometer (Thermo Fisher Scientific) and quantified using a Qubit 2.0 Fluorometer (Thermo Fisher Scientific). Whole-genome sequencing of the F1 hybrid and its parents was performed on an Illumina NovaSeq6000 platform (Illumina NovaSeq 6000 Sequencing System, RRID:SCR_016387) by Macrogen (Macrogen Inc., Seoul, Korea).

High molecular weight DNA extraction

Genomic DNA was extracted using 1.2 g of flash frozen ground leaf tissue. The ground material was suspended in 25 ml Guanidine buffer (20 mM EDTA, 100 mM NaCl, 1% Trition® X-100, 500 mM Guanidine-HCl and 10 mM Tris, pH 7.9), supplemented with 50 mg cellulase (Sigma-Aldrich) and 50 mg lysing enzyme (Sigma-Aldrich) incubated at 42 °C with gentle agitation. After 2.5 h, 10 µl RNase A (20 µg/ml) was added and the sample was incubated for 30 min at 37 °C, after which 50 mg proteinase K was added, and the mixture was incubated for another 2 h at 50 °C. The mixture was then centrifuged for 20 min at 12 000 x g and the clarified lysate transferred to an appropriate buffer QBT-equilibrated QIAGEN Genomic-tip 100/G column (Qiagen), after which the column was washed three times with 7 ml Buffer QC and HMW DNA was eluted with 5 ml Buffer QF. The DNA was precipitated by adding 0,7 V of isopropanol and centrifuged at 12 000 g for 20 min. The DNA pellet was washed twice with 70% Ethanol and resuspended in an appropriate volume of low salt TE (10 mM Tris-HCL pH 8.0; 0.1 mM of EDTA). Gel electrophoresis was performed using a 0.8% w/v agarose gel to assess DNA quality, and DNA quantity was assessed using a Qubit 2.0 Fluorometer (Thermo Fisher Scientific).

Nanopore sequencing

HMW DNA was prepared for MinION (RRID:SCR_017985) sequencing following the manufacturers protocol using the genomic sequencing kit SQK-LSK109 (Oxford Nanopore Technologies, Oxford, UK). Approximately 3.3 µg of HMW DNA from was used without exogenous shearing or size selection. HMW DNA was first repaired with NEBNext FFPE Repair Mix (New England Biolabs) and 3'-adenylated with NEBNext Ultra II End Repair/dA-Tailing Module (NEB). The DNA was then purified with AMPure XP beads (Beckmann Coulter) and ligated with sequencing adapters (ONT) using NEBNext Quick T4 DNA Ligase (NEB). After purification with AMPure XP beads (Beckman Coulter), the library was mixed with sequencing buffer (ONT) and library loading beads (ONT) and loaded on primed MinION R9.4 SpotOn flow cells (FLO-MIN106). MinION sequencing was performed with a MinION Mk1B sequencer running for 48 h.

The resulting FAST5 files were base-called and reads with a QV < 7 were removed with Oxford Nanopore Technologies'

Guppy base-calling software v3.4.5 (ONT) using parameters for FLO-MIN106 and SQK-LSK109 library type. RStudio was used summarise and visualise statistics for both DNA isolation methods based on the sequencing summary file generated by the Guppy base-caller. The Guppy base-caller may not remove all the sequence adapters so to ensure all sequence adapters are removed Porechop v0.2.4 (Porechop, RRID:SCR_016967) was used. All scripts used in this study is available on github. The resulting adapter-less reads of both DNA isolation methods were combined into a single FASTQ file for further use.

PromethION (RRID:SCR_017987) sequencing was performed by the Centre for Genome Innovation (University of Connecticut, Connecticut, USA) on a FLO-PRO002 PromethION flow cell as per the PromethION sequencing protocol (ONT) using the SQK-LSK109 (ONT) sequencing kit with Circulomics Short Read Eliminator XS (Circulomics Inc.) size-selection. The flow cell was washed and reloaded after 38 h and run for an additional 6 h of sequencing. Base-calling was performed using the Guppy v3.4.5 basecaller and adapter removal was performed as stated above.

Genome assembly

Trio-binning and haplogenome assembly

Illumina short-reads were used for k-mer based genome size estimation was performed using Jellyfish v2.2.6 (Jellyfish, RRID:SCR_005491) [25] for 21-mers and visualised with GenomeScope v2.0 (GenomeScope, RRID:SCR_017014) [26]. Long-reads of the F1 hybrid were binned into *E. urophylla* and *E. grandis* haplotype bins (corresponding to parental short-reads) using the Trio-Canu module in Canu v1.8 (Canu, RRID:SCR_015880) [22]. Read contaminants were identified from the binned reads using Centrifuge v1.0.4-beta (Centrifuge Classifier, RRID:SCR_016665) [27] and removed with a custom script. Similarly, contaminant reads were also identified and removed from short read data with Kraken v2.0.8-beta (Kraken, RRID:SCR_005484) [28]. The remaining raw reads were used for all assembly and alignment steps.

The binned reads corresponding to each of the parents were assembled separately, along with the corresponding parental short reads, using the MaSuRCA v3.3.4 (MaSuRCA, RRID:SCR_010691) [29] genome assembler. MaSuRCa was chosen as initial testing of multiple genome assemblers (based on the BUSCO completion score, contig N50 and total assembly size) indicated that the MaSuRCA genome assembler performed the best. Quality of the resulting assemblies was assessed using QUAST v5.0.2 (QUAST,RRID:SCR_001228) [30, 31] and BUSCO v5.0.0 (BUSCO, RRID:SCR_015008) [32, 33, 34]. To verify genome coverage of the assemblies, Illumina reads from each of the parental haplotypes were mapped to the corresponding and alternative assembled haplogenomes using BWA v0.7.5a-r405 (BWA, RRID:SCR_010910) [35] and mapping rate calculated using the flagstat module from Samtools v1.9 (SAMTOOLS, RRID:SCR_002105) [36].

Genome scaffolding

To improve assembly contiguity, scaffolding was performed for the MaSuRCa assembled *E. urophylla* and *E. grandis* genomes using high-density genetic linkage maps previously constructed for each of the parents (Candotti *et al.*). To resolve possible chimeric contigs that were assembled by MaSuRCa, Polar_Star (`https://github.com/phasegenomics/polar_star`) was used to infer breakpoints and split in contigs based on identification of read-depth outliers from the binned long-reads. After breakpoints were inferred and contigs split, all contigs smaller than 3 kb were removed before scaffolding with high-density genetic linkage maps. A BLAST database was created for both assembled haplogenomes to identify the position of 1,588 *E. grandis* and 1,575 *E. urophylla* SNP probes used to construct the genetic maps. A consensus map was constructed with ALLMAPS (ALLMAPS, RRID:SCR_021171) [37], consisting of SNPs that mapped to the assembled haplogenomes, and was used to perform genome scaffolding. For the consensus map construction, a weight of two was given to the parental genetic linkage map corresponding to species haplogenome to be scaffolded, while a weight of one was given for the alternative parental linkage map. Chromosome scaffold sizes from the two haplogenomes were compared to one another and to the *E. grandis* v2.0 genome to see whether there is a size difference between the *E. grandis* v2.0 reference potential bias in scaffolding of particular chromosomes. To validate if unplaced contigs/scaffolds were from a particular chromosome, unplaced contigs/scaffolds were aligned to the *E. grandis* v2.0 genome using MiniMap2 (RRID:SCR_018550) [38] and alignments visualized with D-Genies (RRID:SCR_018967) [39].

Genome annotation

Custom libraries of repetitive elements were constructed for the *E. urophylla* and *E. grandis* haplogenomes with RepeatModeler v1.0.8 (RepeatModeler, RRID:SCR_015027) [40]. Repetitive elements were annotated with RepeatMasker v4.0.9 (RepeatMasker, RRID:SCR_012954) [41] for the haplogenome assemblies. To eliminate the chance of missing repeat elements in either haplogenome due to them not being identified, the combined species library was used as input for RepeatMasker. Lastly, to identify the abundance of LTR retrotransposons, LTR retrotransposon candidates were identified with LTR_retriever (RRID:SCR_017623) [42] for both haplogenomes and their distribution visualised with Circos (RRID:SCR_011798) [43].

RNA-Seq reads from previous studies were used for structural genome annotation. RNA-Seq reads used for the *E. grandis* haplogenome assembly were from the original genome assembly paper and included six different tissues from an *E. grandis* individual [44, 45] all data is available on `https://eucgenie.org/`. RNA-Seq data from three-year-old *E. grandis* x *E. urophylla* backcrossed with *E. urophylla* trees were used and only derived from two tissues (mature leaf and xylem, Bioproject: `https://www.ncbi.nlm.nih.gov/bioproject/PRJNA354497`) [46]. RNA-Seq reads were trimmed with Trimmomatic v0.39 (Trimmomatic, RRID:SCR_011848) [47] and only paired reads were used for further work. Trimmed RNA-Seq reads were aligned to the relevant haplogenome assemblies with Hisat2 v2.1.0 (HISAT2, RRID:SCR_015530) [48]. GenomeThreader v1.7.1 [49] was used to align protein sequences of the *E. grandis* v2.0 genome annotation to the haplogenome assemblies. We used BRAKER2 v2.0.5 (BRAKER, RRID:SCR_018964) [50] for structural gene prediction. To predict protein coding regions in the genome, Braker2 first converts RNA-Seq alignments to exon support with GeneMark-ET v4.38 (GeneMark, RRID:SCR_011930) [51]. This output is combined with protein alignments for two rounds of training with AUGUSTUS v3.2.3 (Augustus, RRID:SCR_008417) [52, 53, 54]. The predicted gene space was then filtered with gFACs v1.1.3 (gFACs,) [55]. Mono-exonic genes were filtered with InterProScan v5.35-74.0 (InterProScan, RRID:SCR_005829) to keep only those with known protein domains. Completeness of the structural annotations were assessed with BUSCO v5.0.0.

Functional genome annotation was performed with EnTAP v0.9.0 [56] using the following public databases: NCBI RefSeq complete and EMBL-EBI UniProt. This pipeline integrates similarity search and other annotation resources which include gene family (eggNOG), protein domains (Pfam), gene ontology and KEGG pathway assignment.

Structural variant identification

To check for regions that are unassembled in the haplogenome assemblies compared to the *E. grandis* v2.0 reference genome, the *E. grandis* and *E. urophylla* haplogenomes were each aligned to the *E. grandis* v2.0 genome, with MiniMap2 [38] and alignments visualised using D-Genies [39]. Using the same method, the eleven assembled *E. grandis* and *E. urophylla* chromosomes were aligned to each other to visually identify genomic regions with possible large structural variants (SVs). To identify structural rearrangements (inversions, translocations and duplications) and local variations (SNPs, InDels, copy gains/losses, highly diverged regions and tandem repeats) between *E. grandis* and *E. urophylla*, haplogenome assemblies were aligned to each other using nucmer from the MUMmer3 toolbox (MUMmer, RRID:SCR_018171) [57] with alignment parameters "—maxmatch -c 100 -b 500 -l 50" . The resulting alignments were further filtered for alignment length (>100) and identity (>90). Identification of structural rearrangements and local variations was performed using the Synteny and Rearrangement Identifier (SyRI) pipeline [58]. The same method was also used to identify regions that differed between the *E. grandis* haplogenome and the *E. grandis* v2.0 reference genome. As the linear visualisation of syntenic regions and variants from SyRI prohibits us from depicting inter-chromosomal events, synteny and variants of greater than 10 kb were visualised with Circos.

Syntenic gene pairs between the *E. grandis* and *E. urophylla* haplogenomes were identified using a python version of MCScan, JCVI v1.1.18 (jcvi, RRID:SCR_021641) [59]. Coding sequence and annotation gff3 files were used as input data to identify the syntenic blocks for each pair of species with the 'jcvi.compara.catalog ortholog' command and a c-score parameter of –cscore=0.95. Syntenic blocks were filtered with 'jcvi.compara.synteny screen' with parameters –minspan=30 –simple. The pattern of synteny was detected with jcvi.compara.synteny depth –histogram. Smaller syntenic blocks were also filtered with 'jcvi.compara.synteny screen' with parameters –minspan=10 –simple. Genes within inverted and translocated syntenic blocks that spanned ten or more gene pairs were checked for gene ontology enrichment terms using Blast2GO v1.20.14 (Blast2GO, RRID:SCR_005828) [60] and results were visualized using Tableau Professional Edition (Tableau Desktop, RRID:SCR_013994) (Tableau Software Inc., Seattle, WA, USA).

Results

Genome sequencing

Illumina sequencing of an F1 hybrid individual (SAP_F1_FK118) and its pure-species *E. grandis* (SAP_GRA_FK1758) and *E. urophylla* (SAP_URO_FK1756) parents (Sappi Forest Research, South Africa) resulted in more than 116 Gb of PE150 data per individual (Supplementary Table S1). Using GenomeScope2.0, we estimated the genome size to be 443.19 Mb, 482.27 Mb and 477.76 Mb for the *E. urophylla*, *E. grandis* parents and the F1 hybrid respectively (Supplementary Figure S1). These short read-based estimates were substantially smaller than previous estimates based on flow cytometry [61] and that reported for the *E. grandis* reference genome [20]. [62] recently reported a lower flow cytometry size estimate (497.7 Mb) for *E. grandis* supporting our findings. Levels of heterozygosity in the short-read data were 2.14%, 2.63% and 3.46% for the *E. grandis*, *E. urophylla* and the F1 hybrid (Supplementary Figure S1) providing ample genetic diversity for trio-binning of the long-reads (see below).

A total of 75.32 Gb of Nanopore sequencing data was generated (read N50 27 kb), of which 68.15 Gb (90.48%) passed QC (Q-value > 7, Supplementary Table S2) and was used for trio-binning corresponding to 105X coverage of the F1 hybrid genome and 50X coverage per haplogenome (Figure 1, Supplementary Table S2).

Genome assembly

Phased hybrid genome assembly using trio-binning

To separately assemble the long reads originating from the two haplogenomes in the F1 hybrid, we performed trio-binning using the Illumina short-read data for the parents and the long-read data for the F1 individual. We were able to bin 1,876,816 reads (32.66 Gb) for the *E. urophylla* haplogenome and 1,998,860 reads (35.11 Gb) for the *E. grandis* haplogenome corresponding to 50X and 54X coverage of the two haplotypes, respectively (Figure 1, Supplementary Table S3). Only 6,693 reads (0.014%) could not be binned and were excluded from further analyses.

Assembly of the binned reads for the *E. urophylla* haplogenome resulted in 654 contigs and a total size of 546.1 Mb, with a contig N50 of 4.41 Mb 1. A BUSCO completeness score of 97.9% was obtained of which 95.2% were single-copy genes and only 2.7% were duplicate-copy genes (Supplementary Figure S2). The reads binned for the *E. grandis* haplogenome assembled into 793 contigs with a total size of 568.5 Mb and a contig N50 of 3.91 Mb 1. For this assembly we obtained a BUSCO completeness score of 98.3%, of which 94.8% were single copy genes and 3.5% were duplicate genes (Supplementary Figure S2). The low duplicate percentages reflected efficient trio-binning and haplogenome assembly.

Next, we mapped the parental Illumina reads to the corresponding haplogenome to investigate whether the smaller than expected haplogenome assembly size might be due to unassembled genomic regions. We observed mapping rates of 98.73% and 99.10% (93.79% and 92.91% properly paired), respectively (Supplementary Table S3), indicating that it is unlikely that major genomic regions are missing in the haplogenome assemblies.

Genome scaffolding

To curate incorrectly assembled contigs, contig breakpoints were inferred based on long-read depth support and used to split suspicious contigs before scaffolding. The parental genetic linkage maps yielded a set of 3,125 (for the *E. urophylla* haplogenome) and 3,129 (*E. grandis* haplogenome) unique SNP markers to anchor contigs into pseudo-chromosome level scaffolds. The anchoring rate for both haplogenome assemblies was greater than 88.0% 2 and a BUSCO completeness score of at least 95.3% was obtained for contigs anchored to one of the eleven chromosomes. Dot-plot visualization of the haplogenome alignment confirmed high levels of collinearity between the assembled haplogenomes (Supplementary Figure S3 and Supplementary Figure S4). ALLMAPS was able to orientate 299 *E. urophylla* and 262 *E. grandis* contigs with two or more markers each., while 52 contigs for *E. urophylla* and 49 for *E. grandis* only had one marker and were placed without orientation 2. A total of 1,067 contigs (corresponding to 63.37 Mb) of the *E. urophylla* and 1,268 contigs (67.78 Mb) of the *E. grandis* haplogenome assembly could not be anchored 2 of which 863 (9.71 Mb) and 1,051 contigs (11.86 Mb) were smaller than 50 kb (Supplementary Table S4) and contained no markers.

Most of the anchored assembly had a high level of congruence between the genetic and physical maps as indicated by the Pearson's correlation coefficient (ρ) being close to -1 or 1, with the weakest correlation being $\rho = 0.965$ (Supplementary Figure S5) for *E. urophylla* and $\rho = 0.938$ for *E. grandis* (Supplementary Figure S6). Chromosome 3 and 5 differed from the *E. grandis* v2.0 reference genome by more than 20 Mb (Supplementary

Table 1. Assembly and annotation statistics for the *E. urophylla* and *E. grandis* haplogenomess compared to the previous *E. grandis* genome assembly

	E. grandis v2.0	*E. grandis*	*E. urophylla*
Type of sequencing	BAC end cloning (ABI)	Illumina + ONP	Illumina + ONP
Genome coverage[a]	6.73x	54.01x (ONP)	50.25x (ONP)
Primary assembly:			
Number of contigs	32,724	793	654
Total number bases in contigs	691.43 Mb	568.46 Mb	546.11 Mb
Contig N50 length	67.25 kb	3.91 Mb	4.41 Mb
Contig L50	2,261	38	36
Total contigs >50 kb	288	387	368
Validated contigs (Polar_Star):			
Number of contigs	-	1,579	1,418
Total number bases in contigs	-	566.72 Mb	544.51 Mb
Contig N50 length	-	2.42 Mb	1.93 Mb
Contig L50	-	74	83
Total contigs >50 kb	-	522	547
Assembly BUSCO completeness[b]	98.00%	98.30%	97.90%
Number scaffolds	4,951	1,279	1,078
Total number of bases scaffolded[c]	612.60 Mb	498.98 Mb	481.16 Mb
Scaffold N50	53.80 Mb	43.82 Mb	42.45 Mb
Scaffold L50	5	6	6
BUSCO completeness[d]	98.00%	98.30%	97.90%
GC content	39.99%	39.46%	39.44%
Repeat content	44.50%	49.06%	48.34%
Number of genes	36,376	39,849	37,942
Annotation BUSCO completeness[e]	99.10%	94.60%	95.80%

[a] Coverage based on 650 Mb genome size for *E. grandis* and *E. urophylla*.
[b] BUSCO completeness scores of contig level assembly.
[c] Total number of bases scaffolded onto one of the eleven chromosomes.
[d] BUSCO completeness scores of all scaffolds (including unplaced scaffolds).
[e] BUSCO completeness of gene annotation

Table 2. Summary statistics for parental linkage maps (gra_allmap and uro_allmap) and final consensus anchoring of the *E. urophylla* and *E. grandis* haplogenome contigs. A greater weight (indicated with w) was given to the linkage map of the species corresponding to the haplogenome being scaffolded. Scaffolds that contain no SNP markers or had ambiguous placements were counted as unplaced. Marker density (measured as number of markers per Mb) represents the sum of unique markers in the two linkage maps.

E. urophylla	**gra_allmap (w=1)**	**uro_allmap (w=2)**	**Anchored**	**Unplaced**
Linkage Groups	11	11	11	n.a.
Markers (unique)	1,577	1,573	3,125	25
Average markers per Mb	3.5	3.5	6.5	0.4
N50 Scaffolds	76	79	81	2
Scaffolds	311	299	351	1,067
Scaffolds with 1 marker	83	80	52	13
Scaffolds with 2 markers	51	53	42	4
Scaffolds with 3 markers	41	37	44	0
Scaffolds with >=4 markers	136	129	213	1
**Total bases**	**448,984,013**	**447,297,011**	**481,132,251**	**63,374,165**
Percent of genome	82.5%	82.1%	88.4%	11.6%

E. grandis	**gra_allmap (w=2)**	**uro_allmap (w=1)**	**Anchored**	**Unplaced**
Linkage groups	11	11	11	n.a.
Markers (unique)	1,588	1,575	3,129	34
Average markers per Mb	3.3	3.4	6.3	0.5
N50 Scaffolds	72	72	73	1
Scaffolds	283	263	311	1,268
Scaffolds with 1 marker	62	60	49	21
Scaffolds with 2 markers	46	33	26	3
Scaffolds with 3 markers	32	32	30	1
Scaffolds with >=4 markers	143	138	206	1
**Total bases**	**477,075,775**	**464,179,728**	**498,948,047**	**67,775,781**
Percent of genome	84.2%	81.9%	88.0%	12.0%

Figure S7), which could not be explained by a single missing genomic segment (Supplementary Figure S3). To investigate this, we aligned all unplaced scaffolds to the *E. grandis* v2.0 reference genome but did not observe any chromosomal preference for unplaced scaffolds (Supplementary Figure S8). This suggested that the chromosomal size differences were not due to scaffolds not being placed to those chromosomes (Supplementary Figure S8).

Genome annotation

To further examine whether the smaller haplogenome assembly size is due to a difference in repeat content, we annotated repeat elements with RepeatMasker. A total of 48.34% of the *E. urophylla* haplogenome assembly comprised of repetitive elements, whereas it was 49.06% for the *E. grandis* haplogenome (Supplementary Table S5). In both cases, LTR retrotransposons were the most prevalent repetitive element, making up more than 21% of the assembled haplogenomes (Supplementary Table S5). DNA transposons made up 6% of the haplogenomes. These results are similar to previous repeat annotations for the v2.0 *E. grandis* reference assembly [20], Table 1). We used LTR retriever to visualize the distribution of various LTR retrotransposon types (in bins of 300 kb Figure 2). LTR retriever, which is more sensitive for detection of LTR retrotransposons than RepeatModeler, identified 29.08% and 29.25% of the *E. grandis* and *E. urophylla* haplogenomes respectively, as LTR retrotransposons. Direct comparison of the LTR retrotransposon distribution pattern between *E. grandis* and *E. urophylla* was not possible as the assembled chromosomes differ in size, but there was good relative conservation in pattern with few notable exceptions e.g., on Chromosome 2 (Figure 2).

Structural (*de novo*) annotation resulted in 39,849 and 37,942 gene models for the *E. grandis* and *E. urophylla* haplogenomes, respectively (Table 1 and Supplementary Table S6). BUSCO completeness scores of 94.6% and 95.8% was obtained for the *E. grandis* and *E. urophylla* structural annotation models (Table 1). Functional annotation based on similarity searches or gene family assignment was possible for 35,572 and 33,915 structural gene models of *E. grandis* and *E. urophylla* (Supplementary Table S6).

Structural variant analysis

E. grandis and *E. urophylla* are in the same section (*Latoangulatae*) and subgenus *Symphyomyrtus* but have non-overlapping natural ranges with unique adaptations such as greater resistance to fungal pathogens in *E. urophylla*, which has a more tropical distribution. Genetic linkage mapping has suggested high collinearity of their genomes [18, 63, 19], but a direct fine-scale comparison of genome synteny between these species has not been possible. Using the SyRI the whole-genome comparison tool, we revealed that a total of 257 Mb was syntenic between the two haplogenome assemblies, while 262.22 and 374.85 Mb were identified as rearranged in the *E. grandis* and *E. urophylla* haplogenomes, respectively (Figure 2, Figure 3, Supplementary Table S7, Supplementary Figure S9). In comparison, 318 Mb was syntenic between the *E. grandis* haplogenome and the *E. grandis* v2.0 reference genome (Supplementary Table S7, Supplementary Figure S9), but due to the difference in overall assembly size and methods used in the two studies, it is not possible to compare the genomic proportions. The regions rearranged between the haplogenomes included 189 inversions and 10,526 translocations (Figure 3, Supplementary Figure S9, Supplementary Table S7, Supplementary Table S9 and Supplementary Table S10). In addition, there were 16,865 duplications in the *E. grandis* and 21,149 duplications in the *E. urophylla* haplogenome (Figure 3, Supplementary Figure S9 and Supplementary Table S7). Together these results suggest that despite high collinearity previously reported for these species and observed here for the *E. grandis* and *E. urophylla* haplogenomes, extensive fine-scale rearrangements exist that have not been detected in previous studies.

Next, we investigated genome sequence divergence in syntenic regions, designated as "local variants" by SyRI, comprising 65.26 Mb and 66.45 Mb in the *E. grandis* and *E. urophylla* haplogenomes, respectively. These local variants (excluding SNPs) ranged from 1 bp (indels) to 3.09 Mb (highly diverged regions, HDR, Figure 3C). SNPs were the most prevalent class of local variants in terms of number, with 8.3 million SNPs between the *E. grandis* and *E. urophylla* haplogenomes, followed by small insertions and deletions (Supplementary Table S8). In terms of the total bases affected, highly diverged regions and copy gain/losses made up 9.6 Mb and 38 – 40 Mb of the haplogenome assemblies. Although there is a greater number of local variants compared to SVs, local variants made up 13.8% of the *E. urophylla* and 13.1% of the *E. grandis* chromosomal assembly compared to 54.5% and 75.1% in SV. This suggests that although local variants are more numerous, structural variants have a larger impact on genome architecture. This was also revealed in similar studies in tomato[8] and grape [64].

We performed gene-based synteny analysis between the *E. grandis* and *E. urophylla* haplogenomes, which confirmed high collinearity between the haplogenomes, with 23,390 gene pairs in 238 syntenic blocks (average 98.28 gene pairs per syntenic block with min = 4 gene pairs and max = 1296 gene pairs, Figure 4). A total of 227 blocks had 10 or more homologous gene pairs and 175 blocks had 30 or more gene pairs derived from the two haplogenomes. Of the 227 blocks, 86 blocks (8,114 genes and 37.88% of gene synteny blocks) are rearranged between the haplogenomes as inversions or translocations. The top GO enriched terms within these blocks belonged to regulation of transcription, DNA binding transcription factor activity and mRNA binding (a full list of enriched GO terms can be found in Supplementary Figure S10).

Discussion

We have assessed the use of a trio-binning strategy to assemble high-quality haplogenomes in an F1 hybrid of two important eucalypt tree species as a starting point towards investigating pan-genome variation within and between these species. The high level of heterozygosity in the F1 hybrid enabled discrimination of almost all parental long reads and independent assembly of the two parental haplogenomes. These haploid assemblies, the first of their kind for a forest tree species, allowed us to circumvent the problem of co-assembly of alternative haplotypes which has presented a challenge for the assembly of highly heterozygous tree genomes, especially in intergenic DNA where complex structural variants from partially overlapping haplotypes may be co-assembled into a mosaic sequence [20, 19]. Furthermore, the high coverage of long reads (50X per haplogenome) and the long-read length (N50 >27 Kb) allowed us to assemble across complex repeat structures leading overall to highly contiguous assemblies (contig N50 of 2.42 Mb for *E. grandis* and 1.93 Mb for *E. urophylla*). Intriguingly, we find that, despite having very high BUSCO completeness scores (>97.9%), the assembled haplogenomes (567 Mbp and 545 Mbp) were substantially smaller than the previous diploid reference genome assembly of 691.4 Mb [20, 19] and the 640 Mb flow cytometry estimate [61]. High-density SNP genetic linkage maps enabled further improvement of haplogenome assembly contiguity (scaffold N50 >42 Mbp). Finally, we performed the first fine-scale structural and gene-based comparison for any two eucalypt genomes and show that SVs are more prevalent than detected in previous studies, but follow a similar class distribution pattern as in other plants with inversion events the least frequent, followed by translocation events and duplications being the most frequent [58, 65].

Trio-binning of a highly heterozygous F1 hybrid genome

The trio-binning strategy [66] allowed successful discrimination of the long reads derived from the *E. urophylla* and *E. grandis* haplogenomes. A total of 99.98% of the sequenced read bases could be assigned to one of the two haplo-bins, with only a small proportion (0.014%) of mostly shorter nanopore

reads was not assigned to bins (N50 = 1,385 bp for un-binned vs N50 27.5 kb for binned reads). The long-read data was split 51.80% vs 48.18% for *E. grandis* and *E. urophylla*, respectively, Supplementary Table S3), matching the assembly sizes, but it is not clear whether this can be generalized for individuals of the two species. Stringent cross-mapping of the parental short-read data to the two haplogenomes revealed, as expected, lower mapping rates to the opposite haplogenome (average 93.35% vs 84.94%, Supplementary Table S3) supporting that we have efficiently separated the haplogenome reads from the two species. The low level of BUSCO duplication in the assembled haplogenomes (less than 3.3%; Supplementary Figure S2) compared to 13.9% reported for a recent diploid *E. pauciflora* assembly [67], supports that the haplotype binning was highly efficient. We further validated the size of phased blocks, as well as phase origin (Supplementary Note 1) and found that the haplogenome assemblies had very low haplotype switch error rates (lower than 0.033%) confirming the accuracy of haplotype separation. Together these results suggest that the trio-binning approach was highly efficient and accurate in the heterozygous F1 hybrid genome.

Haplotype separation is known to improve with higher levels of heterozygosity [66, 68]. We observed high heterozygosity for both pure-species parents (2.14% for *E. grandis* and 2.63% for *E. urophylla*), and as expected, heterozygosity was substantially higher in the F1 hybrid offspring (estimated to be 3.46%; Supplementary Figure S1). Such high heterozygosity levels are expected for outcrossed organisms such as eucalypts [69, 70]. Successful haplotype separation of an F1 hybrid of species within the same section of Myrtaceae (*Latoangulatae*) suggests that application of trio-binning for haplotype separation should be successful for most other viable *Eucalyptus* F1 hybrid combinations. In addition, the high heterozygosity observed in the pure species parents suggests that haplotype binning will also be successful in intraspecific crosses of *Eucalyptus* as the trio-binning strategy has been demonstrated to be efficient at much lower levels of heterozygosity (0.9% in the case of a F1 Brahman x Angus cattle hybrid and 1.36% for *A. thaliana*; [66]).

We note that the haplogenome assembly sizes, 546/481 Mb for *E. urophylla* and 568/498 Mb for *E. grandis* (total/scaffolded size) were much smaller than that of the current *E. grandis* v2.0 reference genome (691/612 Mb, [20, 19] and previous estimates (640 Mb) based on flow cytometry [61]. K-mer based genome size estimates of the parental reads predicted diploid genome sizes of 443 Mb for *E. urophylla*, 482 Mb for *E. grandis* and 478 Mb for the F1 hybrid (Supplementary Figure S1), which agreed with the scaffolded genome sizes of the two haplogenome assemblies. This apparent discrepancy was also observed in *E. pauciflora*, where k-mer based estimates were 408 Mb compared to the final 595 Mb assembly [67]. The total assembly sizes of the two haplogenomes were therefore approximately 70 - 100 Mb smaller than previous flow cytometry estimates for the two species and the total scaffolded sizes were 140 - 160 Mbp smaller than expected. This size discrepancy may be explained by several factors, which we explore below.

First, to exclude the possibility that the smaller assembly size was due to a portion of sequencing reads not being assembled, i.e., that we failed to assemble parts of the haplogenomes, we aligned the parental Illumina reads to the corresponding parental haplogenome assembly. We also aligned the raw short- and long-reads and the haplogenome assemblies to the *E. grandis* v2.0 reference genome to make sure all v2.0 genomic regions had sequencing coverage (Supplementary Note 2). This revealed that some regions had very high sequencing depth relative to the *E. grandis* v2.0 reference genome (Supplementary Note 2) presumably due to highly repetitive sequence content in those regions. More than 98.7% of parental Illumina reads aligned to their corresponding parental haplogenome, which suggests that almost all of the sequences in the parental genomes (that are amenable to Illumina sequencing) are represented in the haplogenomes (Supplementary Table S2), although it is possible that the regions with high sequencing depth represent repetitive regions that are collapsed in the haplogenome assemblies. To further investigate this possibility, we confirmed that the repeat content of the haplogenomes was not lower than that reported in the *E. grandis* v2.0 diploid reference assembly. In fact, the repeat content for the *E. urophylla* and *E. grandis* haplogenomes (48.34% and 49.06%, respectively, Table 1) was higher than that reported for the *E. grandis* v2.0 assembly (44.50%, [20] and for the more recent *E. pauciflora* assembly (44.77%, [67]). This suggests that the observed size difference is most probably not due to the collapse of repetitive regions during haplogenome assembly. Rather, the slightly higher repeat content of our haplogenome assemblies probably reflect our ability to better assemble across such repeats using long-read technology in haplo-assemblies vs short-read/Sanger sequencing previously used for these highly heterozygous genomes. Previous size estimates were probably somewhat inflated in size due to the possible co-assembly of partially overlapping alternative haplotypes highly heterozygous regions distributed throughout the genome. Our analysis showed that Chromosomes 3 and 5 in the haplogenome assemblies were 20 Mb smaller than the corresponding chromosomes in the diploid *E. grandis* v2.0 assembly.

Genetic linkage maps support high scaffolding rates

Overall, 88.4% and 88.0% of the haplogenome assemblies was anchored into 11 pseudo-chromosomes for *E. urophylla* and *E. grandis*. However, there are some limits to using ALLMAPS for genome scaffolding as the program cannot identify and separate duplicated regions that are misassembled or collapsed by the genome assembler due to high similarity [37]. In addition, most genetic linkage maps contain regions such as centromeres with no or very low recombination and few DNA markers for anchoring and orientation of contigs. Many of the unanchored contigs may contain difficult to assemble, centromeric or other non-recombinogenic regions devoid of mapped DNA markers (average 0.4 and 0.5 markers per Mb for unanchored vs 6.5 and 6.3 markers per Mb for anchored *E. urophylla* and *E. grandis* contigs, respectively, Table 2). The N50 of the unanchored contigs was 324 kb, which was smaller than the average marker spacing in those regions (Supplementary Table S4). Thus, integration of additional proximity ligation or optical mapping data may lead to inclusion of some of the remaining unplaced contigs that had few markers to place or orient them. Despite this limitation we were able to produce eleven pseudo-chromosome scaffolds for each of the haplogenomes owing to the high density of SNP markers in the parental maps and the quality of the genetic maps as evidenced in collinearity of markers between the genetic map and the de novo assembled contigs, as well as high collinearity between the scaffolded assembly and the genetic linkage maps (Pearson's correlation of ρ = 0.938 to ρ = 1.00; Supplementary Figure S5 and Supplementary Figure S6).

Structural variants between *E. urophylla* and *E. grandis*

To our knowledge, this is the first genome-wide comparison of synteny and structural rearrangements between two eucalypt species. In addition, we had the advantage of being able to directly compare the two haplogenomes from the same F1 hybrid individual assembled using the same method. Using SyRI we found that 53.39% (256.88 Mb) of the 481.16 Mb chromosomal assembly of *E. urophylla* and 51.45% (256.75 Mb) of the 498.97 Mb chromosomal assembly of *E. grandis* was syntenic

(Supplementary Table S7). We were able to identify 48,729 SVs between the two haplogenomes, with a 103.62 Mb difference between the two haplogenomes due to duplications (Supplementary Table S7). As seen in previous studies using SyRI for SV calling, we found that inversions were the smallest group of SVs in terms of number, followed by translocations, with duplications being the most abundant (189 inversions, 10,526 translocations and 38,014 duplications, Supplementary Table S7; [58, 65]. Using unfolded site frequency spectrum of SVs, [64] found that there is purifying selection against SVs, and that there is stronger purifying selection against inversions and translocations compared to duplications as they have a more deleterious effect compared to duplications [64]. Stronger purifying selection against inversions and translocations in our haplogenome assemblies may therefore explain the lower frequency of these two classes of SV, however this will need to be tested in future sequencing projects including population-wide tracking of SVs.

With additional genome sequences for *E. grandis* and *E. urophylla*, a pan-genome reference assembly could be constructed as was done for *Arabidopsis* [65] and tomato [8, 71]. SyRI identifies SVs and local variants using three main steps: 1) identify syntenic alignments, 2) identify inverted, duplicated and translocated alignments and 3) identify "local variants" within alignment blocks. As such, there is a hierarchy of variation where local variants are found within alignment blocks, be they syntenic or rearranged regions. However, when looking for the functional effects of local and larger structural variants, it is important to note the hierarchy of genomic rearrangements, as local variants within rearranged regions show different inheritance patterns to those in syntenic regions. SVs can influence recombination studies as rearrangement hotspots typically have lower synteny and reduced recombination rates [65]. In addition, SVs can influence gene expression directly or indirectly making their functional interpretation harder [58].

Surprisingly, despite the high completeness, we found that the total assembled size of each of the haplogenomes is substantially smaller than that of the *E. grandis* v2.0 reference genome and previous flow cytometry estimates. We propose that the size difference is not due to collapse of the repeat content of the haplogenome assemblies, but rather due to possible overestimation of the *E. grandis* v2.0 genome assembly as a result of inclusion of partially overlapping alternative haplotypes in highly heterozygous regions of the diploid genome assembly. However, resolving this discrepancy will require further de novo genome assemblies for *E. grandis*, possibly including resequencing using long read technology to update the genome assembly of the reference BRASUZ1 individual, as has been performed for some reference genomes that were originally assembled with Sanger sequencing data [72].

Finally, we provide the first genome-wide SV between *E. urophylla* and *E. grandis*. We identify 48,729 SVs, ranging in size from 100 bp to 4.91 Mb. Some of these variants are large enough to be able to cover multiple genes and future studies will focus on understanding the genomic context and functional implications of these variants. An added advantage of this study is the reduced false discovery of SVs which may be introduced when comparing genomes assembled using different pipelines.

Conclusions

We have produced phased, haplogenome assemblies of an interspecific F1 hybrid using a trio-binning approach and performed the first genome-wide analysis of genome synteny between two key tree species used in plantation forestry, *E. grandis* and *E. urophylla*. This revealed a large number of previously undescribed genome structural variants as a step towards understanding genome structural evolution in this iconic genus of fast-growing woody perennials. The haplogenome resource data provides the first insight into haplotype diversity in F1 hybrids and, with additional haplogenomes to be sequenced, this will lead to a better understanding of the genetic basis of hybrid compatibility and superiority. This work is a pilot study towards understanding pan-genome variation in *Eucalyptus* that can be used for tree improvement. The project also produced the first draft, near complete genome assembly for *E. urophylla*, a key tropical eucalypt with an interesting island colonization history.

Additional Files

Supplementary Figure S1. Genome size estimates for the (A) *E. urophylla*, (B) *E. grandis* and (C) the *E. urophylla* x *E. grandis* F1 hybrid genomes.
Supplementary Figure S2. Benchmarking Universal Single-Copy Orthologs (BUSCO) completeness scores for both haplogenome assemblies as well as the currently available *E. grandis* v2.0 reference genome.
Supplementary Figure S3. Alignment of placed haplogenome scaffolds to the *E. grandis* v2.0 reference genome.
Supplementary Figure S4. Alignment between the *E. grandis* and *E. urophylla* scaffolded haplogenome assemblies.
Supplementary Figure S5. Pseudochromosomes of *E. urophylla* haplogenome, reconstructed from two genetic linkage input maps – uro.allmap and gra.allmap, with unequal weights (2 and 1 respectively).
Supplementary Figure S6. Pseudochromosomes of *E. grandis* haplogenome, reconstructed from two genetic linkage input maps – gra.allmap and uro.allmap, with unequal weights (2 and 1 respectively).
Supplementary Figure S7. Scaffolded chromosome sizes of the *E. grandis* v2.0 and the scaffolded *E. grandis* and *E. urophylla* haplogenome assemblies.
Supplementary Figure S9. Syntenic and rearranged regions between the *E. grandis* v2.0, *E. grandis* and *E. urophylla* haplogenomes for all eleven chromosomes.
Supplementary Figure S10. Enriched gene ontology (GO) terms for inverted and translocated gene alignment blocks.
Supplementary Figure S11. Hap-mer blob plot of the *E. grandis* and *E. urophylla* haplogenome assemblies.
Supplementary Figure S12. Evaluation of haplotype phase blocks. All hap-mer information was generated with Merqury v1.1 [68].
Supplementary Note 1. Hap-mer based phasing completeness assessment.
Supplementary Note 2. Read and assembly alignment and validation of high peak content
Supplementary Table S1. Illumina sequencing results.
Supplementary Table S2. Nanopore sequencing results for the F1 hybrid individual.
Supplementary Table S3. Summary statistics for long-read binning using the parental short reads.
Supplementary Table S4. Summary statistics of placed and unplaced contigs after scaffolding with ALLMAPS for the *E. urophylla* and *E. grandis* haplogenomes respectively.
Supplementary Table S5. Repeat element content of assembled haplogenomes.
Supplementary Table S6. Haplogenome annotation statistics.
Supplementary Table S7. Number and total length of syntenic and rearranged regions in the *E. grandis* and *E. urophylla* haplogenomes.
Supplementary Table S8. Number and total length of local sequence variation in syntenic and rearranged region in the *E. grandis* and *E. urophylla* haplogenomes.
Supplementary Table S9. Inversions larger than 50 kb between

the *E. grandis* and *E. urophylla* haplogenomes.
Supplementary Table S10. Translocations between the *E. grandis* and *E. urophylla* haplogenomes that are larger than 50 kb.
Supplementary Table S11. Phase block statistics of the *E. grandis* and *E. urophylla* haplo-genome assemblies.
Supplementary Table S12. *E. grandis* and *E. urophylla* high coverage bin content.

Availability of source code and requirements

All scripts are available at: https://gitlab.com/Anneri/eucalyptus-haplogenome-synteny.

Data Availability

Illumina DNA sequencing data was uploaded at NCBI SRA under BioProject: PRJNA870669. High density genetic linkage maps are available on github. The haplogenome assemblies were uploaded to the NCBI database and can be accessed with accession no. JAOPUP000000000 and JAOPUO000000000. All supporting data such as repeat element libraries, genome annotation files, synteny analyses output files etc. are available upon request.

Declarations

List of abbreviations

BAC: bacterial artificial chromosome, BLAST: Basic Local Alignment Search Tool, BUSCO: Benchmarking Universal Single-Copy Orthologs, BWA: BurrowsWheeler Aligner, CDS: coding sequence, Chr: Chromosome, DUP: duplication, EGR: *E. grandis*, EUR: *E. urophylla*, FDR: false discovery rate, Gb: Gigabase, GO: gene ontology, HDR: highly diverged regions, HMW: high molecular weight, INV: inversion, kb: kilobase, KEGG: Kyoto Encyclopedia of Genes and Genomes, LINE: long interspersed nucleotide element, LRS: Long-read sequencing, LTR: long terminal repeat, Mb: megabase, NCBI: National Center for Biotechnology Information, ONT: Oxford Nanopore Technologies, PacBio: Pacific Biosciences, PE: paired-end, QC: quality control, QUAST: quality assessment tool, QV: quality value, RNA-seq: RNA sequencing, SNP: single nucleotide polymorphism, SRA: Sequence Read Archive, SRS: short read sequencing, SVs: structural variants, SYN: syntenic region, SyRI: Synteny and Rearrangement Identifier, TE: transposable element, TRANS: translocation.

Consent for publication

Not applicable

Competing Interests

The authors declare that they have no competing interests.

Funding

This work was funded by the South African Department of Science and Innovation and Technology Innovation Agency (DSI/TIA, Strategic Grant for Eucalyptus Genomics), the Forestry Sector Innovation Fund (FSIF) and Sappi South Africa through the Forest Molecular Genetics (FMG) Industry Consortium at the University of Pretoria (UP). AL acknowledges MSc bursary support from the National Research Foundation (NRF) of South Africa (Grant Number MND190406427887) and funding from the UP Postgraduate Studies Abroad Programme.

Author's Contributions

AL performed all analyses in the manuscript and prepared the manuscript. JC constructed genetic linkage maps for genome scaffolding. TD and JLW provided bioinformatic and technical support and advised on data analysis and interpretation throughout the project. TD, EM and JLW co-supervised the project. AAM conceived and supervised the project. All authors read and contributed to the final manuscript.

Acknowledgements

The authors thank the UP Bioinformatics and Computational Biology Centre and University of Connecticut (UConn) Computational Biology Core for bioinformatics and computational biology support. Sappi Forest Research (Howick, South Africa) kindly provided the plant materials used in the study.

References

1. Grierson CS, Barnes SR, Chase MW, Clarke M, Grierson D, Edwards KJ, et al. One hundred important questions facing plant science research. New Phytol 2011;192(1):6–12.
2. Grattapaglia D, Kirst M. Eucalyptus applied genomics: from gene sequences to breeding tools. New Phytol 2008;179(4):911–929.
3. de Assis TF. Production and use of Eucalyptus hybrids for industrial purposes. Noosa, Queensland, Australia; 2000.
4. Rezende GDSP, de Resende MDV, de Assis TF. Chapter 16. In: Eucalyptus Breeding for Clonal Forestry Forestry Sciences, Springer; 2014. p. 393–424.
5. Grattapaglia D, Silva-Junior OB, Resende RT, Cappa EP, Muller BSF, Tan B, et al. Quantitative Genetics and Genomics Converge to Accelerate Forest Tree Breeding. Front Plant Sci 2018;9:1693.
6. Zheng GX, Lau BT, Schnall-Levin M, Jarosz M, Bell JM, Hindson CM, et al. Haplotyping germline and cancer genomes with high-throughput linked-read sequencing. Nat Biotechnol 2016;34(3):303–11.
7. Jiao WB, Schneeberger K. The impact of third generation genomic technologies on plant genome assembly. Curr Opin Plant Biol 2017;36:64–70.
8. Alonge M, Wang X, Benoit M, Soyk S, Pereira L, Zhang L, et al. Major Impacts of Widespread Structural Variation on Gene Expression and Crop Improvement in Tomato. Cell 2020;182(1):145–161 e23.
9. Ogawa D, Nonoue Y, Tsunematsu H, Kanno N, Yamamoto T, Yonemaru JI. Discovery of QTL Alleles for Grain Shape in the Japan-MAGIC Rice Population Using Haplotype Information. G3 (Bethesda) 2018;8(11):3559–3565.
10. Ogawa D, Yamamoto E, Ohtani T, Kanno N, Tsunematsu H, Nonoue Y, et al. Haplotype-based allele mining in the Japan-MAGIC rice population. Sci Rep 2018;8(1):4379.
11. Motazedi E, Finkers R, Maliepaard C, de Ridder D. Exploiting next-generation sequencing to solve the haplotyping puzzle in polyploids: a simulation study. Brief Bioinform 2018;19(3):387–403.
12. Bevan MW, Uauy C, Wulff BB, Zhou J, Krasileva K, Clark MD. Genomic innovation for crop improvement. Nature 2017;543(7645):346–354.
13. Kyriakidou M, Tai HH, Anglin NL, Ellis D, Stromvik MV. Current Strategies of Polyploid Plant Genome Sequence Assembly. Front Plant Sci 2018;9:1660.
14. Sherman RM, Salzberg SL. Pan-genomics in the human genome era. Nat Rev Genet 2020;21(4):243–254.
15. Bayer PE, Golicz AA, Scheben A, Batley J, Edwards D. Plant pan-genomes are the new reference. Nat Plants 2020;6(8):914–920.
16. Brondani RPV, Brondani C, Tarchini R, Grattapaglia D. Development, characterization and mapping of microsatellite markers in Eucalyptus grandis and E. urophylla. Theoretical and Applied Genetics 1998;97(5-6):816–827.
17. Marques M, Brondani V, Grattapaglia D, Sederoff R. Conservation and synteny of SSR loci and QTLs for vegetative

propagation in four Eucalyptus species. Theor Appl Genet 2002;105(2-3):474–478.
18. Hudson CJ, Kullan ARK, Freeman JS, Faria DA, Grattapaglia D, Kilian A, et al. High synteny and colinearity among Eucalyptus genomes revealed by high-density comparative genetic mapping. Tree Genetics & Genomes 2011;8(2):339–352.
19. Bartholome J, Mandrou E, Mabiala A, Jenkins J, Nabihoudine I, Klopp C, et al. High-resolution genetic maps of Eucalyptus improve Eucalyptus grandis genome assembly. New Phytol 2015;206(4):1283–96.
20. Myburg AA, Grattapaglia D, Tuskan GA, Hellsten U, Hayes RD, Grimwood J, et al. The genome of Eucalyptus grandis. Nature 2014;510(7505):356–62.
21. Ho SS, Urban AE, Mills RE. Structural variation in the sequencing era. Nat Rev Genet 2020;21(3):171–189.
22. Koren S, Walenz BP, Berlin K, Miller JR, Bergman NH, Phillippy AM. Canu: scalable and accurate long-read assembly via adaptive k-mer weighting and repeat separation. Genome Res 2017;27(5):722–736.
23. Shirasawa K, Esumi T, Hirakawa H, Tanaka H, Itai A, Ghelfi A, et al. Phased genome sequence of an interspecific hybrid flowering cherry, 'Somei-Yoshino' (Cerasus x yedoensis). DNA Res 2019;26(5):379–389.
24. Zhu T, Wang L, You FM, Rodriguez JC, Deal KR, Chen L, et al. Sequencing a Juglans regia x J. microcarpa hybrid yields high-quality genome assemblies of parental species. Hortic Res 2019;6:55.
25. Marcais G, Kingsford C. A fast, lock-free approach for efficient parallel counting of occurrences of k-mers. Bioinformatics 2011;27(6):764–70.
26. Ranallo-Benavidez TR, Jaron KS, Schatz MC. GenomeScope 2.0 and Smudgeplot for reference-free profiling of polyploid genomes. Nat Commun 2020;11(1):1432.
27. Kim D, Song L, Breitwieser FP, Salzberg SL. Centrifuge: rapid and sensitive classification of metagenomic sequences. Genome Res 2016;26(12):1721–1729.
28. Wood DE, Lu J, Langmead B. Improved metagenomic analysis with Kraken 2. Genome Biol 2019;20(1):257.
29. Zimin AV, Puiu D, Luo MC, Zhu T, Koren S, Marcais G, et al. Hybrid assembly of the large and highly repetitive genome of Aegilops tauschii, a progenitor of bread wheat, with the MaSuRCA mega-reads algorithm. Genome Res 2017;27(5):787–792.
30. Gurevich A, Saveliev V, Vyahhi N, Tesler G. QUAST: quality assessment tool for genome assemblies. Bioinformatics 2013;29(8):1072–5.
31. Mikheenko A, Prjibelski A, Saveliev V, Antipov D, Gurevich A. Versatile genome assembly evaluation with QUAST-LG. Bioinformatics 2018;34(13):i142–i150.
32. Simao FA, Waterhouse RM, Ioannidis P, Kriventseva EV, Zdobnov EM. BUSCO: assessing genome assembly and annotation completeness with single-copy orthologs. Bioinformatics 2015;31(19):3210–2.
33. Seppey M, Manni M, Zdobnov EM. BUSCO: Assessing Genome Assembly and Annotation Completeness. Methods Mol Biol 2019;1962:227–245.
34. Manni M, Berkeley MR, Seppey M, Simao FA, Zdobnov EM. BUSCO Update: Novel and Streamlined Workflows along with Broader and Deeper Phylogenetic Coverage for Scoring of Eukaryotic, Prokaryotic, and Viral Genomes. Mol Biol Evol 2021;38(10):4647–4654. https://www.ncbi.nlm.nih.gov/pubmed/34320186.
35. Li H, Durbin R. Fast and accurate short read alignment with Burrows-Wheeler transform. Bioinformatics 2009;25(14):1754–60.
36. Li H, Handsaker B, Wysoker A, Fennell T, Ruan J, Homer N, et al. The Sequence Alignment/Map format and SAMtools. Bioinformatics 2009;25(16):2078–9.
37. Tang H, Zhang X, Miao C, Zhang J, Ming R, Schnable JC, et al. ALLMAPS: robust scaffold ordering based on multiple maps. Genome Biol 2015;16:3.
38. Li H. Minimap and miniasm: fast mapping and de novo assembly for noisy long sequences. Bioinformatics 2016;32(14):2103–10.
39. Cabanettes F, Klopp C. D-GENIES: dot plot large genomes in an interactive, efficient and simple way. PeerJ 2018;6:e4958.
40. Smit A, Hubley R. RepeatModeler Open-1.0.; 2008, http://www.repeatmasker.org/RepeatModeler/.
41. Smit AFA, Hubley R, Green P. RepeatMasker Open-4.0.; 2013, http://www.repeatmasker.org.
42. Ou S, Jiang N. LTR_retriever: A Highly Accurate and Sensitive Program for Identification of Long Terminal Repeat Retrotransposons. Plant Physiol 2018;176(2):1410–1422.
43. Krzywinski M, Schein J, Birol I, Connors J, Gascoyne R, Horsman D, et al. Circos: an information aesthetic for comparative genomics. Genome Res 2009;19(9):1639–45.
44. Mizrachi E, Hefer CA, Ranik M, Joubert F, Myburg AA. De novo assembled expressed gene catalog of a fast-growing Eucalyptus tree produced by Illumina mRNA-Seq. BMC Genomics 2010;11:681.
45. Vining KJ, Romanel E, Jones RC, Klocko A, Alves-Ferreira M, Hefer CA, et al. The floral transcriptome of Eucalyptus grandis. New Phytol 2015;206(4):1406–22.
46. Mizrachi E, Verbeke L, Christie N, Fierro AC, Mansfield SD, Davis MF, et al. Network-based integration of systems genetics data reveals pathways associated with lignocellulosic biomass accumulation and processing. Proc Natl Acad Sci U S A 2017;114(5):1195–1200.
47. Bolger AM, Lohse M, Usadel B. Trimmomatic: a flexible trimmer for Illumina sequence data. Bioinformatics 2014;30(15):2114–20.
48. Kim D, Paggi JM, Park C, Bennett C, Salzberg SL. Graph-based genome alignment and genotyping with HISAT2 and HISAT-genotype. Nat Biotechnol 2019;37(8):907–915.
49. Gremme G, Brendel V, Sparks ME, Kurtz S. Engineering a software tool for gene structure prediction in higher organisms. Information and Software Technology 2005;47(15):965–978.
50. Hoff KJ, Lange S, Lomsadze A, Borodovsky M, Stanke M. BRAKER1: Unsupervised RNA-Seq-Based Genome Annotation with GeneMark-ET and AUGUSTUS. Bioinformatics 2016;32(5):767–769.
51. Lomsadze A, Burns PD, Borodovsky M. Integration of mapped RNA-Seq reads into automatic training of eukaryotic gene finding algorithm. Nucleic Acids Res 2014;42(15):e119.
52. Stanke M, Diekhans M, Baertsch R, Haussler D. Using native and syntenically mapped cDNA alignments to improve de novo gene finding. Bioinformatics 2008;24(5):637–44.
53. Stanke M, Keller O, Gunduz I, Hayes A, Waack S, Morgenstern B. AUGUSTUS: ab initio prediction of alternative transcripts. Nucleic Acids Res 2006;34(Web Server issue):W435–9.
54. Camacho C, Coulouris G, Avagyan V, Ma N, Papadopoulos J, Bealer K, et al. BLAST+: architecture and applications. BMC Bioinformatics 2009;10:421.
55. Caballero M, Wegrzyn J. gFACs: Gene Filtering, Analysis, and Conversion to Unify Genome Annotations Across Alignment and Gene Prediction Frameworks. Genomics Proteomics Bioinformatics 2019;17(3):305–310.
56. Hart AJ, Ginzburg S, Xu MS, Fisher CR, Rahmatpour N, Mitton JB, et al. EnTAP: Bringing faster and smarter functional annotation to non-model eukaryotic transcriptomes. Mol Ecol Resour 2020;20(2):591–604.

57. Kurtz S, Phillippy A, Delcher AL, Smoot M, Shumway M, Antonescu C, et al. Versatile and open software for comparing large genomes. Genome Biol 2004;5(2):R12.
58. Goel M, Sun H, Jiao WB, Schneeberger K. SyRI: finding genomic rearrangements and local sequence differences from whole-genome assemblies. Genome Biol 2019;20(1):277.
59. Tang H, Bowers JE, Wang X, Ming R, Alam M, Paterson AH. Synteny and collinearity in plant genomes. Science 2008;320(5875):486–8.
60. Conesa A, Gotz S, Garcia-Gomez JM, Terol J, Talon M, Robles M. Blast2GO: a universal tool for annotation, visualization and analysis in functional genomics research. Bioinformatics 2005;21(18):3674–6.
61. Grattapaglia D, Bradshaw Jr HD. Nuclear DNA content of commercially important Eucalyptus species and hybrids. Canadian Journal of Forest Research 1994;24(5):1074–1078.
62. Marks RA, Hotaling S, Frandsen PB, VanBuren R. Representation and participation across 20 years of plant genome sequencing. Nat Plants 2021;7(12):1571–1578.
63. Kullan ARK, van Dyk MM, Jones N, Kanzler A, Bayley A, Myburg AA. High-density genetic linkage maps with over 2,400 sequence-anchored DArT markers for genetic dissection in an F2 pseudo-backcross of Eucalyptus grandis × E. urophylla. Tree Genetics & Genomes 2011;8(1):163–175.
64. Zhou Y, Minio A, Massonnet M, Solares E, Lv Y, Beridze T, et al. The population genetics of structural variants in grapevine domestication. Nat Plants 2019;5(9):965–979.
65. Jiao WB, Schneeberger K. Chromosome-level assemblies of multiple Arabidopsis genomes reveal hotspots of rearrangements with altered evolutionary dynamics. Nat Commun 2020;11(1):989.
66. Koren S, Rhie A, Walenz BP, Dilthey AT, Bickhart DM, Kingan SB, et al. De novo assembly of haplotype-resolved genomes with trio binning. Nat Biotechnol 2018;.
67. Wang W, Das A, Kainer D, Schalamun M, Morales-Suarez A, Schwessinger B, et al. The draft nuclear genome assembly of Eucalyptus pauciflora: a pipeline for comparing de novo assemblies. Gigascience 2020;9(1).
68. Rhie A, Walenz BP, Koren S, Phillippy AM. Merqury: reference-free quality, completeness, and phasing assessment for genome assemblies. Genome Biol 2020;21(1):245.
69. Moran GF, Bell JC, Griffin AR. Reduction in levels of inbreeding in a seed orchard of Eucalyptus regnans F. Muall. compared with natural populations. Silvae Genetica 1989;38(1).
70. Gaiotto FA, Bramucci M, Grattapaglia D. Estimation of outcrossing rate in a breeding population of Eucalyptus urophylla with dominant RAPD and AFLP markers. Theoretical and Applied Genetics 1997;95(5-6):842–849.
71. Wang X, Gao L, Jiao C, Stravoravdis S, Hosmani PS, Saha S, et al. Genome of Solanum pimpinellifolium provides insights into structural variants during tomato breeding. Nat Commun 2020;11(1):5817.
72. Nurk S, Koren S, Rhie A, Rautiainen M, Bzikadze AV, Mikheenko A, et al. The complete sequence of a human genome. Science 2022;376(6588):44–53.

Figure legends

Figure 1. Separation of *E. urophylla* and *E. grandis* haplogenomes in the F1 hybrid using a trio-binning strategy. Using whole-genome Illumina short-read sequencing data of the parental genomes, long-read sequencing data of the F1 hybrid offspring is separated based on unique parental k-mers into *E. urophylla* and *E. grandis* haplotype bins (amount of Nanopore sequencing data is indicated in gigabases (Gb) below each bin, as well as the estimated genome coverage). Reads that contain no unique k-mers were unbinned and kept in their own bin. Long reads were subsequently assembled independently, resulting in fully assembled *E. urophylla* and *E. grandis* haplogenome (total assembly size is shown below the relevant haplogenome and size of assembly scaffolded into eleven chromosomes are indicated in brackets). This figure is adapted from [66] and tree images are from `https://rooweb.com.au/`.

Figure 2. Synteny and distribution of LTR retrotransposons along the *E. grandis* and *E. urophylla* haplogenome assemblies for eleven scaffolded chromosomes. Syntenic regions are shown between the *E. urophylla* and *E. grandis* haplogenomes in the middle, based on SyRI (see Supplementary Figure S9). LTR retrotransposon distribution is shown for the *E. urophylla* (EUR) and the *E. grandis* (EGR) haplogenome assemblies. From outside to inside, the heatmaps show the distribution of Copia (orange, ranging from 6 to 21.5%), Gypsy (blue, ranging from 1.3 to 26.5%) and unknown (green, ranging from 2.8 to 16.6%) LTR retrotransposons, GC% (37.0 to 43.0%) and gene density (0 to 60.0%) with darker shades representing a higher percentage of retrotransposons within the bin. Chromosome number and size is indicated on the outer circle in megabases.

Figure 3. Size and distribution of structural rearrangements and local variants between the *E. grandis* and *E. urophylla* haplogenomes. (A) Distribution of syntenic regions and structural variants between the *E. grandis* and *E. urophylla* haplogenome assemblies. Links are shown between *E. urophylla* (EUR) chromosomes in blue and and *E. grandis* (EGR) chromosomes in green. Only variants of greater than 10 kilobases as identified by SyRI are shown. Darker links in show change in chromosome number between EUR and EGR. (B) Total size of syntenic and rearranged regions in megabases (Mb) for the *E. grandis* and *E. urophylla* haplogenome (see Supplementary Table S7 and Supplementary Table S8). The size of syntenic or rearranged regions are indicated within the bar in Mb, while the bar colour represents the rearrangement type. (C) Size distribution of rearranged regions (left) and local variants (right) between the *E. grandis* and *E. urophylla* haplogenomes. Size is indicated in base pairs on the y-axis (ranging from one to 4.91 Mb for rearrangements and one to 3.09 Mb for local variants), and the rearrangement type on the x-axis; INV are inversions, DUP are duplications, TRANS are translocations, NOTAL are regions that are not aligned, TDM are tandem repeats, CPG and CPL are copy gains/losses, HDR are highly diverged regions, INS are insertions and DEL are deletions.

Figure 4. Gene synteny between *E. grandis* and *E. urophylla* haplogenome assemblies. (A) Chromosome-scale collinearity between *E. grandis* and *E. urophylla* haplogenome annotations. Lines in light grey indicate syntenic gene blocks, lines in purple indicate inverted gene blocks, blue indicated translocated gene block and dark grey inverted translocated gene blocks. Only block that span greater than 30 gene pairs are shown. (B) Dot-plot alignments of 23,390 gene pairs between the *E. grandis* and *E. urophylla* haplogenome annotations. (C) Bar graph showing syntenic depth of *E. grandis* and *E. urophylla* syntenic blocks. The majority of genes are in a 1 to 1 synteny pattern. All of the graphs were produced in MCScan JCVI v1.1.18 [59].

Manuscript

Click here to access/download;Manuscript;Haplogenome_assembly_of_Euc

GigaScience, 2022, 1–11

doi: xx.xxxx/xxxx
Manuscript in Preparation
Paper

PAPER

Haplogenome assembly reveals interspecific structural variation in *Eucalyptus* hybrids

Anneri Lötter[1], Tuan A. Duong[1], Julia Candotti[1], Eshchar Mizrachi[1], Jill L. Wegrzyn[2] and Alexander A. Myburg[1,*]

[1]Department of Biochemistry, Genetics and Microbiology, Forestry and Agricultural Biotechnology Institute (FABI), University of Pretoria, Private bag X20, Pretoria 0028, South Africa and [2]Department of Ecology and Evolutionary Biology, Institute for Systems Genomics: Computational Biology Core, University of Connecticut, 67 N. Eagleville Road, Storrs, Connecticut, USA

[*]**corresponding author:** zander.myburg@fabi.up.ac.za

Abstract

Background: *De novo* phased (haplo)genome assembly using long-read DNA sequencing data has improved the detection and characterization of structural variants (SVs) in plant and animal genomes. Able to span across haplotypes, long reads allow phased, haplogenome assembly in highly outbred organisms such as forest trees. *Eucalyptus* tree species and interspecific hybrids are the most widely planted hardwood trees with F1 hybrids of *Eucalyptus grandis* and *E. urophylla* forming the bulk of fast-growing pulpwood plantations in subtropical regions. The extent of structural variation and its effect on interspecific hybridization is unknown in these trees. As a first step towards elucidating the extent of structural variation between the genomes of *E. grandis* and *E. urophylla*, we sequenced and assembled the haplogenomes contained in an F1 hybrid of the two species. **Findings:** Using Nanopore sequencing and a trio-binning approach, we assembled the separate haplogenomes (567 Mb and 545 Mb) to 97.9% BUSCO completion. High-density SNP genetic linkage maps of both parents allowed scaffolding of 88% of the haplogenome contigs into 11 pseudo-chromosomes (scaffold N50 of 43.82 Mb and 42.45 Mb for the *E. grandis* and *E. urophylla* haplogenomes, respectively). We identify 48,729 SVs between the two haplogenomes providing the first detailed insight into genome structural rearrangement in these species. The two haplogenomes have similar gene content, 35,572 and 33,915 functionally annotated genes, of which 34% are contained in genome rearrangements. **Conclusions:** Knowledge of SV and haplotype diversity in the two species will form the basis for understanding the genetic basis of hybrid superiority in these trees.

Key words: *Eucalyptus*; trio-binning; phased genome assembly; Nanopore; structural variant

Background

There is considerable pressure to improve crop yields to provide food, fibre, shelter and renewable energy for the growing human population [1] in a sustainable manner. Fast-growing *Eucalyptus* tree species provide an important renewable feedstock for biomaterial (timber, fibre and lignocellulosics) and bioenergy production, relieving pressure on native forests [2]. These species, commonly referred to as eucalypts, constitute the most widely planted hardwood fibre crop globally. The most productive plantation areas are planted with interspecific F1 hybrid clones that combine favourable characteristics of parental species and generally lead to increased forest productivity and product quality, and reduced production costs [2, 3]. The most widely planted hybrid combination in subtropical regions, *E. grandis* x *E. urophylla*, is primarily bred to combine the disease resistance of the tropical species *E. urophylla* with the fast growth of the subtropical species *E. grandis*. To further improve plantation productivity, wood quality and resilience, more efficient breeding strategies have been pursued in the past decade, primarily through genomic selection using genome-wide SNP markers [4, 5].

Discriminating the maternal and paternal chromosome copies (defined by haplotypes or blocks of allelic variants that are inherited together; [6]) allows for identification of haplotype and structural variants that may be associated with crop productivity and resilience [7, 8]. Haplotype-based molecular breeding has been shown to be a more accurate and effective breeding strategy [9, 10] compared to SNP based strategies. Haplotypes can often be inferred

Compiled on: October 25, 2022.
Draft manuscript prepared by the author.

accurately in offspring by using the parental genomes and previously defined SNP tag-markers and haplotype imputation with statistical methods [11]. SNP tag-markers can then be used in accelerated breeding strategies by aiding the selection of progeny for propagation and deployment, or identification of parents for further breeding [12].

Access to multiple high-quality reference genome assemblies facilitates the identification of haplotypes and structural variants, both of which underlie pan-genome variation in plants. Genome assembly in highly outbred organisms such as forest trees, is often hampered by high levels of heterozygosity and the frequent occurrence of non-syntenic DNA sequences in intergenic regions leading to mixed phase contigs. As a consequence, many of the available reference sequences of outbred plants do not accurately reflect the haplogenomes carried by the reference individuals [13]. Long-read sequencing (LRS) technologies such as Oxford Nanopore (ONT) and Pacific Biosciences (PacBio) can mitigate the challenges associated with assembling outbred plant genomes. Long reads can span across multiple syntenic (gene) regions and connect intergenic allelic variants between them, allowing separate, phased assembly of haplotype and structural variant alternatives. The growing number of phased genome assemblies, especially those assembled with LRS data, has revealed that a single flat reference genome misses a substantial portion of the genotypic diversity in outbred species [14]. As such, there is a movement towards assembly of a pan-reference genome, which incorporates variants from multiple individuals as has been reported in humans (reviews by [14]) and plants (reviewed by [15]).

Studies on pan-genomic (including haplotype and structural) variation are still lacking in *Eucalyptus*, with most information on genome synteny still derived from genetic linkage mapping. These studies have suggested that there is high collinearity between eucalypt species, including *E. grandis* and *E. urophylla* [16, 17, 18, 19] . However, the degree of fine scale synteny between *E. grandis* and *E. urophylla* is unknown as there is not a *de novo* reference assembly available for *E. urophylla*, one of the most important hybrid parent partners. The current reference genome, *E. grandis* [20], was sequenced with a combination of Sanger and SRS. These technologies have limited capability to identify haplotype and structural variants (reviewed by [21]). The lack of available LRS based genome assemblies for *E. grandis* and *E. urophylla* have precluded studies of pan-genome variation in these species and their F1 hybrids.

Combining SRS and LRS data with a parent-offspring trio-sequencing approach has been demonstrated to allow assembly of high-quality haplo-reference genomes for the two parents, at a lower cost than generating two independent reference quality genomes[22, 23, 24]. Similarly, trio-sequencing of an interspecific F1 hybrid of *E. grandis* and *E. urophylla*, paired with LRS technologies will generate high-quality assemblies of the haplogenomes contained in the F1 hybrid. These high-quality phased genome assemblies will ultimately provide a basis for pursuing haplotype-based molecular breeding of eucalypt trees and will provide preliminary insights into the abundance and distribution of structural variants (SVs) of consequence to breeding. Thus, the aim of this study was to create a starting point for defining pan-genome, haplotype and structural variation in *E. grandis* and *E. urophylla*.

Methods

Sample background

Leaf tissues of an F1 *E. urophylla* x *E. grandis* hybrid offspring and its parents (*E. urophylla* seed parent and *E. grandis* pollen parent) were collected and used for DNA extractions. These individuals form part of a large nested association mapping trial and SNP data was used to generate high-density genetic linkage maps for both the *E. grandis* and *E. urophylla* parents (Candotti *et al.*). Sequencing both parents will enable a) inference of both haplotypes for the parental genomes and b) haplotype binning for genome phasing (Figure 1).

DNA Isolation

Illumina sequencing

Genomic DNA was extracted from 50 mg of leaf tissue for the *E. urophylla* and *E. grandis* parents using the NucleoSpin® Plant II Kit (Machery-Nagel, Germany). Gel electrophoresis was performed using a 0.8% w/v agarose gel to assess DNA quality. DNA quality was also assessed using a NanoDrop® ND-1000 spectrophotometer (Thermo Fisher Scientific) and quantified using a Qubit 2.0 Fluorometer (Thermo Fisher Scientific). Whole-genome sequencing of the F1 hybrid and its parents was performed on an Illumina NovaSeq6000 platform (Illumina NovaSeq 6000 Sequencing System, RRID:SCR_016387) by Macrogen (Macrogen Inc., Seoul, Korea).

High molecular weight DNA extraction

Genomic DNA was extracted using 1.2 g of flash frozen ground leaf tissue. The ground material was suspended in 25 ml Guanidine buffer (20 mM EDTA, 100 mM NaCl, 1% Trition® X-100, 500 mM Guanidine-HCl and 10 mM Tris, pH 7.9), supplemented with 50 mg cellulase (Sigma-Aldrich) and 50 mg lysing enzyme (Sigma-Aldrich) incubated at 42 °C with gentle agitation. After 2.5 h, 10 µl RNase A (20 µg/ml) was added and the sample was incubated for 30 min at 37 °C, after which 50 mg proteinase K was added, and the mixture was incubated for another 2 h at 50 °C. The mixture was then centrifuged for 20 min at 12 000 x g and the clarified lysate transferred to an appropriate buffer QBT-equilibrated QIAGEN Genomic-tip 100/G column (Qiagen), after which the column was washed three times with 7 ml Buffer QC and HMW DNA was eluted with 5 ml Buffer QF. The DNA was precipitated by adding 0,7 V of isopropanol and centrifuged at 12 000 g for 20 min. The DNA pellet was washed twice with 70% Ethanol and resuspended in an appropriate volume of low salt TE (10 mM Tris-HCL pH 8.0; 0.1 mM of EDTA). Gel electrophoresis was performed using a 0.8% w/v agarose gel to assess DNA quality, and DNA quantity was assessed using a Qubit 2.0 Fluorometer (Thermo Fisher Scientific).

Nanopore sequencing

HMW DNA was prepared for MinION (RRID:SCR_017985) sequencing following the manufacturers protocol using the genomic sequencing kit SQK-LSK109 (Oxford Nanopore Technologies, Oxford, UK). Approximately 3.3 µg of HMW DNA from was used without exogenous shearing or size selection. HMW DNA was first repaired with NEBNext FFPE Repair Mix (New England Biolabs) and 3'-adenylated with NEBNext Ultra II End Repair/dA-Tailing Module (NEB). The DNA was then purified with AMPure XP beads (Beckmann Coulter) and ligated with sequencing adapters (ONT) using NEBNext Quick T4 DNA Ligase (NEB). After purification with AMPure XP beads (Beckman Coulter), the library was mixed with sequencing buffer (ONT) and library loading beads (ONT) and loaded on primed MinION R9.4 SpotOn flow cells (FLO-MIN106). MinION sequencing was performed with a MinION Mk1B sequencer running for 48 h.

The resulting FAST5 files were base-called and reads with a QV < 7 were removed with Oxford Nanopore Technologies' Guppy basecalling software v3.4.5 (ONT) using parameters for FLO-MIN106 and SQK-LSK109 library type. RStudio was used summarise and visualise statistics for both DNA isolation methods based on the sequencing summary file generated by the Guppy base-caller. The Guppy base-caller may not remove all the sequence adapters so to ensure all sequence adapters are removed Porechop v0.2.4 (Porechop, RRID:SCR_016967) was used. All scripts used in this study is available on github. The resulting adapter-less reads of both DNA isolation methods were combined into a single FASTQ file for further use.

PromethION (RRID:SCR_017987) sequencing was performed by the Centre for Genome Innovation (University of Connecticut,

Connecticut, USA) on a FLO-PRO002 PromethION flow cell as per the PromethION sequencing protocol (ONT) using the SQK-LSK109 (ONT) sequencing kit with Circulomics Short Read Eliminator XS (Circulomics Inc.) size-selection. The flow cell was washed and reloaded after 38 h and run for an additional 6 h of sequencing. Base-calling was performed using the Guppy v3.4.5 basecaller and adapter removal was performed as stated above.

Genome assembly

Trio-binning and haplogenome assembly

Illumina short-reads were used for k-mer based genome size estimation was performed using Jellyfish v2.2.6 (Jellyfish, RRID:SCR_005491) [25] for 21-mers and visualised with GenomeScope v2.0 (GenomeScope, RRID:SCR_017014) [26]. Long-reads of the F1 hybrid were binned into *E. urophylla* and *E. grandis* haplotype bins (corresponding to parental short-reads) using the Trio-Canu module in Canu v1.8 (Canu, RRID:SCR_015880) [22]. Read contaminants were identified from the binned reads using Centrifuge v1.0.4-beta (Centrifuge Classifier, RRID:SCR_016665) [27] and removed with a custom script. Similarly, contaminant reads were also identified and removed from short read data with Kraken v2.0.8-beta (Kraken, RRID:SCR_005484) [28]. The remaining raw reads were used for all assembly and alignment steps.

The binned reads corresponding to each of the parents were assembled separately, along with the corresponding parental short reads, using the MaSuRCA v3.3.4 (MaSuRCA, RRID:SCR_010691) [29] genome assembler. MaSuRCa was chosen as initial testing of multiple genome assemblers (based on the BUSCO completion score, contig N50 and total assembly size) indicated that the MaSuRCA genome assembler performed the best. Quality of the resulting assemblies was assessed using QUAST v5.0.2 (QUAST,RRID:SCR_001228) [30, 31] and BUSCO v5.0.0 (BUSCO, RRID:SCR_015008) [32, 33, 34]. To verify genome coverage of the assemblies, Illumina reads from each of the parental haplotypes were mapped to the corresponding and alternative assembled haplogenomes using BWA v0.7.5a-r405 (BWA, RRID:SCR_010910) [35] and mapping rate calculated using the flagstat module from Samtools v1.9 (SAMTOOLS, RRID:SCR_002105) [36].

Genome scaffolding

To improve assembly contiguity, scaffolding was performed for the MaSuRCa assembled *E. urophylla* and *E. grandis* genomes using high-density genetic linkage maps previously constructed for each of the parents (Candotti *et al.*). To resolve possible chimeric contigs that were assembled by MaSuRCa, Polar_Star (`https://github.com/phasegenomics/polar_star`) was used to infer breakpoints and split in contigs based on identification of read-depth outliers from the binned long-reads. After breakpoints were inferred and contigs split, all contigs smaller than 3 kb were removed before scaffolding with high-density genetic linkage maps. A BLAST database was created for both assembled haplogenomes to identify the position of 1,588 *E. grandis* and 1,575 *E. urophylla* SNP probes used to construct the genetic maps. A consensus map was constructed with ALLMAPS (ALLMAPS, RRID:SCR_021171) [37], consisting of SNPs that mapped to the assembled haplogenomes, and was used to perform genome scaffolding. For the consensus map construction, a weight of two was given to the parental genetic linkage map corresponding to species haplogenome to be scaffolded, while a weight of one was given for the alternative parental linkage map. Chromosome scaffold sizes from the two haplogenomes were compared to one another and to the *E. grandis* v2.0 genome to see whether there is a size difference between the *E. grandis* v2.0 reference potential bias in scaffolding of particular chromosomes. To validate if unplaced contigs/scaffolds were from a particular chromosome, unplaced contigs/scaffolds were aligned to the *E. grandis* v2.0 genome using MiniMap2 (RRID:SCR_018550) [38] and alignments visualized with D-Genies (RRID:SCR_018967) [39].

Genome annotation

Custom libraries of repetitive elements were constructed for the *E. urophylla* and *E. grandis* haplogenomes with RepeatModeler v1.0.8 (RepeatModeler, RRID:SCR_015027) [40]. Repetitive elements were annotated with RepeatMasker v4.0.9 (RepeatMasker, RRID:SCR_012954) [41] for the haplogenome assemblies. To eliminate the chance of missing repeat elements in either haplogenome due to them not being identified, the combined species library was used as input for RepeatMasker. Lastly, to identify the abundance of LTR retrotransposons, LTR retrotransposon candidates were identified with LTR_retriever (RRID:SCR_017623) [42] for both haplogenomes and their distribution visualised with Circos (RRID:SCR_011798) [43].

RNA-Seq reads from previous studies were used for structural genome annotation. RNA-Seq reads used for the *E. grandis* haplogenome assembly were from the original genome assembly paper and included six different tissues from an *E. grandis* individual [44, 45] all data is available on `https://eucgenie.org/`. RNA-Seq data from three-year-old *E. grandis* x *E. urophylla* backcrossed with *E. urophylla* trees were used and only derived from two tissues (mature leaf and xylem, Bioproject: `https://www.ncbi.nlm.nih.gov/bioproject/PRJNA354497`) [46]. RNA-Seq reads were trimmed with Trimmomatic v0.39 (Trimmomatic, RRID:SCR_011848) [47] and only paired reads were used for further work. Trimmed RNA-Seq reads were aligned to the relevant haplogenome assemblies with Hisat2 v2.1.0 (HISAT2, RRID:SCR_015530) [48]. GenomeThreader v1.7.1 [49] was used to align protein sequences of the *E. grandis* v2.0 genome annotation to the haplogenome assemblies. We used BRAKER2 v2.0.5 (BRAKER, RRID:SCR_018964) [50] for structural gene prediction. To predict protein coding regions in the genome, Braker2 first converts RNA-Seq alignments to exon support with GeneMark-ET v4.38 (GeneMark, RRID:SCR_011930) [51]. This output is combined with protein alignments for two rounds of training with AUGUSTUS v3.2.3 (Augustus, RRID:SCR_008417) [52, 53, 54]. The predicted gene space was then filtered with gFACs v1.1.3 (gFACs,) [55]. Mono-exonic genes were filtered with InterProScan v5.35-74.0 (InterProScan, RRID:SCR_005829) to keep only those with known protein domains. Completeness of the structural annotations were assessed with BUSCO v5.0.0.

Functional genome annotation was performed with EnTAP v0.9.0 [56] using the following public databases: NCBI RefSeq complete and EMBL-EBI UniProt. This pipeline integrates similarity search and other annotation resources which include gene family (eggNOG), protein domains (Pfam), gene ontology and KEGG pathway assignment.

Structural variant identification

To check for regions that are unassembled in the haplogenome assemblies compared to the *E. grandis* v2.0 reference genome, the *E. grandis* and *E. urophylla* haplogenomes were each aligned to the *E. grandis* v2.0 genome, with MiniMap2 [38] and alignments visualised using D-Genies [39]. Using the same method, the eleven assembled *E. grandis* and *E. urophylla* chromosomes were aligned to each other to visually identify genomic regions with possible large structural variants (SVs). To identify structural rearrangements (inversions, translocations and duplications) and local variations (SNPs, InDels, copy gains/losses, highly diverged regions and tandem repeats) between *E. grandis* and *E. urophylla*, haplogenome assemblies were aligned to each other using nucmer from the MUMmer3 toolbox (MUMmer, RRID:SCR_018171) [57] with alignment parameters "—maxmatch –c 100 -b 500 -l 50" . The resulting alignments were further filtered for alignment length (>100) and identity (>90). Identification of structural rearrangements and local variations was performed using the Synteny and Rearrangement Identifier (SyRI) pipeline [58]. The same method was also used to

identify regions that differed between the *E. grandis* haplogenome and the *E. grandis* v2.0 reference genome. As the linear visualisation of syntenic regions and variants from SyRI prohibits us from depicting inter-chromosomal events, synteny and variants of greater than 10 kb were visualised with Circos.

Syntenic gene pairs between the *E. grandis* and *E. urophylla* haplogenomes were identified using a python version of MCScan, JCVI v1.1.18 (jcvi, RRID:SCR_021641) [59]. Coding sequence and annotation gff3 files were used as input data to identify the syntenic blocks for each pair of species with the 'jcvi.compara.catalog ortholog' command and a c-score parameter of –cscore=0.95. Syntenic blocks were filtered with 'jcvi.compara.synteny screen' with parameters –minspan=30 –simple. The pattern of synteny was detected with jcvi.compara.synteny depth –histogram. Smaller syntenic blocks were also filtered with 'jcvi.compara.synteny screen' with parameters –minspan=10 –simple. Genes within inverted and translocated syntenic blocks that spanned ten or more gene pairs were checked for gene ontology enrichment terms using Blast2GO v1.20.14 (Blast2GO, RRID:SCR_005828) [60] and results were visualized using Tableau Professional Edition (Tableau Desktop, RRID:SCR_013994) (Tableau Software Inc., Seattle, WA, USA).

Results

Genome sequencing

Illumina sequencing of an F1 hybrid individual (SAP_F1_FK118) and its pure-species *E. grandis* (SAP_GRA_FK1758) and *E. urophylla* (SAP_URO_FK1756) parents (Sappi Forest Research, South Africa) resulted in more than 116 Gb of PE150 data per individual (Supplementary Table S1). Using GenomeScope2.0, we estimated the genome size to be 443.19 Mb, 482.27 Mb and 477.76 Mb for the *E. urophylla*, *E. grandis* parents and the F1 hybrid respectively (Supplementary Figure S1). These short read-based estimates were substantially smaller than previous estimates based on flow cytometry [61] and that reported for the *E. grandis* reference genome [20]. [62] recently reported a lower flow cytometry size estimate (497.7 Mb) for *E. grandis* supporting our findings. Levels of heterozygosity in the short-read data were 2.14%, 2.63% and 3.46% for the *E. grandis*, *E. urophylla* and the F1 hybrid (Supplementary Figure S1) providing ample genetic diversity for trio-binning of the long-reads (see below).

A total of 75.32 Gb of Nanopore sequencing data was generated (read N50 27 kb), of which 68.15 Gb (90.48%) passed QC (Q-value > 7, Supplementary Table S2) and was used for trio-binning corresponding to 105X coverage of the F1 hybrid genome and 50X coverage per haplogenome (Figure 1, Supplementary Table S2).

Genome assembly

Phased hybrid genome assembly using trio-binning

To separately assemble the long reads originating from the two haplogenomes in the F1 hybrid, we performed trio-binning using the Illumina short-read data for the parents and the long-read data for the F1 individual. We were able to bin 1,876,816 reads (32.66 Gb) for the *E. urophylla* haplogenome and 1,998,860 reads (35.11 Gb) for the *E. grandis* haplogenome corresponding to 50X and 54X coverage of the two haplotypes, respectively (Figure 1, Supplementary Table S3). Only 6,693 reads (0.014%) could not be binned and were excluded from further analyses.

Assembly of the binned reads for the *E. urophylla* haplogenome resulted in 654 contigs and a total size of 546.1 Mb, with a contig N50 of 4.41 Mb 1. A BUSCO completeness score of 97.9% was obtained of which 95.2% were single-copy genes and only 2.7% were duplicate-copy genes (Supplementary Figure S2). The reads binned for the *E. grandis* haplogenome assembled into 793 contigs with a total size of 568.5 Mb and a contig N50 of 3.91 Mb 1. For this assembly we obtained a BUSCO completeness score of 98.3%, of which 94.8% were single copy genes and 3.5% were duplicate genes (Supplementary Figure S2). The low duplicate percentages reflected efficient trio-binning and haplogenome assembly.

Next, we mapped the parental Illumina reads to the corresponding haplogenome to investigate whether the smaller than expected haplogenome assembly size might be due to unassembled genomic regions. We observed mapping rates of 98.73% and 99.10% (93.79% and 92.91% properly paired), respectively (Supplementary Table S3), indicating that it is unlikely that major genomic regions are missing in the haplogenome assemblies.

Genome scaffolding

To curate incorrectly assembled contigs, contig breakpoints were inferred based on long-read depth support and used to split suspicious contigs before scaffolding. The parental genetic linkage maps yielded a set of 3,125 (for the *E. urophylla* haplogenome) and 3,129 (*E. grandis* haplogenome) unique SNP markers to anchor contigs into pseudo-chromosome level scaffolds. The anchoring rate for both haplogenome assemblies was greater than 88.0% 2 and a BUSCO completeness score of at least 95.3% was obtained for contigs anchored to one of the eleven chromosomes. Dot-plot visualization of the haplogenome alignment confirmed high levels of collinearity between the assembled haplogenomes (Supplementary Figure S3 and Supplementary Figure S4). ALLMAPS was able to orientate 299 *E. urophylla* and 262 *E. grandis* contigs with two or more markers each., while 52 contigs for *E. urophylla* and 49 for *E. grandis* only had one marker and were placed without orientation 2. A total of 1,067 contigs (corresponding to 63.37 Mb) of the *E. urophylla* and 1,268 contigs (67.78 Mb) of the *E. grandis* haplogenome assembly could not be anchored 2 of which 863 (9.71 Mb) and 1,051 contigs (11.86 Mb) were smaller than 50 kb (Supplementary Table S4) and contained no markers.

Most of the anchored assembly had a high level of congruence between the genetic and physical maps as indicated by the Pearson's correlation coefficient (ρ) being close to -1 or 1, with the weakest correlation being ρ = 0.965 (Supplementary Figure S5) for *E. urophylla* and ρ = 0.938 for *E. grandis* (Supplementary Figure S6). Chromosome 3 and 5 differed from the *E. grandis* v2.0 reference genome by more than 20 Mb (Supplementary Figure S7), which could not be explained by a single missing genomic segment (Supplementary Figure S3). To investigate this, we aligned all unplaced scaffolds to the *E. grandis* v2.0 reference genome but did not observe any chromosomal preference for unplaced scaffolds (Supplementary Figure S8). This suggested that the chromosomal size differences were not due to scaffolds not being placed to those chromosomes (Supplementary Figure S8).

Genome annotation

To further examine whether the smaller haplogenome assembly size is due to a difference in repeat content, we annotated repeat elements with RepeatMasker. A total of 48.34% of the *E. urophylla* haplogenome assembly comprised of repetitive elements, whereas it was 49.06% for the *E. grandis* haplogenome (Supplementary Table S5). In both cases, LTR retrotransposons were the most prevalent repetitive element, making up more than 21% of the assembled haplogenomes (Supplementary Table S5). DNA transposons made up 6% of the haplogenomes. These results are similar to previous repeat annotations for the v2.0 *E. grandis* reference assembly [20], Table 1). We used LTR retriever to visualize the distribution of various LTR retrotransposon types (in bins of 300 kb Figure 2). LTR retriever, which is more sensitive for detection of LTR retrotransposons than RepeatModeler, identified 29.08% and 29.25% of the *E. grandis* and *E. urophylla* haplogenomes respectively, as LTR retrotransposons. Direct comparison of the LTR retrotransposon distribution pattern between *E. grandis* and *E. urophylla* was not possible as the assembled chromosomes differ in size, but there was good relative conservation in pattern with few notable exceptions e.g., on Chromosome 2 (Figure 2).

Structural (*de novo*) annotation resulted in 39,849 and 37,942

Table 1. Assembly and annotation statistics for the *E. urophylla* and *E. grandis* haplogenomess compared to the previous *E. grandis* genome assembly

	E. grandis v2.0	*E. grandis*	*E. urophylla*
Type of sequencing	BAC end cloning (ABI)	Illumina + ONP	Illumina + ONP
Genome coverage[a]	6.73x	54.01x (ONP)	50.25x (ONP)
Primary assembly:			
Number of contigs	32,724	793	654
Total number bases in contigs	691.43 Mb	568.46 Mb	546.11 Mb
Contig N50 length	67.25 kb	3.91 Mb	4.41 Mb
Contig L50	2,261	38	36
Total contigs >50 kb	288	387	368
Validated contigs (Polar_Star):			
Number of contigs	-	1,579	1,418
Total number bases in contigs	-	566.72 Mb	544.51 Mb
Contig N50 length	-	2.42 Mb	1.93 Mb
Contig L50	-	74	83
Total contigs >50 kb	-	522	547
Assembly BUSCO completeness[b]	98.00%	98.30%	97.90%
Number scaffolds	4,951	1,279	1,078
Total number of bases scaffolded[c]	612.60 Mb	498.98 Mb	481.16 Mb
Scaffold N50	53.80 Mb	43.82 Mb	42.45 Mb
Scaffold L50	5	6	6
BUSCO completeness[d]	98.00%	98.30%	97.90%
GC content	39.99%	39.46%	39.44%
Repeat content	44.50%	49.06%	48.34%
Number of genes	36,376	39,849	37,942
Annotation BUSCO completeness[e]	99.10%	94.60%	95.80%

[a] Coverage based on 650 Mb genome size for *E. grandis* and *E. urophylla*.
[b] BUSCO completeness scores of contig level assembly.
[c] Total number of bases scaffolded onto one of the eleven chromosomes.
[d] BUSCO completeness scores of all scaffolds (including unplaced scaffolds).
[e] BUSCO completeness of gene annotation

Table 2. Summary statistics for parental linkage maps (gra_allmap and uro_allmap) and final consensus anchoring of the *E. urophylla* and *E. grandis* haplogenome contigs. A greater weight (indicated with w) was given to the linkage map of the species corresponding to the haplogenome being scaffolded. Scaffolds that contain no SNP markers or had ambiguous placements were counted as unplaced. Marker density (measured as number of markers per Mb) represents the sum of unique markers in the two linkage maps.

E. urophylla	**gra_allmap (w=1)**	**uro_allmap (w=2)**	**Anchored**	**Unplaced**
Linkage Groups	11	11	11	n.a.
Markers (unique)	1,577	1,573	3,125	25
Average markers per Mb	3.5	3.5	6.5	0.4
N50 Scaffolds	76	79	81	2
Scaffolds	311	299	351	1,067
Scaffolds with 1 marker	83	80	52	13
Scaffolds with 2 markers	51	53	42	4
Scaffolds with 3 markers	41	37	44	0
Scaffolds with >=4 markers	136	129	213	1
**Total bases**	**448,984,013**	**447,297,011**	**481,132,251**	**63,374,165**
Percent of genome	82.5%	82.1%	88.4%	11.6%

E. grandis	**gra_allmap (w=2)**	**uro_allmap (w=1)**	**Anchored**	**Unplaced**
Linkage groups	11	11	11	n.a.
Markers (unique)	1,588	1,575	3,129	34
Average markers per Mb	3.3	3.4	6.3	0.5
N50 Scaffolds	72	72	73	1
Scaffolds	283	263	311	1,268
Scaffolds with 1 marker	62	60	49	21
Scaffolds with 2 markers	46	33	26	3
Scaffolds with 3 markers	32	32	30	1
Scaffolds with >=4 markers	143	138	206	1
**Total bases**	**477,075,775**	**464,179,728**	**498,948,047**	**67,775,781**
Percent of genome	84.2%	81.9%	88.0%	12.0%

gene models for the *E. grandis* and *E. urophylla* haplogenomes, respectively (Table 1 and Supplementary Table S6). BUSCO completeness scores of 94.6% and 95.8% was obtained for the *E. grandis* and *E. urophylla* structural annotation models (Table 1). Functional annotation based on similarity searches or gene family assignment was possible for 35,572 and 33,915 structural gene models of *E. grandis* and *E. urophylla* (Supplementary Table S6).

Structural variant analysis

E. grandis and *E. urophylla* are in the same section (*Latoangulatae*) and subgenus *Symphyomyrtus* but have non-overlapping natural ranges with unique adaptations such as greater resistance to fungal pathogens in *E. urophylla*, which has a more tropical distribution. Genetic linkage mapping has suggested high collinearity of their genomes [18, 63, 19], but a direct fine-scale comparison of genome synteny between these species has not been possible. Using the SyRI the whole-genome comparison tool, we revealed that a total of 257 Mb was syntenic between the two haplogenome assemblies, while 262.22 and 374.85 Mb were identified as rearranged in the *E. grandis* and *E. urophylla* haplogenomes, respectively (Figure 2, Figure 3, Supplementary Table S7, Supplementary Figure S9). In comparison, 318 Mb was syntenic between the *E. grandis* haplogenome and the *E. grandis* v2.0 reference genome (Supplementary Table S7, Supplementary Figure S9), but due to the difference in overall assembly size and methods used in the two studies, it is not possible to compare the genomic proportions. The regions rearranged between the haplogenomes included 189 inversions and 10,526 translocations (Figure 3, Supplementary Figure S9, Supplementary Table S7, Supplementary Table S9 and Supplementary Table S10). In addition, there were 16,865 duplications in the *E. grandis* and 21,149 duplications in the *E. urophylla* haplogenome (Figure 3, Supplementary Figure S9 and Supplementary Table S7). Together these results suggest that despite high collinearity previously reported for these species and observed here for the *E. grandis* and *E. urophylla* haplogenomes, extensive fine-scale rearrangements exist that have not been detected in previous studies.

Next, we investigated genome sequence divergence in syntenic regions, designated as "local variants" by SyRI, comprising 65.26 Mb and 66.45 Mb in the *E. grandis* and *E. urophylla* haplogenomes, respectively. These local variants (excluding SNPs) ranged from 1 bp (indels) to 3.09 Mb (highly diverged regions, HDR, Figure 3C). SNPs were the most prevalent class of local variants in terms of number, with 8.3 million SNPs between the *E. grandis* and *E. urophylla* haplogenomes, followed by small insertions and deletions (Supplementary Table S8). In terms of the total bases affected, highly diverged regions and copy gain/losses made up 9.6 Mb and 38 – 40 Mb of the haplogenome assemblies. Although there is a greater number of local variants compared to SVs, local variants made up 13.8% of the *E. urophylla* and 13.1% of the *E. grandis* chromosomal assembly compared to 54.5% and 75.1% in SV. This suggests that although local variants are more numerous, structural variants have a larger impact on genome architecture. This was also revealed in similar studies in tomato[8] and grape [64].

We performed gene-based synteny analysis between the *E. grandis* and *E. urophylla* haplogenomes, which confirmed high collinearity between the haplogenomes, with 23,390 gene pairs in 238 syntenic blocks (average 98.28 gene pairs per syntenic block with min = 4 gene pairs and max = 1296 gene pairs, Figure 4). A total of 227 blocks had 10 or more homologous gene pairs and 175 blocks had 30 or more gene pairs derived from the two haplogenomes. Of the 227 blocks, 86 blocks (8,114 genes and 37.88% of gene synteny blocks) are rearranged between the haplogenomes as inversions or translocations. The top GO enriched terms within these blocks belonged to regulation of transcription, DNA binding transcription factor activity and mRNA binding (a full list of enriched GO terms can be found in Supplementary Figure S10).

Discussion

We have assessed the use of a trio-binning strategy to assemble high-quality haplogenomes in an F1 hybrid of two important eucalypt tree species as a starting point towards investigating pan-genome variation within and between these species. The high level of heterozygosity in the F1 hybrid enabled discrimination of almost all parental long reads and independent assembly of the two parental haplogenomes. These haploid assemblies, the first of their kind for a forest tree species, allowed us to circumvent the problem of co-assembly of alternative haplotypes which has presented a challenge for the assembly of highly heterozygous tree genomes, especially in intergenic DNA where complex structural variants from partially overlapping haplotypes may be co-assembled into a mosaic sequence [20, 19]. Furthermore, the high coverage of long reads (50X per haplogenome) and the long-read length (N50 >27 Kb) allowed us to assemble across complex repeat structures leading overall to highly contiguous assemblies (contig N50 of 2.42 Mb for *E. grandis* and 1.93 Mb for *E. urophylla*). Intriguingly, we find that, despite having very high BUSCO completeness scores (>97.9%), the assembled haplogenomes (567 Mbp and 545 Mbp) were substantially smaller than the previous diploid reference genome assembly of 691.4 Mb [20, 19] and the 640 Mb flow cytometry estimate [61]. High-density SNP genetic linkage maps enabled further improvement of haplogenome assembly contiguity (scaffold N50 >42 Mbp). Finally, we performed the first fine-scale structural and gene-based comparison for any two eucalypt genomes and show that SVs are more prevalent than detected in previous studies, but follow a similar class distribution pattern as in other plants with inversion events the least frequent, followed by translocation events and duplications being the most frequent [58, 65].

Trio-binning of a highly heterozygous F1 hybrid genome

The trio-binning strategy [66] allowed successful discrimination of the long reads derived from the *E. urophylla* and *E. grandis* haplogenomes. A total of 99.98% of the sequenced read bases could be assigned to one of the two haplo-bins, with only a small proportion (0.014%) of mostly shorter nanopore reads was not assigned to bins (N50 = 1,385 bp for un-binned vs N50 27.5 kb for binned reads). The long-read data was split 51.80% vs 48.18% for *E. grandis* and *E. urophylla*, respectively, Supplementary Table S3), matching the assembly sizes, but it is not clear whether this can be generalized for individuals of the two species. Stringent cross-mapping of the parental short-read data to the two haplogenomes revealed, as expected, lower mapping rates to the opposite haplogenome (average 93.35% vs 84.94%, Supplementary Table S3) supporting that we have efficiently separated the haplogenome reads from the two species. The low level of BUSCO duplication in the assembled haplogenomes (less than 3.3%; Supplementary Figure S2) compared to 13.9% reported for a recent diploid *E. pauciflora* assembly [67], supports that the haplotype binning was highly efficient. We further validated the size of phased blocks, as well as phase origin (Supplementary Note 1) and found that the haplogenome assemblies had very low haplotype switch error rates (lower than 0.033%) confirming the accuracy of haplotype separation. Together these results suggest that the trio-binning approach was highly efficient and accurate in the heterozygous F1 hybrid genome.

Haplotype separation is known to improve with higher levels of heterozygosity [66, 68]. We observed high heterozygosity for both pure-species parents (2.14% for *E. grandis* and 2.63% for *E. urophylla*), and as expected, heterozygosity was substantially higher in the F1 hybrid offspring (estimated to be 3.46%; Supplementary Figure S1). Such high heterozygosity levels are expected for outcrossed organisms such as eucalypts [69, 70]. Successful haplotype separation of an F1 hybrid of species within the same section of Myrtaceae (*Latoangulatae*) suggests that application of trio-binning for haplotype separation should be successful for most other viable *Eucalyptus* F1 hybrid combinations. In addition, the high heterozygosity observed in the pure species parents suggests that haplotype binning will also be successful in intraspecific crosses of *Eucalyptus* as the trio-binning strategy has been demonstrated to be efficient at much lower levels of heterozygosity (0.9% in the case of a F1 Brahman x Angus cattle hybrid and 1.36% for *A. thaliana*; [66]).

We note that the haplogenome assembly sizes, 546/481 Mb for *E. urophylla* and 568/498 Mb for *E. grandis* (total/scaffolded size) were much smaller than that of the current *E. grandis* v2.0 reference genome (691/612 Mb, [20, 19] and previous estimates (640

Mb) based on flow cytometry [61]. K-mer based genome size estimates of the parental reads predicted diploid genome sizes of 443 Mb for *E. urophylla*, 482 Mb for *E. grandis* and 478 Mb for the F1 hybrid (Supplementary Figure S1), which agreed with the scaffolded genome sizes of the two haplogenome assemblies. This apparent discrepancy was also observed in *E. pauciflora*, where k-mer based estimates were 408 Mb compared to the final 595 Mb assembly [67]. The total assembly sizes of the two haplogenomes were therefore approximately 70 - 100 Mb smaller than previous flow cytometry estimates for the two species and the total scaffolded sizes were 140 - 160 Mbp smaller than expected. This size discrepancy may be explained by several factors, which we explore below.

First, to exclude the possibility that the smaller assembly size was due to a portion of sequencing reads not being assembled, i.e., that we failed to assemble parts of the haplogenomes, we aligned the parental Illumina reads to the corresponding parental haplogenome assembly. We also aligned the raw short- and long-reads and the haplogenome assemblies to the *E. grandis* v2.0 reference genome to make sure all v2.0 genomic regions had sequencing coverage (Supplementary Note 2). This revealed that some regions had very high sequencing depth relative to the *E. grandis* v2.0 reference genome (Supplementary Note 2) presumably due to highly repetitive sequence content in those regions. More than 98.7% of parental Illumina reads aligned to their corresponding parental haplogenome, which suggests that almost all of the sequences in the parental genomes (that are amenable to Illumina sequencing) are represented in the haplogenomes (Supplementary Table S2), although it is possible that the regions with high sequencing depth represent repetitive regions that are collapsed in the haplogenome assemblies. To further investigate this possibility, we confirmed that the repeat content of the haplogenomes was not lower than that reported in the *E. grandis* v2.0 diploid reference assembly. In fact, the repeat content for the *E. urophylla* and *E. grandis* haplogenomes (48.34% and 49.06%, respectively, Table 1) was higher than that reported for the *E. grandis* v2.0 assembly (44.50%, [20] and for the more recent *E. pauciflora* assembly (44.77%, [67]). This suggests that the observed size difference is most probably not due to the collapse of repetitive regions during haplogenome assembly. Rather, the slightly higher repeat content of our haplogenome assemblies probably reflect our ability to better assemble across such repeats using long-read technology in haplo-assemblies vs short-read/Sanger sequencing previously used for these highly heterozygous genomes. Previous size estimates were probably somewhat inflated in size due to the possible co-assembly of partially overlapping alternative haplotypes highly heterozygous regions distributed throughout the genome. Our analysis showed that Chromosomes 3 and 5 in the haplogenome assemblies were 20 Mb smaller than the corresponding chromosomes in the diploid *E. grandis* v2.0 assembly.

Genetic linkage maps support high scaffolding rates

Overall, 88.4% and 88.0% of the haplogenome assemblies was anchored into 11 pseudo-chromosomes for *E. urophylla* and *E. grandis*. However, there are some limits to using ALLMAPS for genome scaffolding as the program cannot identify and separate duplicated regions that are misassembled or collapsed by the genome assembler due to high similarity [37]. In addition, most genetic linkage maps contain regions such as centromeres with no or very low recombination and few DNA markers for anchoring and orientation of contigs. Many of the unanchored contigs may contain difficult to assemble, centromeric or other non-recombinogenic regions devoid of mapped DNA markers (average 0.4 and 0.5 markers per Mb for unanchored vs 6.5 and 6.3 markers per Mb for anchored *E. urophylla* and *E. grandis* contigs, respectively, Table 2). The N50 of the unanchored contigs was 324 kb, which was smaller than the average marker spacing in those regions (Supplementary Table S4). Thus, integration of additional proximity ligation or optical mapping data may lead to inclusion of some of the remaining unplaced contigs that had few markers to place or orient them. Despite this limitation we were able to produce eleven pseudo-chromosome scaffolds for each of the haplogenomes owing to the high density of SNP markers in the parental maps and the quality of the genetic maps as evidenced in collinearity of markers between the genetic map and the de novo assembled contigs, as well as high collinearity between the scaffolded assembly and the genetic linkage maps (Pearson's correlation of $\rho = 0.938$ to $\rho = 1.00$; Supplementary Figure S5 and Supplementary Figure S6).

Structural variants between *E. urophylla* and *E. grandis*

To our knowledge, this is the first genome-wide comparison of synteny and structural rearrangements between two eucalypt species. In addition, we had the advantage of being able to directly compare the two haplogenomes from the same F1 hybrid individual assembled using the same method. Using SyRI we found that 53.39% (256.88 Mb) of the 481.16 Mb chromosomal assembly of *E. urophylla* and 51.45% (256.75 Mb) of the 498.97 Mb chromosomal assembly of *E. grandis* was syntenic (Supplementary Table S7). We were able to identify 48,729 SVs between the two haplogenomes, with a 103.62 Mb difference between the two haplogenomes due to duplications (Supplementary Table S7). As seen in previous studies using SyRI for SV calling, we found that inversions were the smallest group of SVs in terms of number, followed by translocations, with duplications being the most abundant (189 inversions, 10,526 translocations and 38,014 duplications, Supplementary Table S7; [58, 65]. Using unfolded site frequency spectrum of SVs, [64] found that there is purifying selection against SVs, and that there is stronger purifying selection against inversions and translocations compared to duplications as they have a more deleterious effect compared to duplications [64]. Stronger purifying selection against inversions and translocations in our haplogenome assemblies may therefore explain the lower frequency of these two classes of SV, however this will need to be tested in future sequencing projects including population-wide tracking of SVs.

With additional genome sequences for *E. grandis* and *E. urophylla*, a pan-genome reference assembly could be constructed as was done for *Arabidopsis* [65] and tomato [8, 71]. SyRI identifies SVs and local variants using three main steps: 1) identify syntenic alignments, 2) identify inverted, duplicated and translocated alignments and 3) identify "local variants" within alignment blocks. As such, there is a hierarchy of variation where local variants are found within alignment blocks, be they syntenic or rearranged regions. However, when looking for the functional effects of local and larger structural variants, it is important to note the hierarchy of genomic rearrangements, as local variants within rearranged regions show different inheritance patterns to those in syntenic regions. SVs can influence recombination studies as rearrangement hotspots typically have lower synteny and reduced recombination rates [65]. In addition, SVs can influence gene expression directly or indirectly making their functional interpretation harder [58].

Surprisingly, despite the high completeness, we found that the total assembled size of each of the haplogenomes is substantially smaller than that of the *E. grandis* v2.0 reference genome and previous flow cytometry estimates. We propose that the size difference is not due to collapse of the repeat content of the haplogenome assemblies, but rather due to possible overestimation of the *E. grandis* v2.0 genome assembly as a result of inclusion of partially overlapping alternative haplotypes in highly heterozygous regions of the diploid genome assembly. However, resolving this discrepancy will require further de novo genome assemblies for *E. grandis*, possibly including resequencing using long read technology to update the genome assembly of the reference BRASUZ1 individual, as has been performed for some reference genomes that were originally assembled with Sanger sequencing data [72].

Finally, we provide the first genome-wide SV between *E. urophylla* and *E. grandis*. We identify 48,729 SVs, ranging in size from 100 bp to 4.91 Mb. Some of these variants are large enough to be able to cover multiple genes and future studies will focus on un-

derstanding the genomic context and functional implications of these variants. An added advantage of this study is the reduced false discovery of SVs which may be introduced when comparing genomes assembled using different pipelines.

Conclusions

We have produced phased, haplogenome assemblies of an interspecific F1 hybrid using a trio-binning approach and performed the first genome-wide analysis of genome synteny between two key tree species used in plantation forestry, *E. grandis* and *E. urophylla*. This revealed a large number of previously undescribed genome structural variants as a step towards understanding genome structural evolution in this iconic genus of fast-growing woody perennials. The haplogenome resource data provides the first insight into haplotype diversity in F1 hybrids and, with additional haplogenomes to be sequenced, this will lead to a better understanding of the genetic basis of hybrid compatibility and superiority. This work is a pilot study towards understanding pan-genome variation in *Eucalyptus* that can be used for tree improvement. The project also produced the first draft, near complete genome assembly for *E. urophylla*, a key tropical eucalypt with an interesting island colonization history.

Additional Files

Supplementary Figure S1. Genome size estimates for the (A) *E. urophylla*, (B) *E. grandis* and (C) the *E. urophylla* x *E. grandis* F1 hybrid genomes.
Supplementary Figure S2. Benchmarking Universal Single-Copy Orthologs (BUSCO) completeness scores for both haplogenome assemblies as well as the currently available *E. grandis* v2.0 reference genome.
Supplementary Figure S3. Alignment of placed haplogenome scaffolds to the *E. grandis* v2.0 reference genome.
Supplementary Figure S4. Alignment between the *E. grandis* and *E. urophylla* scaffolded haplogenome assemblies.
Supplementary Figure S5. Pseudochromosomes of *E. urophylla* haplogenome, reconstructed from two genetic linkage input maps – uro.allmap and gra.allmap, with unequal weights (2 and 1 respectively).
Supplementary Figure S6. Pseudochromosomes of *E. grandis* haplogenome, reconstructed from two genetic linkage input maps – gra.allmap and uro.allmap, with unequal weights (2 and 1 respectively).
Supplementary Figure S7. Scaffolded chromosome sizes of the *E. grandis* v2.0 and the scaffolded *E. grandis* and *E. urophylla* haplogenome assemblies.
Supplementary Figure S9. Syntenic and rearranged regions between the *E. grandis* v2.0, *E. grandis* and *E. urophylla* haplogenomes for all eleven chromosomes.
Supplementary Figure S10. Enriched gene ontology (GO) terms for inverted and translocated gene alignment blocks.
Supplementary Figure S11. Hap-mer blob plot of the *E. grandis* and *E. urophylla* haplogenome assemblies.
Supplementary Figure S12. Evaluation of haplotype phase blocks. All hap-mer information was generated with Merqury v1.1 [68].
Supplementary Note 1. Hap-mer based phasing completeness assessment.
Supplementary Note 2. Read and assembly alignment and validation of high peak content
Supplementary Table S1. Illumina sequencing results.
Supplementary Table S2. Nanopore sequencing results for the F1 hybrid individual.
Supplementary Table S3. Summary statistics for long-read binning using the parental short reads.
Supplementary Table S4. Summary statistics of placed and unplaced contigs after scaffolding with ALLMAPS for the *E. urophylla* and *E. grandis* haplogenomes respectively.
Supplementary Table S5. Repeat element content of assembled haplogenomes.
Supplementary Table S6. Haplogenome annotation statistics.
Supplementary Table S7. Number and total length of syntenic and rearranged regions in the *E. grandis* and *E. urophylla* haplogenomes.
Supplementary Table S8. Number and total length of local sequence variation in syntenic and rearranged region in the *E. grandis* and *E. urophylla* haplogenomes.
Supplementary Table S9. Inversions larger than 50 kb between the *E. grandis* and *E. urophylla* haplogenomes.
Supplementary Table S10. Translocations between the *E. grandis* and *E. urophylla* haplogenomes that are larger than 50 kb.
Supplementary Table S11. Phase block statistics of the *E. grandis* and *E. urophylla* haplo-genome assemblies.
Supplementary Table S12. *E. grandis* and *E. urophylla* high coverage bin content.

Availability of source code and requirements

All scripts are available at: `https://gitlab.com/Anneri/eucalyptus-haplogenome-synteny`.

Data Availability

Illumina DNA sequencing data was uploaded at NCBI SRA under BioProject: PRJNA870669. High density genetic linkage maps are available on github. The haplogenome assemblies were uploaded to the NCBI database and can be accessed with accession no. JAOPUP000000000 and JAOPUO000000000. All supporting data such as repeat element libraries, genome annotation files, synteny analyses output files etc. are available upon request.

Declarations

List of abbreviations

BAC: bacterial artificial chromosome, BLAST: Basic Local Alignment Search Tool, BUSCO: Benchmarking Universal Single-Copy Orthologs, BWA: BurrowsWheeler Aligner, CDS: coding sequence, Chr: Chromosome, DUP: duplication, EGR: *E. grandis*, EUR: *E. urophylla*, FDR: false discovery rate, Gb: Gigabase, GO: gene ontology, HDR: highly diverged regions, HMW: high molecular weight, INV: inversion, kb: kilobase, KEGG: Kyoto Encyclopedia of Genes and Genomes, LINE: long interspersed nucleotide element, LRS: Long-read sequencing, LTR: long terminal repeat, Mb: megabase, NCBI: National Center for Biotechnology Information, ONT: Oxford Nanopore Technologies, PacBio: Pacific Biosciences, PE: paired-end, QC: quality control, QUAST: quality assessment tool, QV: quality value, RNA-seq: RNA sequencing, SNP: single nucleotide polymorphism, SRA: Sequence Read Archive, SRS: short read sequencing, SVs: structural variants, SYN: syntenic region, SyRI: Synteny and Rearrangement Identifier, TE: transposable element, TRANS: translocation.

Consent for publication

Not applicable

Competing Interests

The authors declare that they have no competing interests.

Funding

This work was funded by the South African Department of Science and Innovation and Technology Innovation Agency (DSI/TIA, Strategic Grant for Eucalyptus Genomics), the Forestry Sector Innovation Fund (FSIF) and Sappi South Africa through the Forest Molecular Genetics (FMG) Industry Consortium at the University of Pretoria (UP). AL acknowledges MSc bursary support from the

National Research Foundation (NRF) of South Africa (Grant Number MND190406427887) and funding from the UP Postgraduate Studies Abroad Programme.

Author's Contributions

AL performed all analyses in the manuscript and prepared the manuscript. JC constructed genetic linkage maps for genome scaffolding. TD and JLW provided bioinformatic and technical support and advised on data analysis and interpretation throughout the project. TD, EM and JLW co-supervised the project. AAM conceived and supervised the project. All authors read and contributed to the final manuscript.

Acknowledgements

The authors thank the UP Bioinformatics and Computational Biology Centre and University of Connecticut (UConn) Computational Biology Core for bioinformatics and computational biology support. Sappi Forest Research (Howick, South Africa) kindly provided the plant materials used in the study.

References

1. Grierson CS, Barnes SR, Chase MW, Clarke M, Grierson D, Edwards KJ, et al. One hundred important questions facing plant science research. New Phytol 2011;192(1):6–12.
2. Grattapaglia D, Kirst M. Eucalyptus applied genomics: from gene sequences to breeding tools. New Phytol 2008;179(4):911–929.
3. de Assis TF. Production and use of Eucalyptus hybrids for industrial purposes. Noosa, Queensland, Australia; 2000.
4. Rezende GDSP, de Resende MDV, de Assis TF. Chapter 16. In: Eucalyptus Breeding for Clonal Forestry Forestry Sciences, Springer; 2014. p. 393–424.
5. Grattapaglia D, Silva-Junior OB, Resende RT, Cappa EP, Muller BSF, Tan B, et al. Quantitative Genetics and Genomics Converge to Accelerate Forest Tree Breeding. Front Plant Sci 2018;9:1693.
6. Zheng GX, Lau BT, Schnall-Levin M, Jarosz M, Bell JM, Hindson CM, et al. Haplotyping germline and cancer genomes with high-throughput linked-read sequencing. Nat Biotechnol 2016;34(3):303–11.
7. Jiao WB, Schneeberger K. The impact of third generation genomic technologies on plant genome assembly. Curr Opin Plant Biol 2017;36:64–70.
8. Alonge M, Wang X, Benoit M, Soyk S, Pereira L, Zhang L, et al. Major Impacts of Widespread Structural Variation on Gene Expression and Crop Improvement in Tomato. Cell 2020;182(1):145–161 e23.
9. Ogawa D, Nonoue Y, Tsunematsu H, Kanno N, Yamamoto T, Yonemaru JI. Discovery of QTL Alleles for Grain Shape in the Japan-MAGIC Rice Population Using Haplotype Information. G3 (Bethesda) 2018;8(11):3559–3565.
10. Ogawa D, Yamamoto E, Ohtani T, Kanno N, Tsunematsu H, Nonoue Y, et al. Haplotype-based allele mining in the Japan-MAGIC rice population. Sci Rep 2018;8(1):4379.
11. Motazedi E, Finkers R, Maliepaard C, de Ridder D. Exploiting next-generation sequencing to solve the haplotyping puzzle in polyploids: a simulation study. Brief Bioinform 2018;19(3):387–403.
12. Bevan MW, Uauy C, Wulff BB, Zhou J, Krasileva K, Clark MD. Genomic innovation for crop improvement. Nature 2017;543(7645):346–354.
13. Kyriakidou M, Tai HH, Anglin NL, Ellis D, Stromvik MV. Current Strategies of Polyploid Plant Genome Sequence Assembly. Front Plant Sci 2018;9:1660.
14. Sherman RM, Salzberg SL. Pan-genomics in the human genome era. Nat Rev Genet 2020;21(4):243–254.
15. Bayer PE, Golicz AA, Scheben A, Batley J, Edwards D. Plant pan-genomes are the new reference. Nat Plants 2020;6(8):914–920.
16. Brondani RPV, Brondani C, Tarchini R, Grattapaglia D. Development, characterization and mapping of microsatellite markers in Eucalyptus grandis and E. urophylla. Theoretical and Applied Genetics 1998;97(5-6):816–827.
17. Marques M, Brondani V, Grattapaglia D, Sederoff R. Conservation and synteny of SSR loci and QTLs for vegetative propagation in four Eucalyptus species. Theor Appl Genet 2002;105(2-3):474–478.
18. Hudson CJ, Kullan ARK, Freeman JS, Faria DA, Grattapaglia D, Kilian A, et al. High synteny and colinearity among Eucalyptus genomes revealed by high-density comparative genetic mapping. Tree Genetics & Genomes 2011;8(2):339–352.
19. Bartholome J, Mandrou E, Mabiala A, Jenkins J, Nabihoudine I, Klopp C, et al. High-resolution genetic maps of Eucalyptus improve Eucalyptus grandis genome assembly. New Phytol 2015;206(4):1283–96.
20. Myburg AA, Grattapaglia D, Tuskan GA, Hellsten U, Hayes RD, Grimwood J, et al. The genome of Eucalyptus grandis. Nature 2014;510(7505):356–62.
21. Ho SS, Urban AE, Mills RE. Structural variation in the sequencing era. Nat Rev Genet 2020;21(3):171–189.
22. Koren S, Walenz BP, Berlin K, Miller JR, Bergman NH, Phillippy AM. Canu: scalable and accurate long-read assembly via adaptive k-mer weighting and repeat separation. Genome Res 2017;27(5):722–736.
23. Shirasawa K, Esumi T, Hirakawa H, Tanaka H, Itai A, Ghelfi A, et al. Phased genome sequence of an interspecific hybrid flowering cherry, 'Somei-Yoshino' (Cerasus x yedoensis). DNA Res 2019;26(5):379–389.
24. Zhu T, Wang L, You FM, Rodriguez JC, Deal KR, Chen L, et al. Sequencing a Juglans regia x J. microcarpa hybrid yields high-quality genome assemblies of parental species. Hortic Res 2019;6:55.
25. Marcais G, Kingsford C. A fast, lock-free approach for efficient parallel counting of occurrences of k-mers. Bioinformatics 2011;27(6):764–70.
26. Ranallo-Benavidez TR, Jaron KS, Schatz MC. GenomeScope 2.0 and Smudgeplot for reference-free profiling of polyploid genomes. Nat Commun 2020;11(1):1432.
27. Kim D, Song L, Breitwieser FP, Salzberg SL. Centrifuge: rapid and sensitive classification of metagenomic sequences. Genome Res 2016;26(12):1721–1729.
28. Wood DE, Lu J, Langmead B. Improved metagenomic analysis with Kraken 2. Genome Biol 2019;20(1):257.
29. Zimin AV, Puiu D, Luo MC, Zhu T, Koren S, Marcais G, et al. Hybrid assembly of the large and highly repetitive genome of Aegilops tauschii, a progenitor of bread wheat, with the MaSuRCA mega-reads algorithm. Genome Res 2017;27(5):787–792.
30. Gurevich A, Saveliev V, Vyahhi N, Tesler G. QUAST: quality assessment tool for genome assemblies. Bioinformatics 2013;29(8):1072–5.
31. Mikheenko A, Prjibelski A, Saveliev V, Antipov D, Gurevich A. Versatile genome assembly evaluation with QUAST-LG. Bioinformatics 2018;34(13):i142–i150.
32. Simao FA, Waterhouse RM, Ioannidis P, Kriventseva EV, Zdobnov EM. BUSCO: assessing genome assembly and annotation completeness with single-copy orthologs. Bioinformatics 2015;31(19):3210–2.
33. Seppey M, Manni M, Zdobnov EM. BUSCO: Assessing Genome Assembly and Annotation Completeness. Methods Mol Biol 2019;1962:227–245.
34. Manni M, Berkeley MR, Seppey M, Simao FA, Zdobnov EM. BUSCO Update: Novel and Streamlined Workflows along with Broader and Deeper Phylogenetic Coverage for Scoring of Eukaryotic, Prokaryotic, and Viral Genomes. Mol Biol

Evol 2021;38(10):4647–4654. https://www.ncbi.nlm.nih.gov/pubmed/34320186.
35. Li H, Durbin R. Fast and accurate short read alignment with Burrows-Wheeler transform. Bioinformatics 2009;25(14):1754–60.
36. Li H, Handsaker B, Wysoker A, Fennell T, Ruan J, Homer N, et al. The Sequence Alignment/Map format and SAMtools. Bioinformatics 2009;25(16):2078–9.
37. Tang H, Zhang X, Miao C, Zhang J, Ming R, Schnable JC, et al. ALLMAPS: robust scaffold ordering based on multiple maps. Genome Biol 2015;16:3.
38. Li H. Minimap and miniasm: fast mapping and de novo assembly for noisy long sequences. Bioinformatics 2016;32(14):2103–10.
39. Cabanettes F, Klopp C. D-GENIES: dot plot large genomes in an interactive, efficient and simple way. PeerJ 2018;6:e4958.
40. Smit A, Hubley R. RepeatModeler Open-1.0.; 2008, http://www.repeatmasker.org/RepeatModeler/.
41. Smit AFA, Hubley R, Green P. RepeatMasker Open-4.0.; 2013, http://www.repeatmasker.org.
42. Ou S, Jiang N. LTR_retriever: A Highly Accurate and Sensitive Program for Identification of Long Terminal Repeat Retrotransposons. Plant Physiol 2018;176(2):1410–1422.
43. Krzywinski M, Schein J, Birol I, Connors J, Gascoyne R, Horsman D, et al. Circos: an information aesthetic for comparative genomics. Genome Res 2009;19(9):1639–45.
44. Mizrachi E, Hefer CA, Ranik M, Joubert F, Myburg AA. De novo assembled expressed gene catalog of a fast-growing Eucalyptus tree produced by Illumina mRNA-Seq. BMC Genomics 2010;11:681.
45. Vining KJ, Romanel E, Jones RC, Klocko A, Alves-Ferreira M, Hefer CA, et al. The floral transcriptome of Eucalyptus grandis. New Phytol 2015;206(4):1406–22.
46. Mizrachi E, Verbeke L, Christie N, Fierro AC, Mansfield SD, Davis MF, et al. Network-based integration of systems genetics data reveals pathways associated with lignocellulosic biomass accumulation and processing. Proc Natl Acad Sci U S A 2017;114(5):1195–1200.
47. Bolger AM, Lohse M, Usadel B. Trimmomatic: a flexible trimmer for Illumina sequence data. Bioinformatics 2014;30(15):2114–20.
48. Kim D, Paggi JM, Park C, Bennett C, Salzberg SL. Graph-based genome alignment and genotyping with HISAT2 and HISAT-genotype. Nat Biotechnol 2019;37(8):907–915.
49. Gremme G, Brendel V, Sparks ME, Kurtz S. Engineering a software tool for gene structure prediction in higher organisms. Information and Software Technology 2005;47(15):965–978.
50. Hoff KJ, Lange S, Lomsadze A, Borodovsky M, Stanke M. BRAKER1: Unsupervised RNA-Seq-Based Genome Annotation with GeneMark-ET and AUGUSTUS. Bioinformatics 2016;32(5):767–769.
51. Lomsadze A, Burns PD, Borodovsky M. Integration of mapped RNA-Seq reads into automatic training of eukaryotic gene finding algorithm. Nucleic Acids Res 2014;42(15):e119.
52. Stanke M, Diekhans M, Baertsch R, Haussler D. Using native and syntenically mapped cDNA alignments to improve de novo gene finding. Bioinformatics 2008;24(5):637–44.
53. Stanke M, Keller O, Gunduz I, Hayes A, Waack S, Morgenstern B. AUGUSTUS: ab initio prediction of alternative transcripts. Nucleic Acids Res 2006;34(Web Server issue):W435–9.
54. Camacho C, Coulouris G, Avagyan V, Ma N, Papadopoulos J, Bealer K, et al. BLAST+: architecture and applications. BMC Bioinformatics 2009;10:421.
55. Caballero M, Wegrzyn J. gFACs: Gene Filtering, Analysis, and Conversion to Unify Genome Annotations Across Alignment and Gene Prediction Frameworks. Genomics Proteomics Bioinformatics 2019;17(3):305–310.
56. Hart AJ, Ginzburg S, Xu MS, Fisher CR, Rahmatpour N, Mitton JB, et al. EnTAP: Bringing faster and smarter functional annotation to non-model eukaryotic transcriptomes. Mol Ecol Resour 2020;20(2):591–604.
57. Kurtz S, Phillippy A, Delcher AL, Smoot M, Shumway M, Antonescu C, et al. Versatile and open software for comparing large genomes. Genome Biol 2004;5(2):R12.
58. Goel M, Sun H, Jiao WB, Schneeberger K. SyRI: finding genomic rearrangements and local sequence differences from whole-genome assemblies. Genome Biol 2019;20(1):277.
59. Tang H, Bowers JE, Wang X, Ming R, Alam M, Paterson AH. Synteny and collinearity in plant genomes. Science 2008;320(5875):486–8.
60. Conesa A, Gotz S, Garcia-Gomez JM, Terol J, Talon M, Robles M. Blast2GO: a universal tool for annotation, visualization and analysis in functional genomics research. Bioinformatics 2005;21(18):3674–6.
61. Grattapaglia D, Bradshaw Jr HD. Nuclear DNA content of commercially important Eucalyptus species and hybrids. Canadian Journal of Forest Research 1994;24(5):1074–1078.
62. Marks RA, Hotaling S, Frandsen PB, VanBuren R. Representation and participation across 20 years of plant genome sequencing. Nat Plants 2021;7(12):1571–1578.
63. Kullan ARK, van Dyk MM, Jones N, Kanzler A, Bayley A, Myburg AA. High-density genetic linkage maps with over 2,400 sequence-anchored DArT markers for genetic dissection in an F2 pseudo-backcross of Eucalyptus grandis × E. urophylla. Tree Genetics & Genomes 2011;8(1):163–175.
64. Zhou Y, Minio A, Massonnet M, Solares E, Lv Y, Beridze T, et al. The population genetics of structural variants in grapevine domestication. Nat Plants 2019;5(9):965–979.
65. Jiao WB, Schneeberger K. Chromosome-level assemblies of multiple Arabidopsis genomes reveal hotspots of rearrangements with altered evolutionary dynamics. Nat Commun 2020;11(1):989.
66. Koren S, Rhie A, Walenz BP, Dilthey AT, Bickhart DM, Kingan SB, et al. De novo assembly of haplotype-resolved genomes with trio binning. Nat Biotechnol 2018;.
67. Wang W, Das A, Kainer D, Schalamun M, Morales-Suarez A, Schwessinger B, et al. The draft nuclear genome assembly of Eucalyptus pauciflora: a pipeline for comparing de novo assemblies. Gigascience 2020;9(1).
68. Rhie A, Walenz BP, Koren S, Phillippy AM. Merqury: reference-free quality, completeness, and phasing assessment for genome assemblies. Genome Biol 2020;21(1):245.
69. Moran GF, Bell JC, Griffin AR. Reduction in levels of inbreeding in a seed orchard of Eucalyptus regnans F. Muall. compared with natural populations. Silvae Genetica 1989;38(1).
70. Gaiotto FA, Bramucci M, Grattapaglia D. Estimation of outcrossing rate in a breeding population of Eucalyptus urophylla with dominant RAPD and AFLP markers. Theoretical and Applied Genetics 1997;95(5-6):842–849.
71. Wang X, Gao L, Jiao C, Stravoravdis S, Hosmani PS, Saha S, et al. Genome of Solanum pimpinellifolium provides insights into structural variants during tomato breeding. Nat Commun 2020;11(1):5817.
72. Nurk S, Koren S, Rhie A, Rautiainen M, Bzikadze AV, Mikheenko A, et al. The complete sequence of a human genome. Science 2022;376(6588):44–53.

Figure legends

Figure 1. Separation of *E. urophylla* and *E. grandis* haplogenomes in the F1 hybrid using a trio-binning strategy. Using whole-genome Illumina short-read sequencing data of the parental genomes, long-read sequencing data of the F1 hybrid offspring is separated based on unique parental k-mers into *E. urophylla* and *E. grandis* haplotype bins (amount of Nanopore sequencing data is indicated in

gigabases (Gb) below each bin, as well as the estimated genome coverage). Reads that contain no unique k-mers were unbinned and kept in their own bin. Long reads were subsequently assembled independently, resulting in fully assembled *E. urophylla* and *E. grandis* haplogenome (total assembly size is shown below the relevant haplogenome and size of assembly scaffolded into eleven chromosomes are indicated in brackets). This figure is adapted from [66] and tree images are from https://rooweb.com.au/.

Figure 2. Synteny and distribution of LTR retrotransposons along the *E. grandis* and *E. urophylla* haplogenome assemblies for eleven scaffolded chromosomes. Syntenic regions are shown between the *E. urophylla* and *E. grandis* haplogenomes in the middle, based on SyRI (see Supplementary Figure S9). LTR retrotransposon distribution is shown for the *E. urophylla* (EUR) and the *E. grandis* (EGR) haplogenome assemblies. From outside to inside, the heatmaps show the distribution of Copia (orange, ranging from 6 to 21.5%), Gypsy (blue, ranging from 1.3 to 26.5%) and unknown (green, ranging from 2.8 to 16.6%) LTR retrotransposons, GC% (37.0 to 43.0%) and gene density (0 to 60.0%) with darker shades representing a higher percentage of retrotransposons within the bin. Chromosome number and size is indicated on the outer circle in megabases.

Figure 3. Size and distribution of structural rearrangements and local variants between the *E. grandis* and *E. urophylla* haplogenomes. (A) Distribution of syntenic regions and structural variants between the *E. grandis* and *E. urophylla* haplogenome assemblies. Links are shown between *E. urophylla* (EUR) chromosomes in blue and and *E. grandis* (EGR) chromosomes in green. Only variants of greater than 10 kilobases as identified by SyRI are shown. Darker links in show change in chromosome number between EUR and EGR. (B) Total size of syntenic and rearranged regions in megabases (Mb) for the *E. grandis* and *E. urophylla* haplogenome (see Supplementary Table S7 and Supplementary Table S8). The size of syntenic or rearranged regions are indicated within the bar in Mb, while the bar colour represents the rearrangement type. (C) Size distribution of rearranged regions (left) and local variants (right) between the *E. grandis* and *E. urophylla* haplogenomes. Size is indicated in base pairs on the y-axis (ranging from one to 4.91 Mb for rearrangements and one to 3.09 Mb for local variants), and the rearrangement type on the x-axis; INV are inversions, DUP are duplications, TRANS are translocations, NOTAL are regions that are not aligned, TDM are tandem repeats, CPG and CPL are copy gains/losses, HDR are highly diverged regions, INS are insertions and DEL are deletions.

Figure 4. Gene synteny between *E. grandis* and *E. urophylla* haplogenome assemblies. (A) Chromosome-scale collinearity between *E. grandis* and *E. urophylla* haplogenome annotations. Lines in light grey indicate syntenic gene blocks, lines in purple indicate inverted gene blocks, blue indicated translocated gene block and dark grey inverted translocated gene blocks. Only block that span greater than 30 gene pairs are shown. (B) Dot-plot alignments of 23,390 gene pairs between the *E. grandis* and *E. urophylla* haplogenome annotations. (C) Bar graph showing syntenic depth of *E. grandis* and *E. urophylla* syntenic blocks. The majority of genes are in a 1 to 1 synteny pattern. All of the graphs were produced in MCScan JCVI v1.1.18 [59].

Figure 4

Click here to access/download;Figure;Figure 4.png

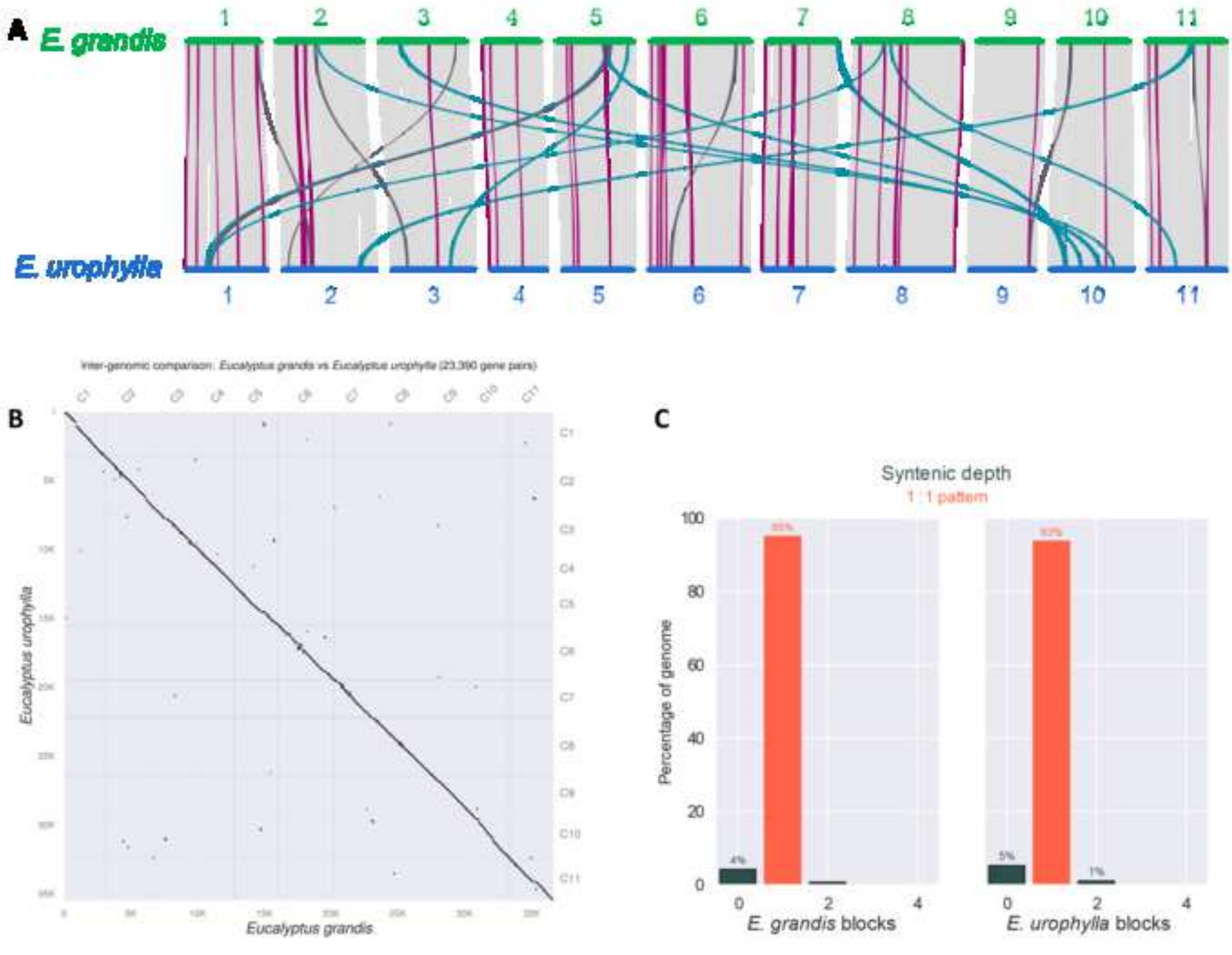

Figure 1

Click here to access/download;Figure;Figure 1.png

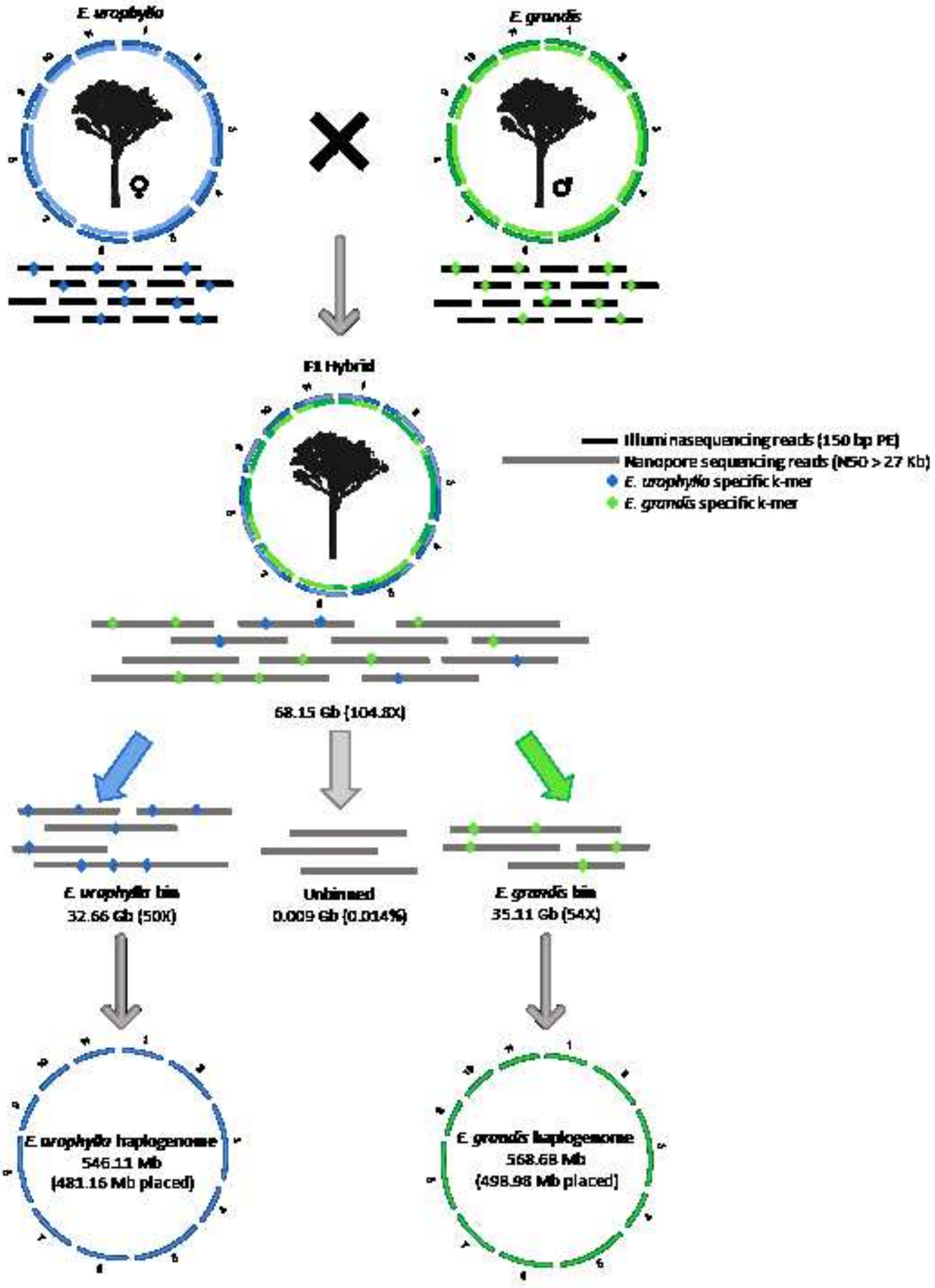

Figure 2

Click here to access/download;Figure;Figure 2.png

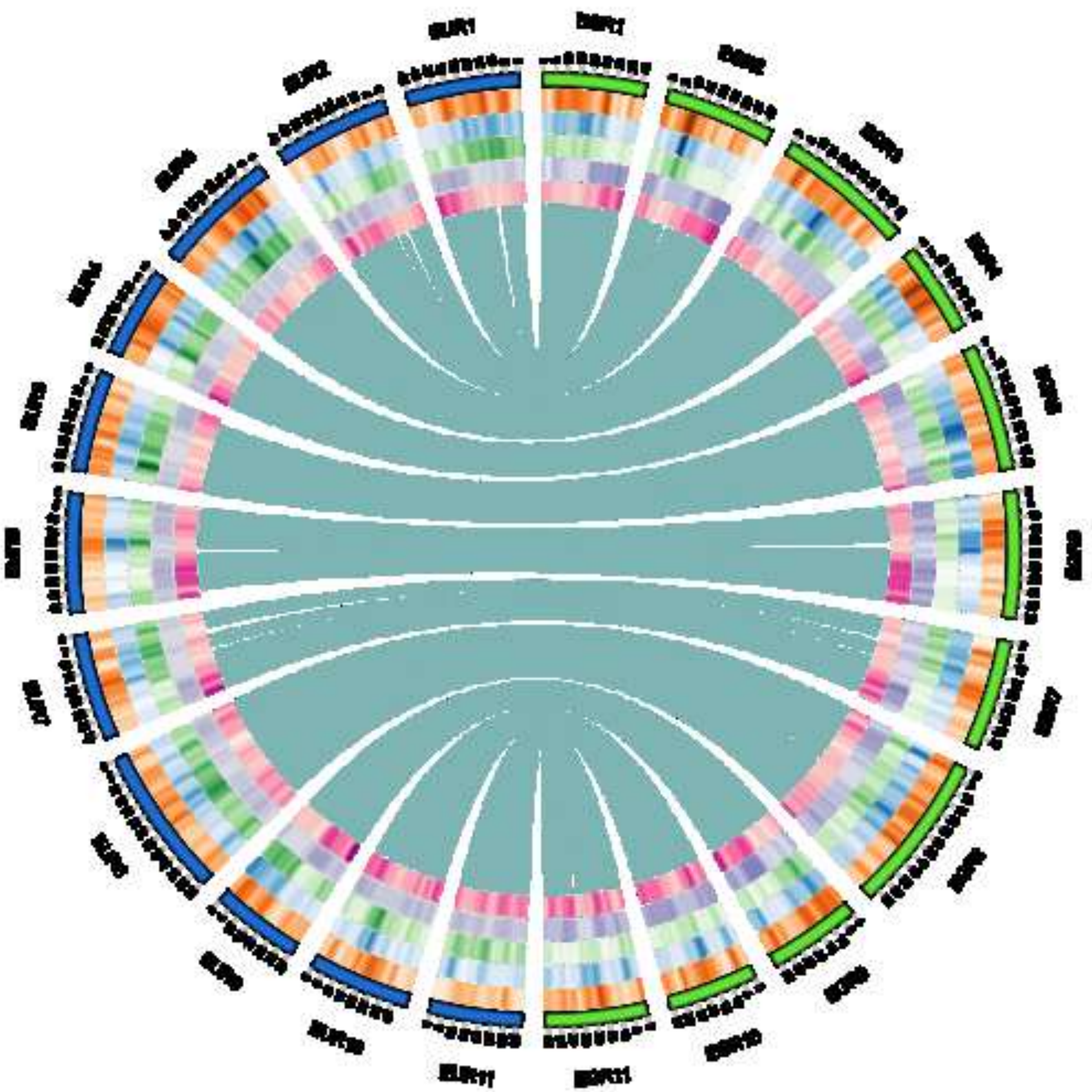

Figure 3 Click here to access/download;Figure;Figure 3.png

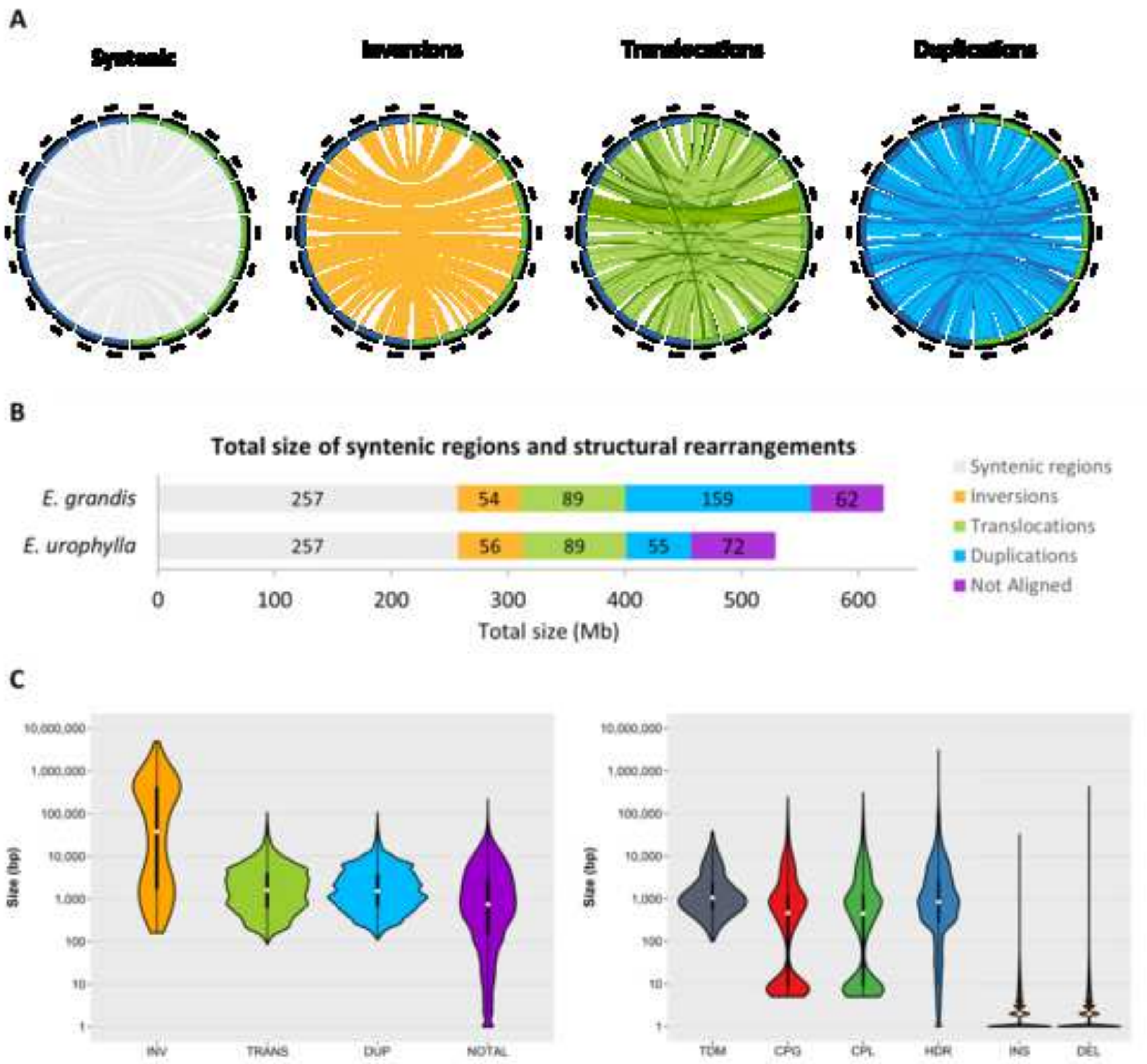

Supplementary Figure S10

Click here to access/download
Supplementary Material
Supplementary Figure S10.png

Supplementary Figure S11

Click here to access/download
Supplementary Material
Supplementary Figure S11.png

Supplementary Figure S12

Click here to access/download
Supplementary Material
Supplementary Figure S12.png

Supplementary Figure S13A

Click here to access/download
Supplementary Material
Supplementary Figure S13A.png

Supplementary Figure S13B

Click here to access/download

Supplementary Material

Supplementary Figure S13B.png

Supplementary Figure S14

Click here to access/download
Supplementary Material
Supplementary Figure S14.png

Supplementary Figure S1

Click here to access/download
Supplementary Material
Supplementary Figure S1.png

Supplementary Figure S3

Click here to access/download
Supplementary Material
Supplementary Figure S3.png

Supplementary Figure S4

Click here to access/download
Supplementary Material
Supplementary Figure S4.png

Supplementary Figure S5

Click here to access/download
Supplementary Material
Supplementary Figure S5.png

Supplementary Figure S6

Click here to access/download
Supplementary Material
Supplementary Figure S6.png

Supplementary Figure S7

Click here to access/download
Supplementary Material
Supplementary Figure S7.png

Supplementary Figure S8

Click here to access/download
Supplementary Material
Supplementary Figure S8.png

Supplementary Figure S9

Click here to access/download
Supplementary Material
Supplementary Figure S9.png

Supplementary Notes

Click here to access/download

Supplementary Material

Additional InformationHaplogenome resolved assembly.docx

Supplementary Tables

Click here to access/download
Supplementary Material
Supplementary tables.xlsx

Supplementary Figure S2

Click here to access/download
Supplementary Material
Supplementary Figure S2.png
