# Supplementary figures and images for: Haplogenome assembly reveals structural variation in Eucalyptus interspecific hybrids
Source: Gigascience. 2023 Aug 26;12:giad064. doi: 10.1093/gigascience/giad064 (PMC10460159; doi:10.1093/gigascience/giad064)

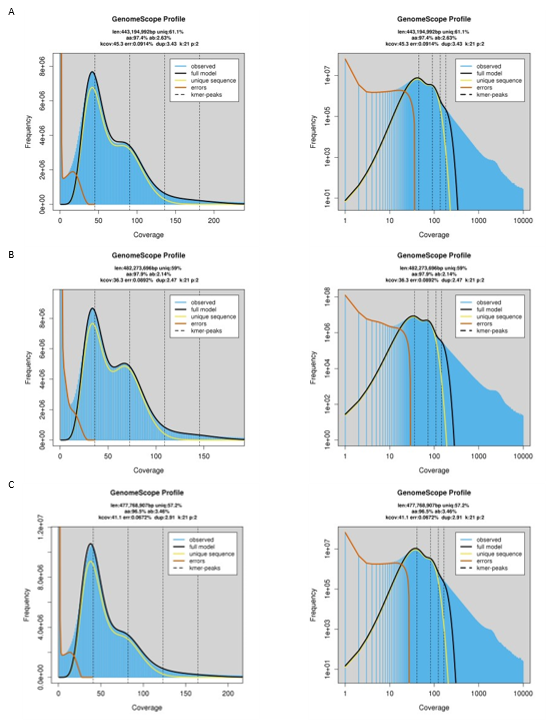

Supplement: giad064_Supplemental_Files [file giad064_supplemental_files.zip › Supplementary Figure S1.png]

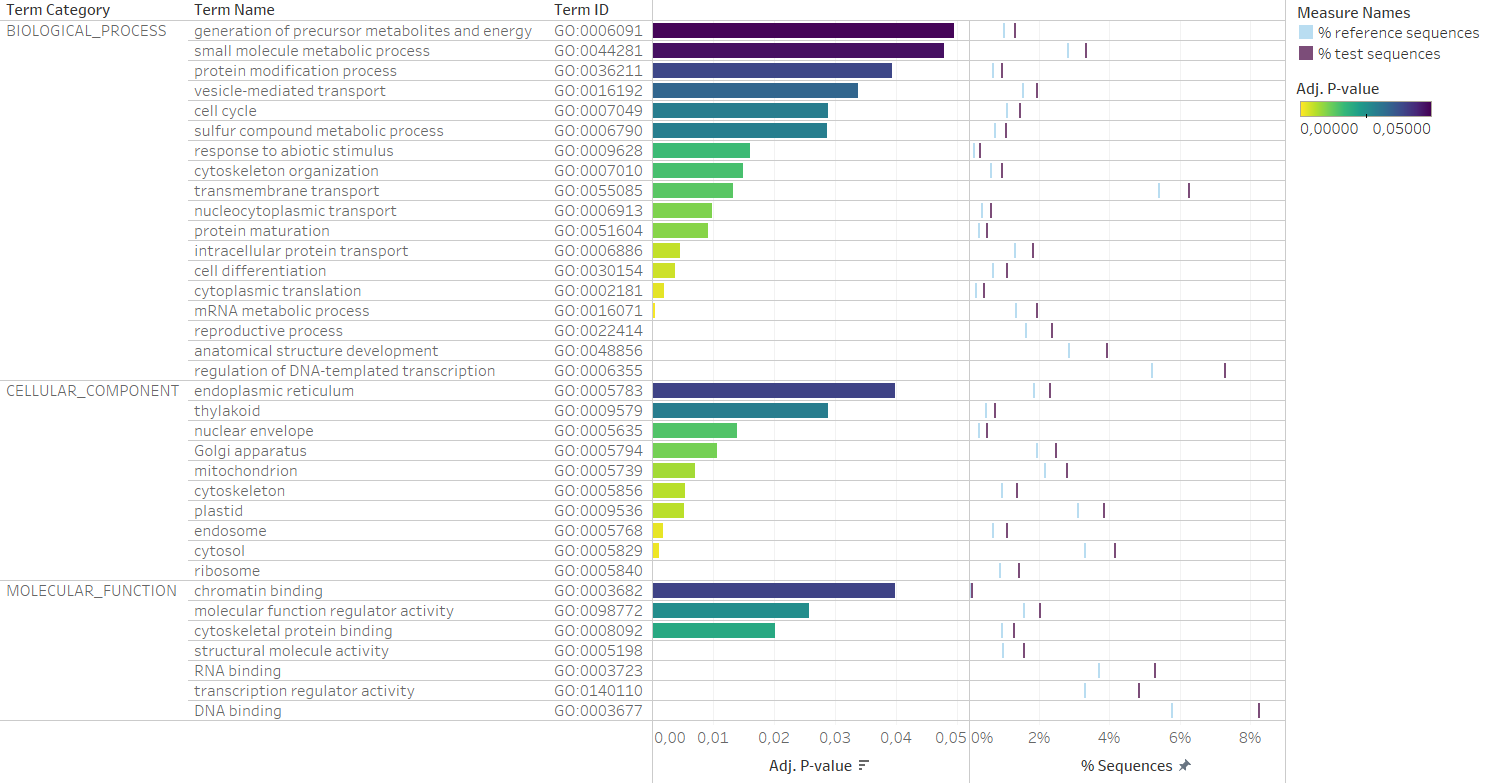

Supplement: giad064_Supplemental_Files [file giad064_supplemental_files.zip › Supplementary Figure S10.png]

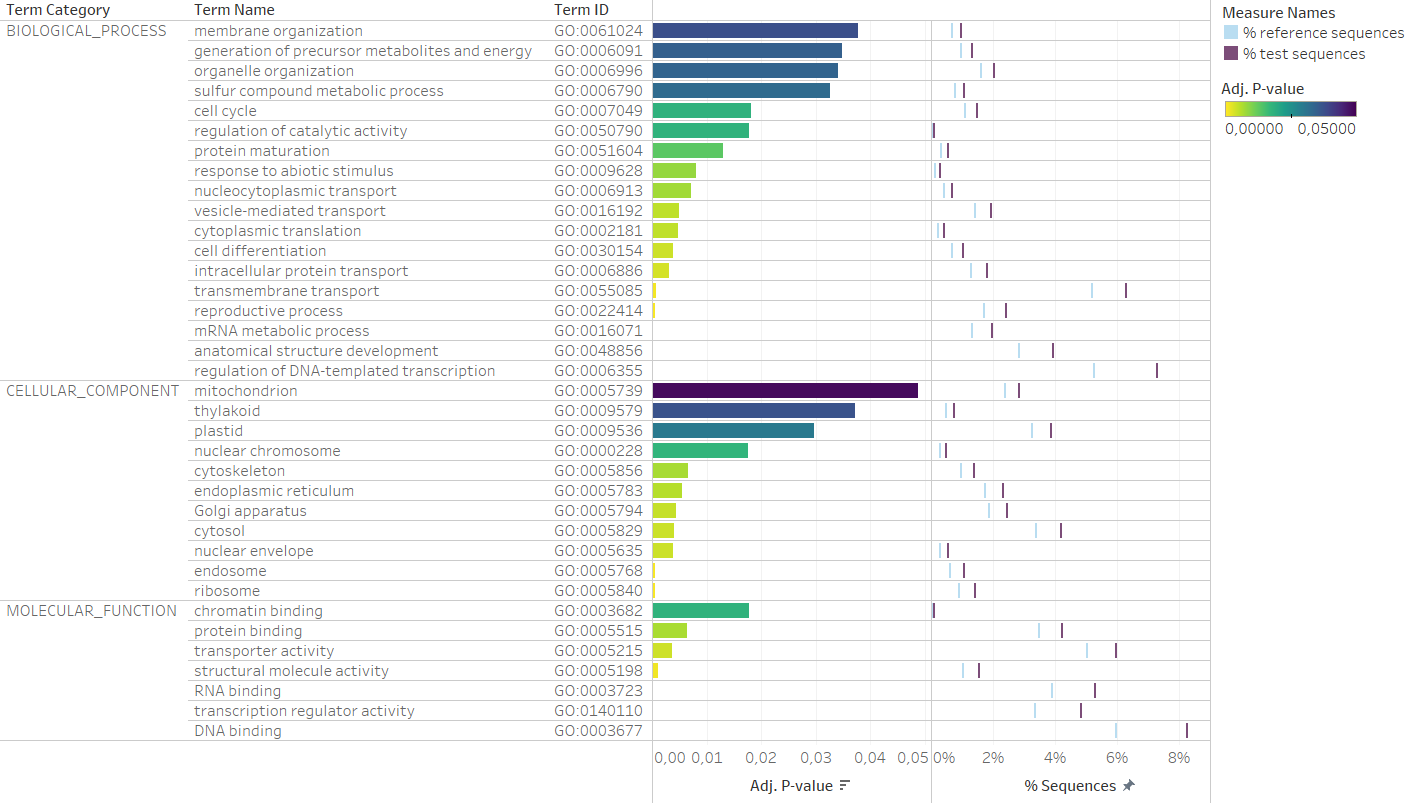

Supplement: giad064_Supplemental_Files [file giad064_supplemental_files.zip › Supplementary Figure S11.png]

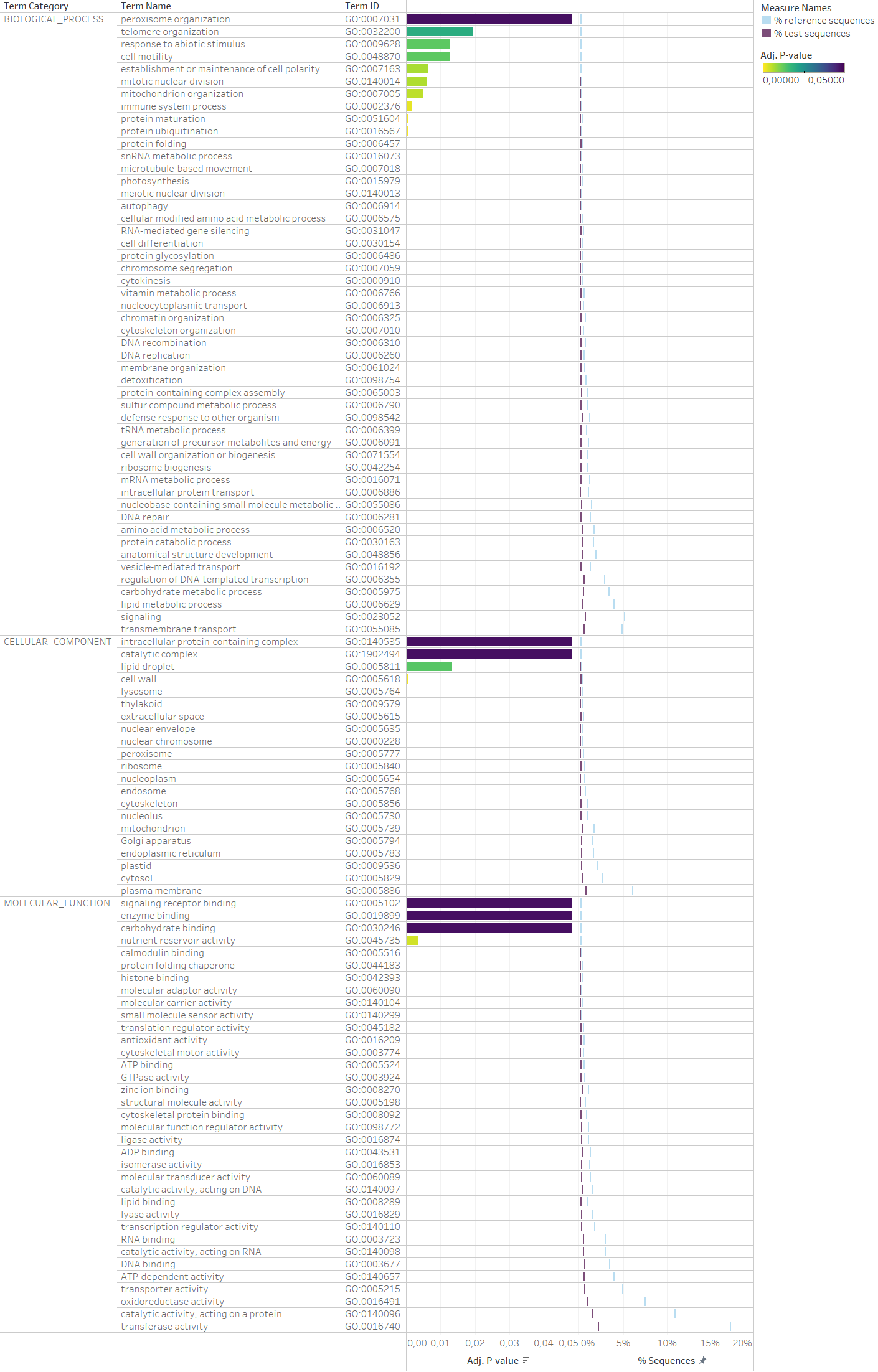

Supplement: giad064_Supplemental_Files [file giad064_supplemental_files.zip › Supplementary Figure S12.png]

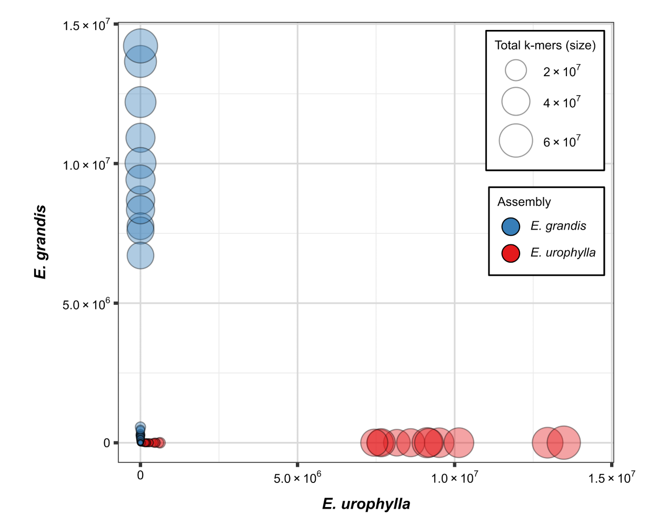

Supplement: giad064_Supplemental_Files [file giad064_supplemental_files.zip › Supplementary Figure S13.png]

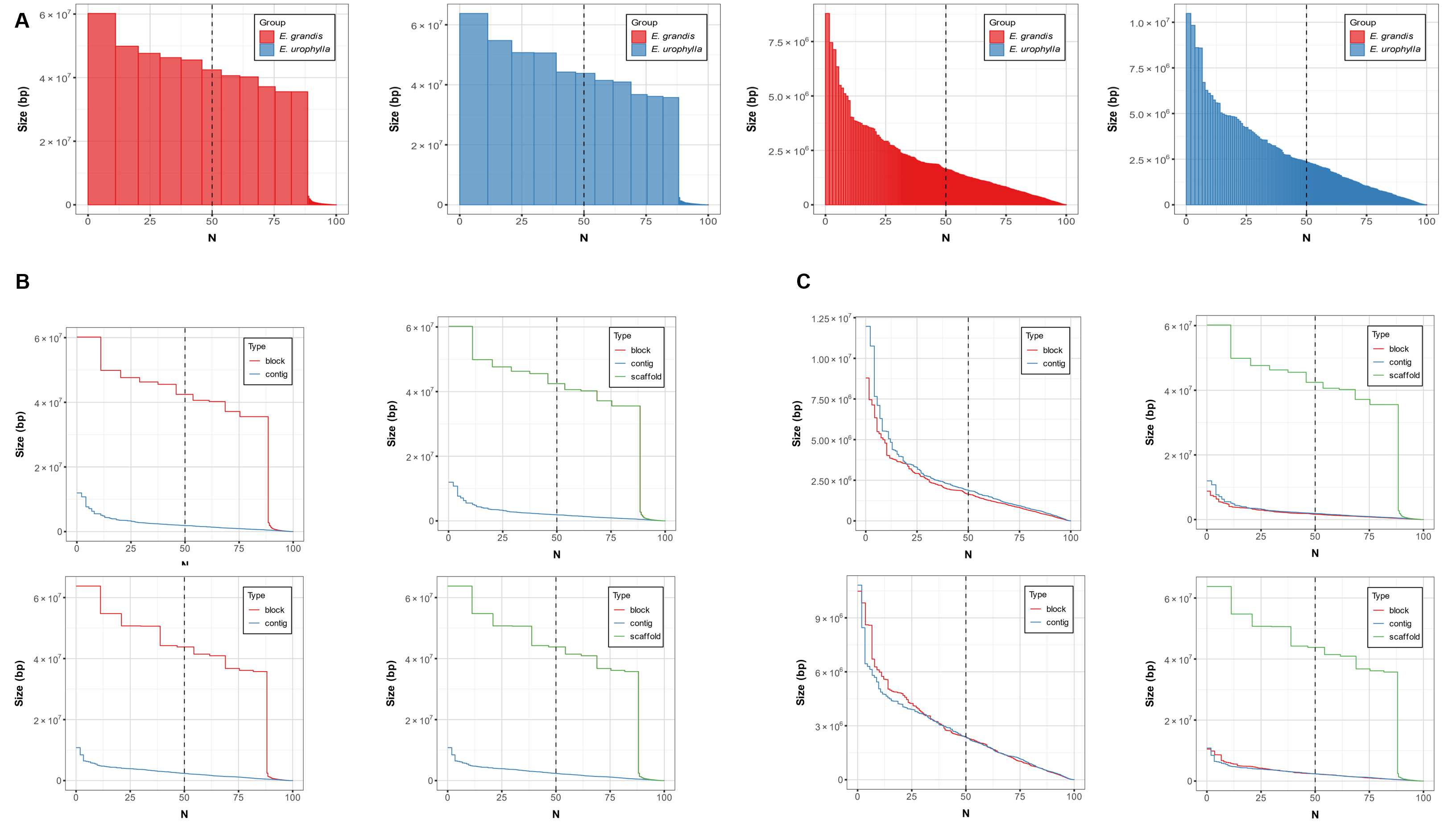

Supplement: giad064_Supplemental_Files [file giad064_supplemental_files.zip › Supplementary Figure S14.png]

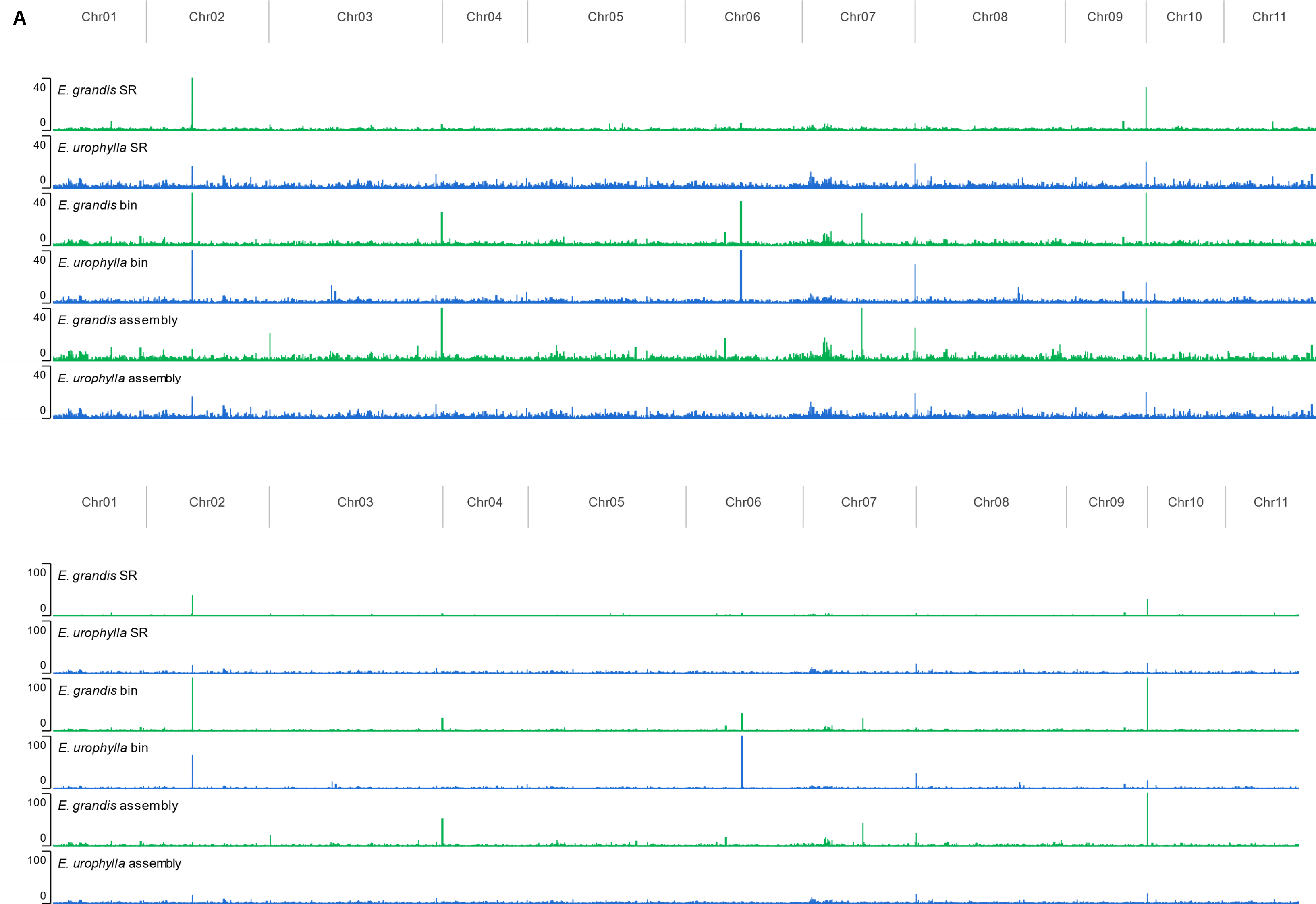

Supplement: giad064_Supplemental_Files [file giad064_supplemental_files.zip › Supplementary Figure S15A.png]

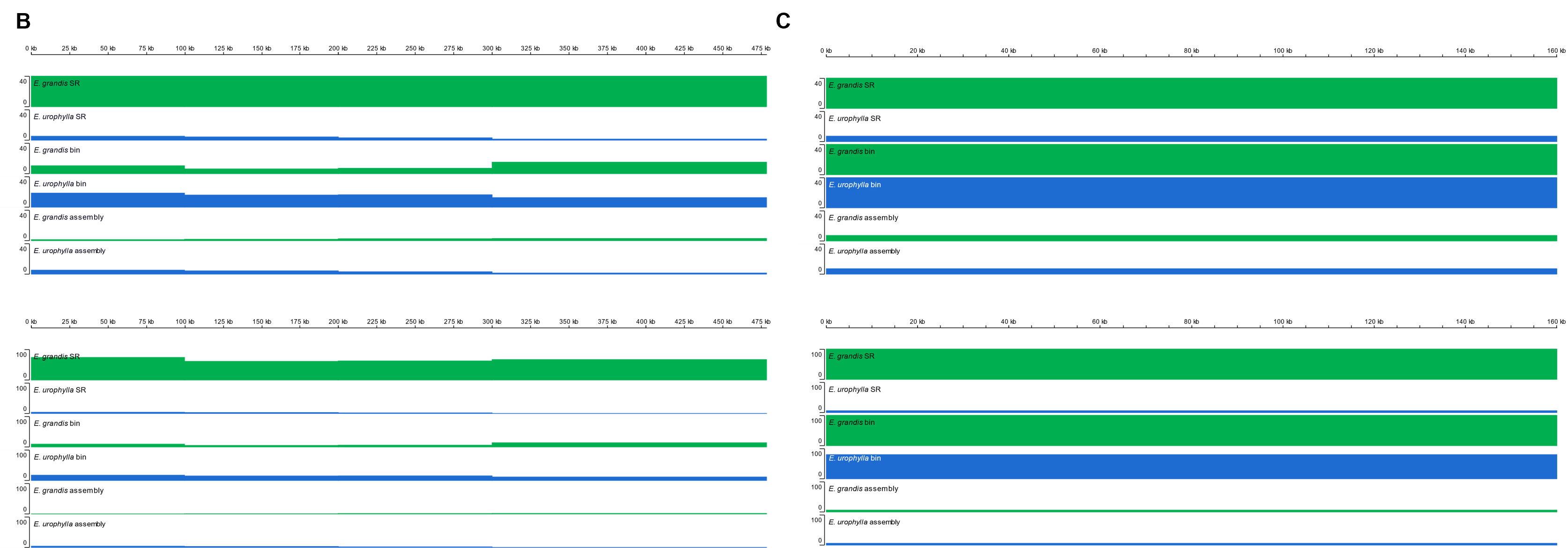

Supplement: giad064_Supplemental_Files [file giad064_supplemental_files.zip › Supplementary Figure S15B.png]

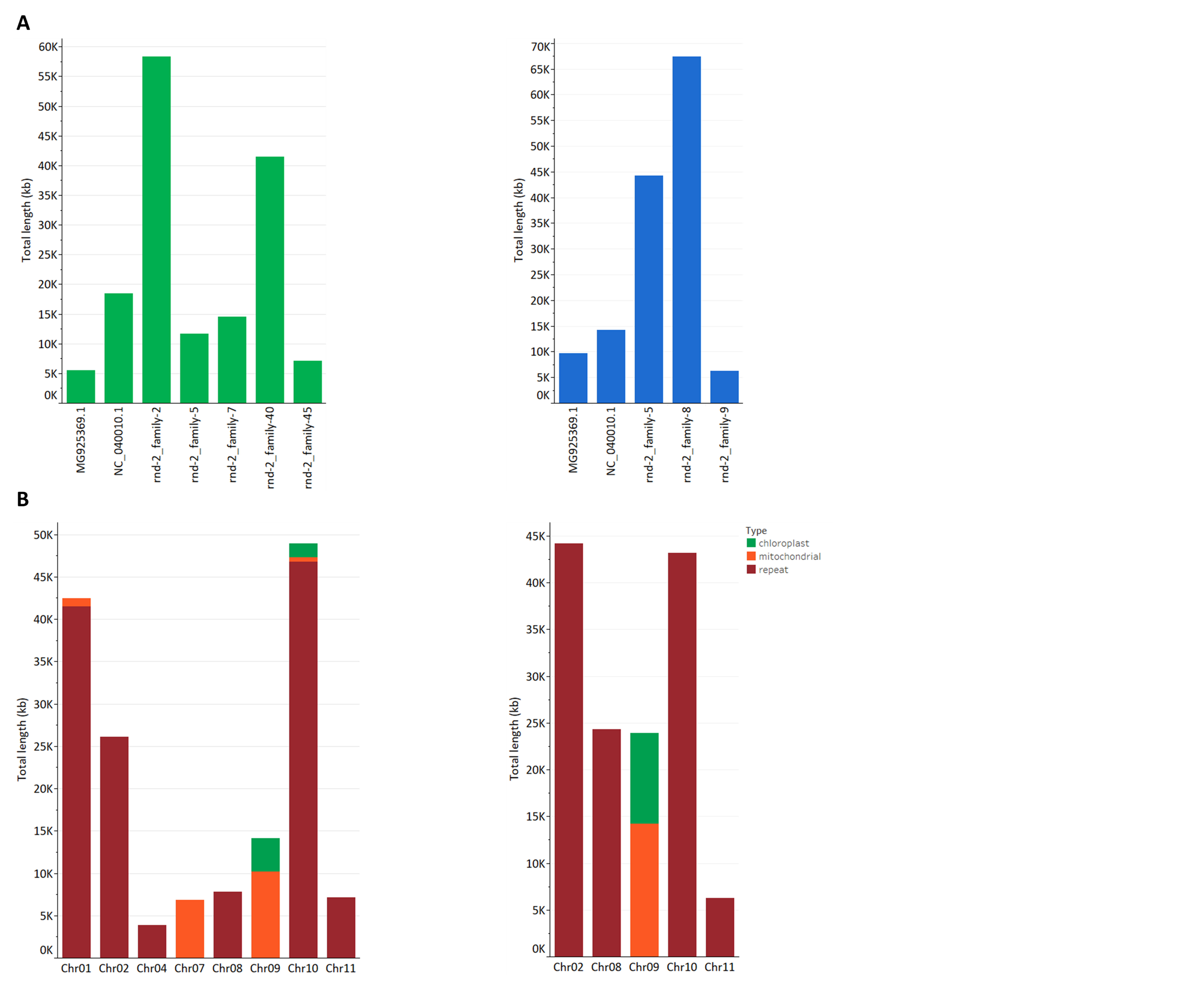

Supplement: giad064_Supplemental_Files [file giad064_supplemental_files.zip › Supplementary Figure S16.png]

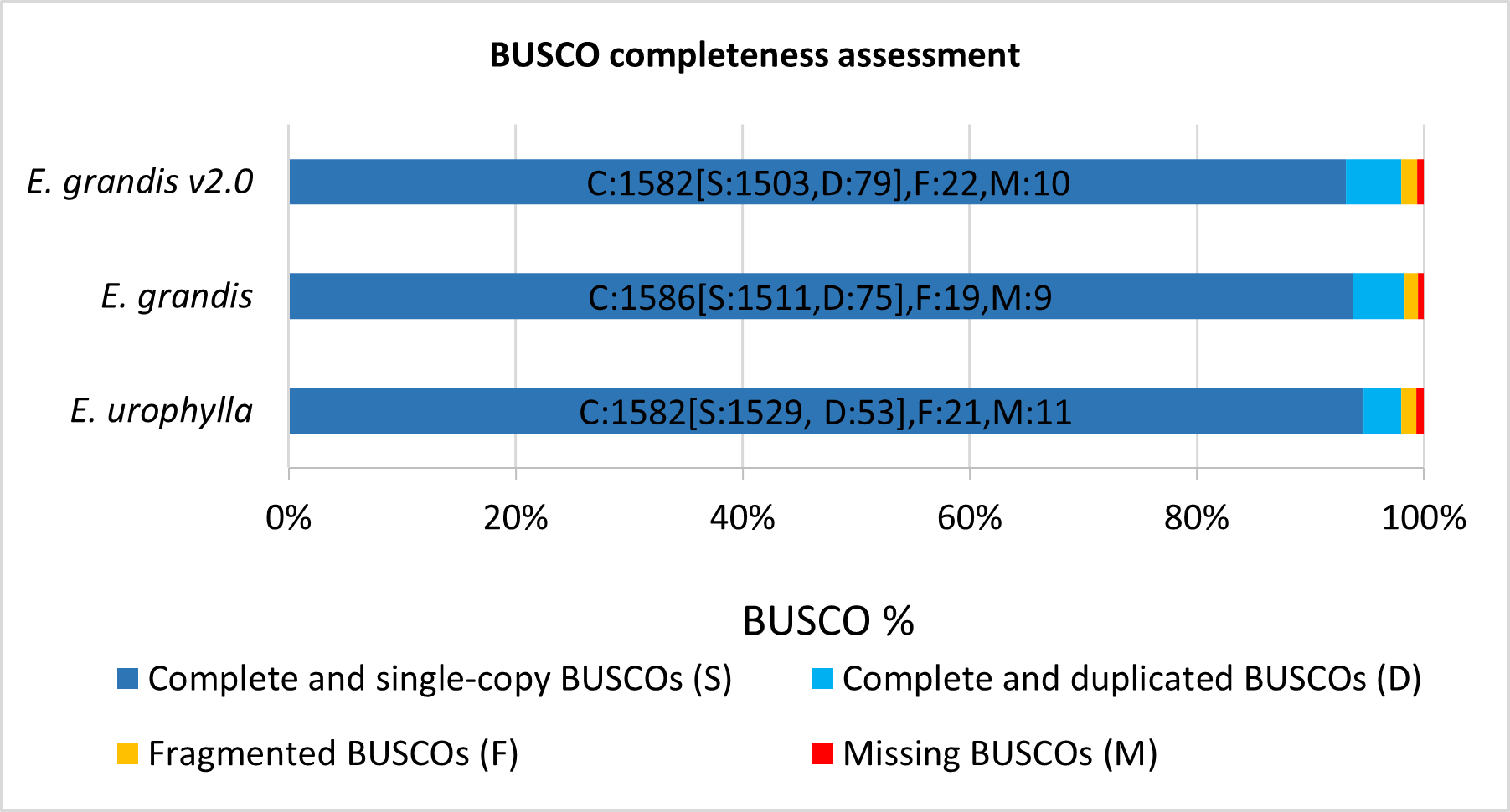

Supplement: giad064_Supplemental_Files [file giad064_supplemental_files.zip › Supplementary Figure S2.png]

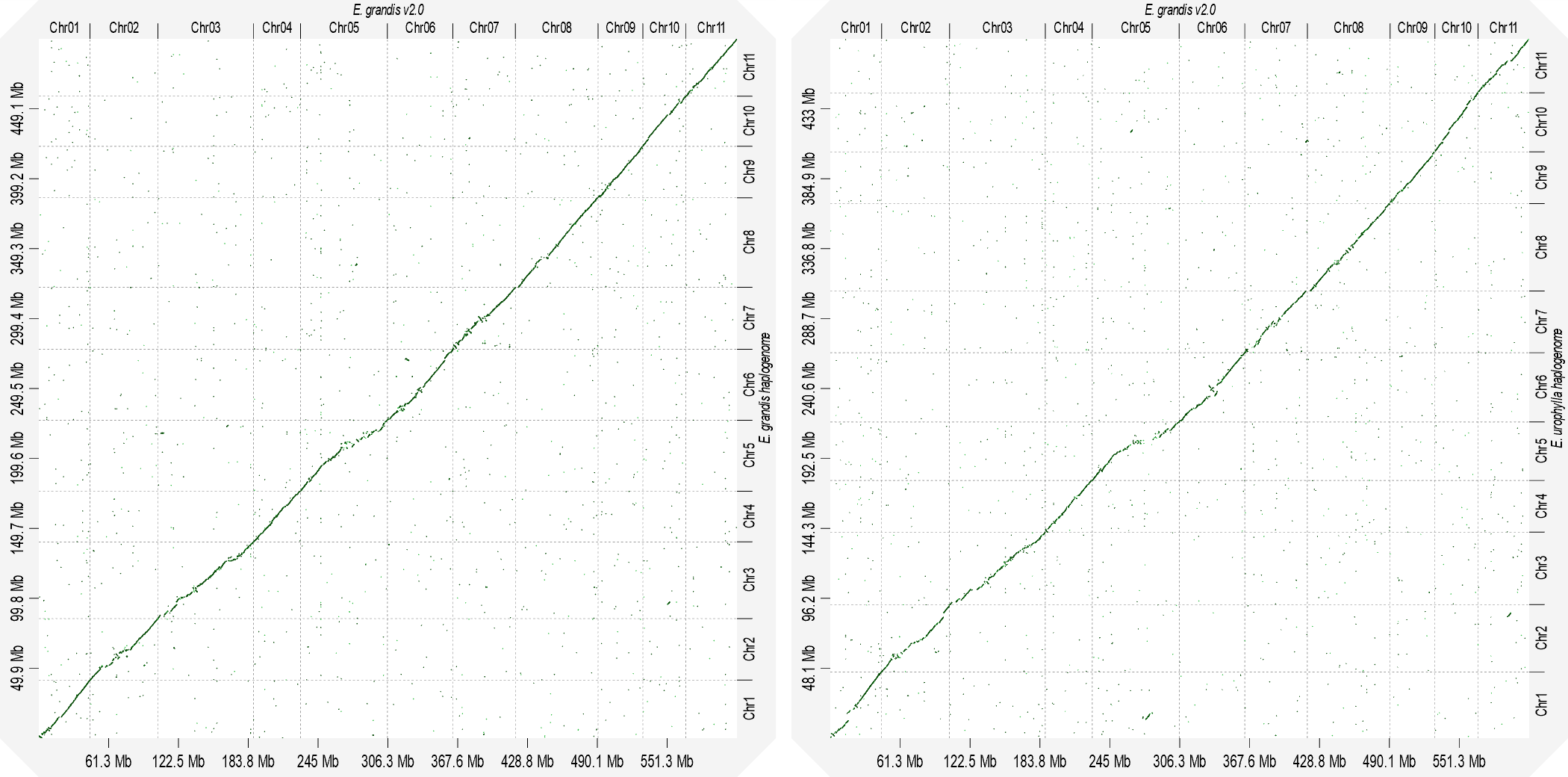

Supplement: giad064_Supplemental_Files [file giad064_supplemental_files.zip › Supplementary Figure S3.png]

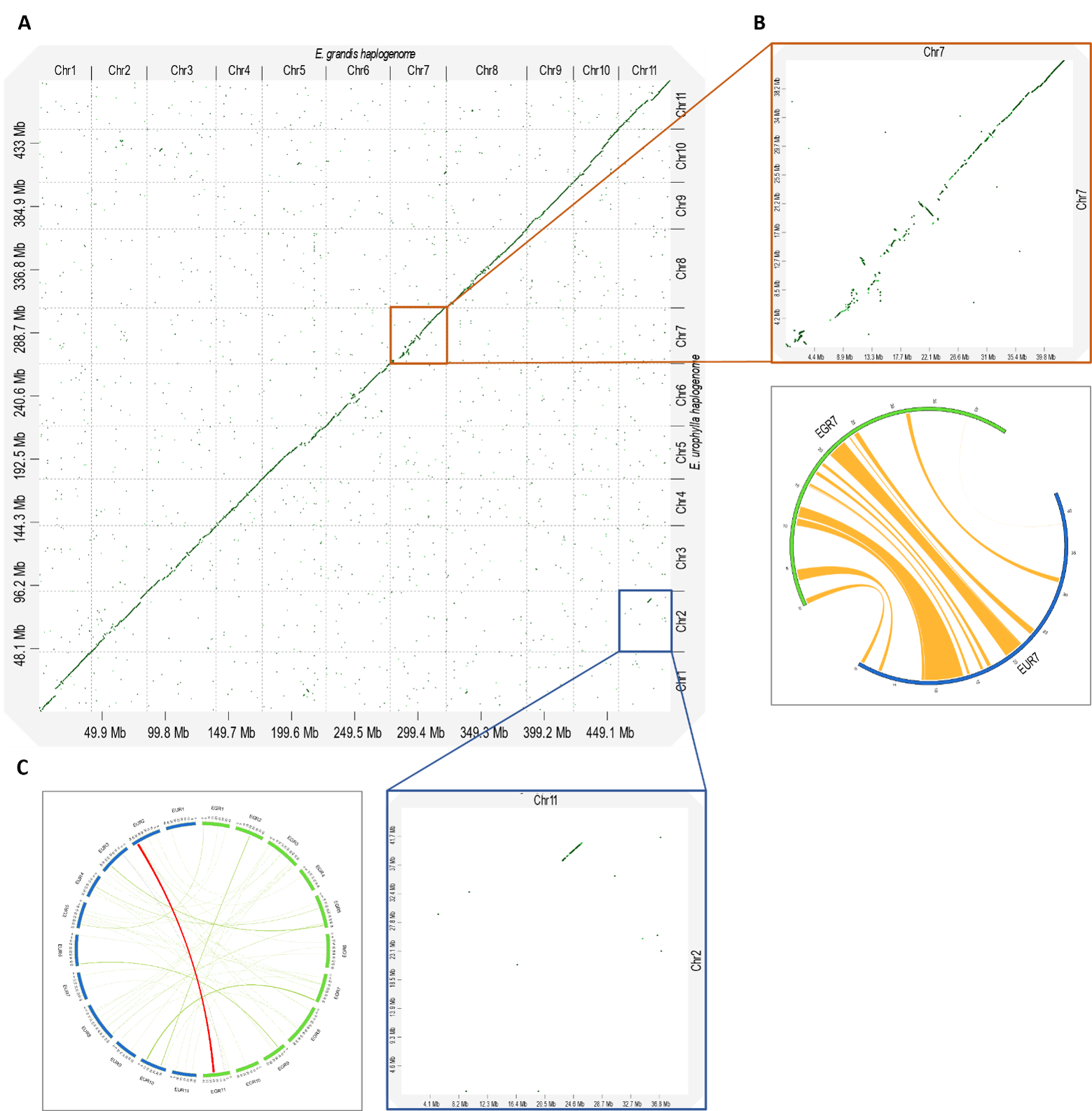

Supplement: giad064_Supplemental_Files [file giad064_supplemental_files.zip › Supplementary Figure S4.png]

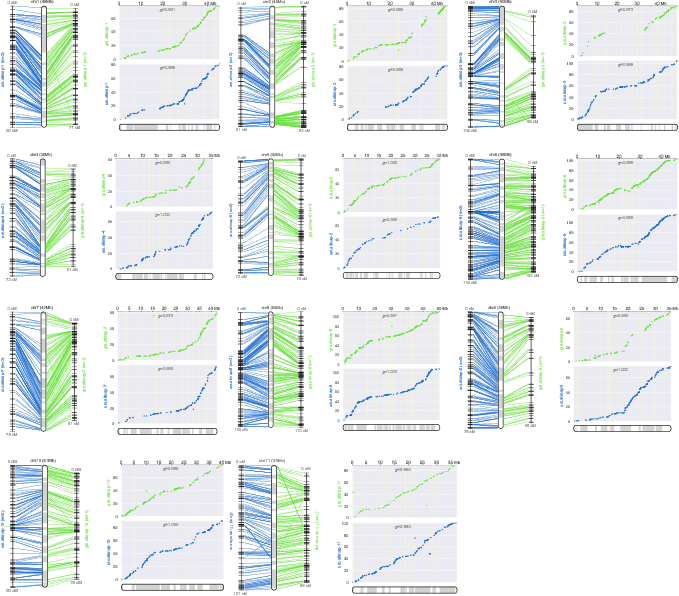

Supplement: giad064_Supplemental_Files [file giad064_supplemental_files.zip › Supplementary Figure S5.png]

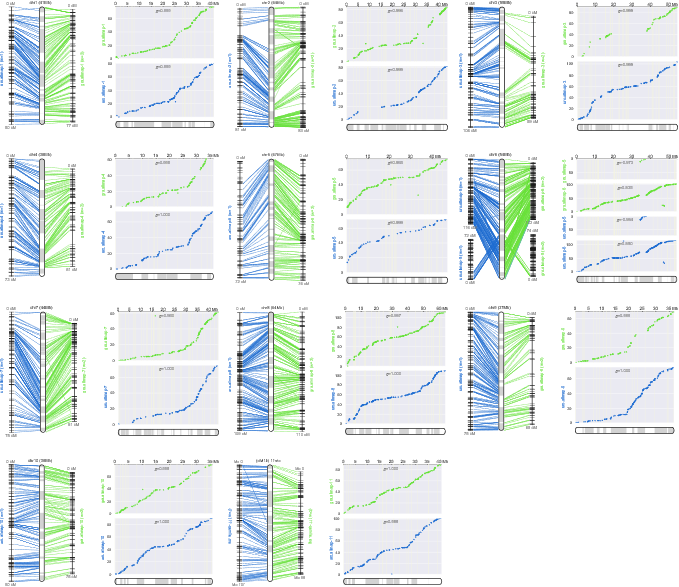

Supplement: giad064_Supplemental_Files [file giad064_supplemental_files.zip › Supplementary Figure S6.png]

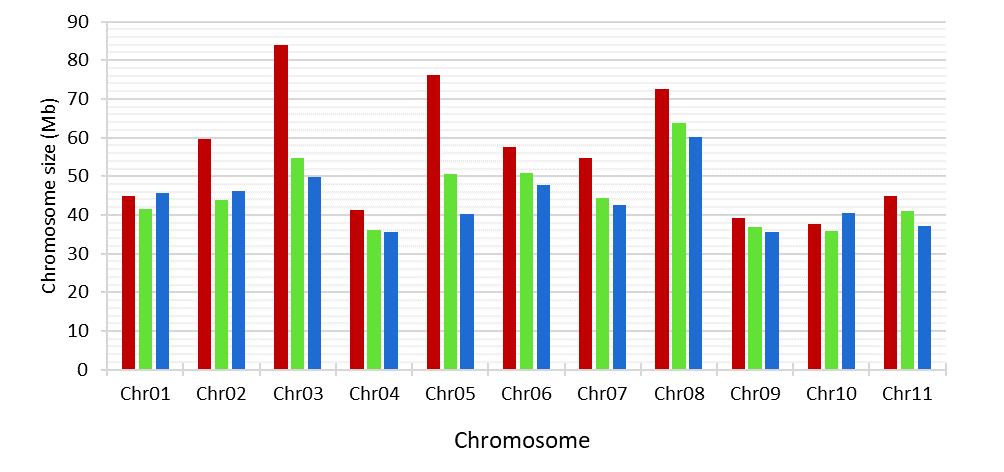

Supplement: giad064_Supplemental_Files [file giad064_supplemental_files.zip › Supplementary Figure S7.png]

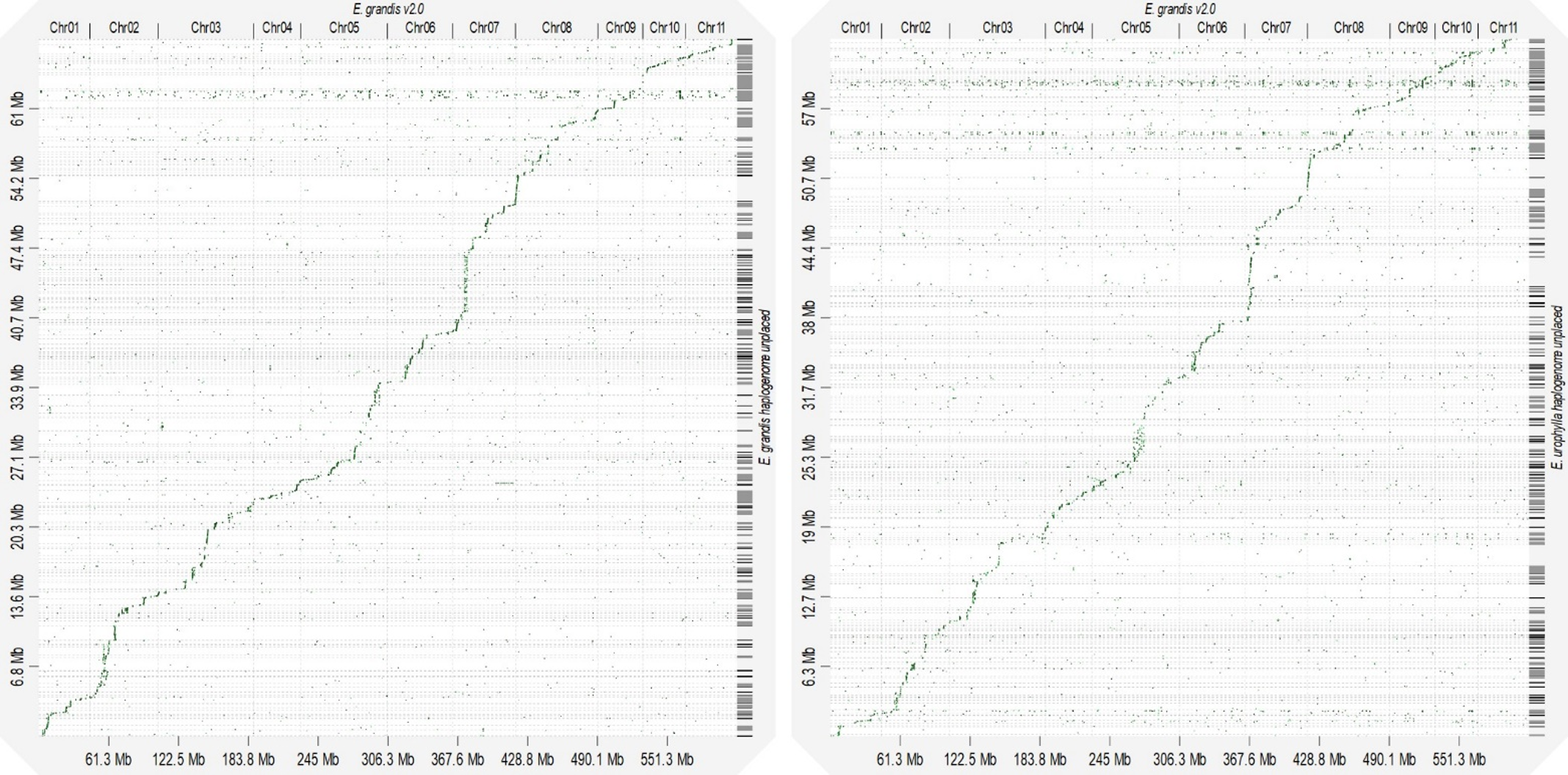

Supplement: giad064_Supplemental_Files [file giad064_supplemental_files.zip › Supplementary Figure S8.png]

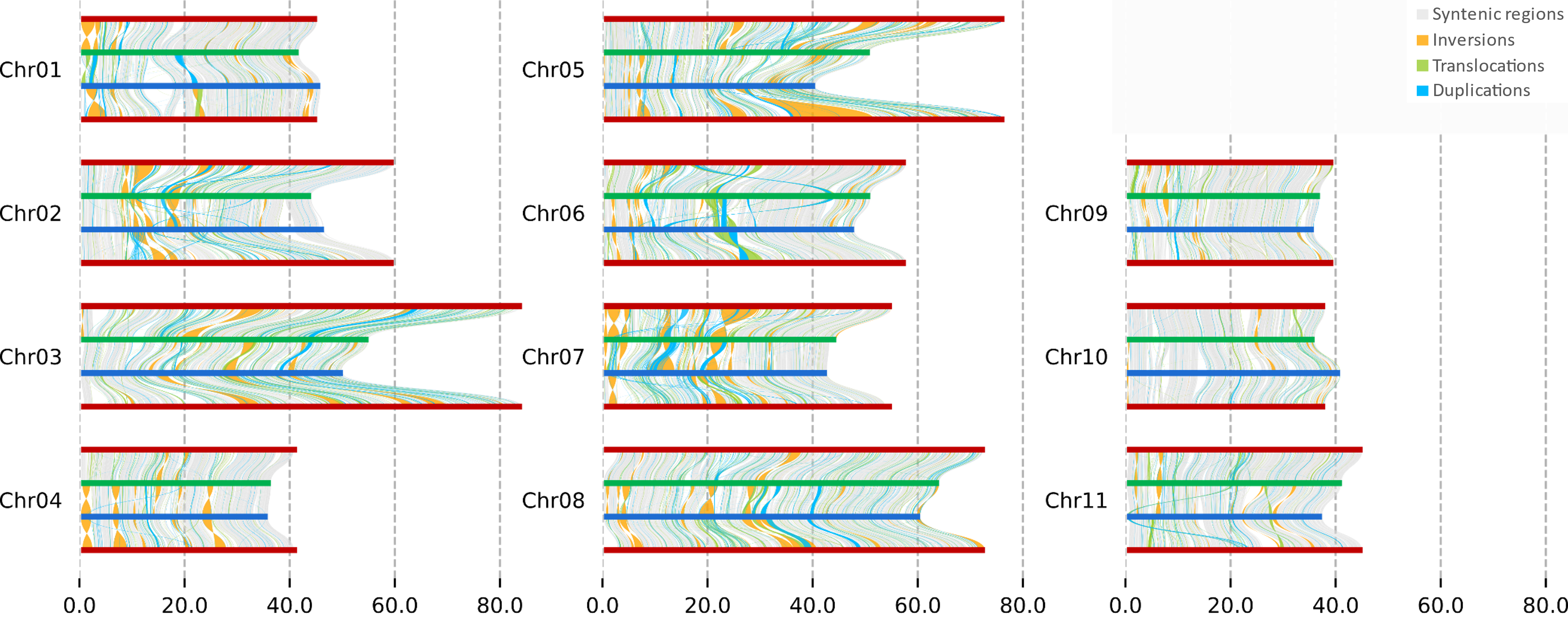

Supplement: giad064_Supplemental_Files [file giad064_supplemental_files.zip › Supplementary Figure S9.png]
