# Supplementary material for: Haplogenome assembly reveals structural variation in Eucalyptus interspecific hybrids
Source: Gigascience. 2023 Aug 26;12:giad064. doi: 10.1093/gigascience/giad064 (PMC10460159; doi:10.1093/gigascience/giad064)
Supplement: giad064_Response_to_Reviewer_Comments_Original_Submission [file giad064_response_to_reviewer_comments_original_submission.pdf]

Author's Response To Reviewer Comments

Close

Please find below the point-by-point format responses to reviewer comments answered within >>><<< sections . All results from additional analyses have been provided on the ftp link provided by GigaScience under the 2023_02_15 folder.

GIGA-D-22-00250
Haplogenome assembly reveals interspecific structural variation in Eucalyptus hybrids
Anneri Lotter; Julia Candotti; Tuan A. Duong; Eshchar Mizrachi; Jill L. Wegrzyn; Alexander Myburg
GigaScience

Dear Prof. Myburg,

Your manuscript "Haplogenome assembly reveals interspecific structural variation in Eucalyptus hybrids" (GIGA-D-22-00250) has been assessed by our reviewers. Although it is of interest, we are unable to consider it for publication in its current form. The reviewers have raised a number of points which we believe would improve the manuscript and may allow a revised version to be published in GigaScience.

Their reports, together with any other comments, are below. Please also take a moment to check our website at https://www.editorialmanager.com/giga/ for any additional comments that were saved as attachments.

In addition, please register any new software application in the bio.tools and SciCrunch.org databases to receive RRID (Research Resource Identification Initiative ID) and biotoolsID identifiers, and include these in your manuscript. Computational workflows should be registered in workflowhub.eu and the DOIs cited in the relevant places in the manuscript. These will facilitate tracking, reproducibility and re-use of your tool.

>>> Thank you for the feedback and opportunity to resubmit. We have followed your instructions and registered all of the software on SciCrunch.org to obtain RRID (Research Resource Identification Initiative ID) and we have included these in our manuscript. No new pipelines, workflows or software programs were developed in this manuscript and all of the used tools are publicly available, hence we did not register any of the software or workflows on biotoolsID or workflowhub.eu. We do refer in the text to the GitLab repository for all of these public programs and workflows. <<<

If you are able to fully address these points, we would encourage you to submit a revised manuscript to GigaScience. Once you have made the necessary corrections, please submit online at:

https://www.editorialmanager.com/giga/

If you have forgotten your username or password please use the "Send Login Details" link to get your login information. For security reasons, your password will be reset.

Please include a point-by-point within the 'Response to Reviewers' box in the submission system. Please ensure you describe additional experiments that were carried out and include a detailed rebuttal of any criticisms or requested revisions that you disagreed with. Please also ensure that your revised manuscript conforms to the journal style, which can be found in the Instructions for Authors on the journal homepage. If the data and code has been modified in the revision process please be sure to update the public versions of this too.

>>> Thank you. Please see the point-by-point rebuttal in the attached document below. We have ensured that the manuscript is formatted to the journal style. We have updated the public data were necessary. The

sequencing and assembly data are now available in NCBI and we provide the accession numbers in the manuscript. Additions and re-analyses are clearly noted in the text. <<<

The due date for submitting the revised version of your article is 15 Feb 2023.

I look forward to receiving your revised manuscript soon.

Best wishes,

Hongling Zhou
GigaScience
www.gigasciencejournal.com

Reviewer reports:
Reviewer #1:
This study, entitled "Haplogenome assembly reveals interspecific structural variation in Eucalyptus hybrids", has reported two haplotypes from Eucalyptus grandis and E. urophylla.
Both genomes are of high quality and high completeness. Nevertheless, why not directly and separately sequenced the Eucalyptus grandis and E. urophylla, and separately assembled each genome? In this way, the authors will not perform so much assembling steps to distinguish haplogenome.
>>> We thank the reviewer for the comment. However, the aim to assemble haplogenomes is the main point of the study. As we motivate in the manuscript, previous assemblies of highly outbred forest tree species have suffered from the inability to co-assemble highly divergent haplotypes, especially in intergenic regions. This has resulted in assemblies that are mosaics of the two haplogenomes in outbred forest trees. In conifers, this was overcome by sequencing DNA from the haploid megagametophyte. We propose here that sequencing of interspecific hybrids (with heterozygosity of over 3% in Eucalyptus) using a trio-binning approach, as pioneered in cattle by Koren et al. 2018, is actually a very productive approach in forest trees to rapidly recover high quality haplo-genomes. Furthermore, the F1 individuals are typically part of a genetic mapping family, which further facilitates scaffolding to chromosome scale. We therefore consider this a valuable approach to sequencing and assembly of highly outbred and complex tree genomes, which will facilitate genetic analysis of interspecific hybrids and heterosis in particular. It is indeed possible to these days achieve fully phased assemblies by combining long-read and Hi-C/Omni-C technologies, but our approach is a valid alternative in the context of hybrid breeding and having a linkage mapping population available. <<<

On the other hand, the authors have written a large paragraph to show the SV and SNP between both Eucalyptus species. However, the author only shown the number of SVs and SNPs, but did not show any relationship between the SV and biological characters. Could some SVs and SNPs involved in or impacted some genes can interpret some biological difference between Eucalyptus grandis and Eucalyptus grandis? In my view, only showing the number of SVs and SNPs is indeed fruitless for wide interests of this study. Some biological stories should be reported in a genome study.
>>> Thank you for this comment. We have investigated those genes that are in inverted or translocated segments of the genome. There are no significant biological enrichments. We address this in the last paragraph of the Results section “Structural Variant Analysis”. At this stage, we only have one haplogenome from each of the two species. We are in fact sequencing additional sets of trios and adding RNA-seq data for those trees in order to address the issue of impact of SVs and sequence variants (SNPs and Indels). That analysis benefits from having multiple haplogenomes samples from each of the parental species which will result in much more robust inference regarding interspecific vs intraspecific biological differences. Reporting the SVs and SNPs in this manuscript represents a valuable contribution to the community since it is the first detailed comparison of these two important genomes, especially within the context of an interspecific hybrid. Similarly, we can find large numbers of SNPs that are predicted to affect gene function (snpEff), but until more genomes are analysed of each species, we do not know whether those are fixed genetic differences between the two species, or segregating polymorphisms that happened to be present in the two parents of this trio. Eucalypts, being highly outbred and heterozygous, accumulate large amounts of genetic load which results in large numbers of genes with predicted functional mutations.

<<<

Please provide new figures with higher resolution. These figures are too much unclear.
>>> We have in fact provided the high resolution images separately as supplementary files. Some of them are very large and we have noticed that they generally lose resolution and quality during the rendering of the PDF. We will provide the images again in the revised version and work with the Editorial team to ensure that they are available for review. <<<

Please use the novel version of BUSCO V5.2.2, and indicate the used library.
>>> We have rerun the analysis with V5.2.2 and updated the information in the manuscript. We will also provide the raw result files from BUSCO via the FTP site (in the 2023_02_15 folder). <<<

What's the QUAST assessment result in this study?
>>> We included the QUAST assessment in Table 1 (contig N50, scaffold N50, number of contigs and scaffolds). <<<

The English language of this paper needs to be largely polished. Too much spelling and mistakes were appeared in the manuscript.
>>> Thank you. We have done a fine-combing of the text and corrected the remaining spelling mistakes and edited for style where necessary. <<<

Some minor suggestions:
The decimal places should be uniform, such as "(567 Mb and 545 Mb) to 97.9% BUSCO completion" and "scaffold N50 of 43.82 Mb and 42.45 Mb for the E. grandis and E. urophylla haplogenomes, respectively".
>>> We have updated the manuscript to have one decimal consistently (with a few exceptions where necessary).<<<

In 'All scripts used in this study is available on github.', 'is' should be 'are'.
>>> We have corrected this. <<<

The language of this sentence should be revised "Illumina short-reads were used for k-mer based genome size estimation was performed using Jellyfish v2.2.6 (Jellyfish, RRID:SCR_005491) [25] for 21-mers and visualised with GenomeScope v2.0"
>>> We have revised to say "K-mer based (21-mer) genome size estimation was performed using llumina short-reads as input for Jellyfish v2.2.6 (Jellyfish, RRID:SCR_005491) [25] and visualised with GenomeScope v2.0"<<<

For scaffolding step, why the authors removed all contigs smaller than 3kb?
>>> We removed there because they were much smaller than the average read length and likely represent single, truncated reads rather than contigs of long-reads. <<<

'The predicted gene space was' should be 'The predicted gene spaces were'.
>>> We have revised accordingly.<<<

For "a contig N50 of 3.91 Mb 1." and 'was greater than 88.0% 2', what're meaning of the last '1' and '2' in these sentences.
>>> These refer to Tables 1 and 2. The broken hyperlink has been fixed and updated in the revised

manuscript.<<<

In this sentence 'Approximately 3.3 μg of HMW DNA from was used without', 'from' what?
>>> This has been corrected in the revised manuscript (removed "from"). <<<

"a BUSCO completeness score of at least 95.3% was obtained for contigs anchored to one of the eleven chromosomes.", for one of the eleven chromosomes? Why contigs were only anchored to one chromosome?
>>> We note that the structure of the sentence may be confusing, the meaning implied is that the contigs that were anchored to any one of the 11 chromosomes have a 95.3% BUSCO completeness score. The sentence has been revised for clarity. <<<

Revise 'markers each.,'.
>>> This has been corrected in the revised manuscript.<<<

"BUSCO completeness scores of 94.6% and 95.8% was obtained", 'was' should be 'were'.
>>> This has been corrected in the revised manuscript.<<<

"Although there is a greater number of local variants compared to SVs", 'there is' should be 'there are'.
>>> This has been corrected in the revised manuscript.<<<

"respectively, Supplementary Table S3)" revised to 'respectively, (Supplementary Table S3)'.
>>> This has been corrected in the revised manuscript. <<<

'Mbp' revised to 'Mb'.
>>> This has been corrected in the revised manuscript.<<<

'assemblies was' should be 'assemblies were'.
>>> This has been corrected in the revised manuscript.<<<

Reviewer #2:
1. The CDS and protein sequences could not extracted from the file of masked.fasta with gff3 file when verifying the accuracy of genes loci and related proteins. The extract software is gffread in cufflinks 2.1.1. Please confirm the final assembly file that would upload to GigaDB.
>>> We have uploaded updated files on the FTP site (in the 2023_02_15 folder) and confirmed that the following command works:
gffread ./URO_coding_genes_CDS.gff3 -g ../../genome_assemblies/uro_final_masked.fasta -E -J -C -M -w gff3_uro_transcript_dup.fa -y gff3_uro_protein_dup.faa -d gff3_uro_dup.out
gffread ./GRA_coding_genes_CDS.gff3 -g ../../genome_assemblies/gra_final_masked.fasta -E -w gff3_gra_transcript_dup.fa -y gff3_gra_protein_dup.faa -d gff3_gra_dup.out
<<<

2. Confirmed the accuracy of gene predication, especially for ks calculation.

3. Before the repeat masked with the software of Repeatmasker, the final sequences were scanned with LTR_retriever and the LAI index have been generated in this folder. The LAI values were 20.55 and 18.06,

which could be classified the haplogenome assembly as the reference or gold level, please describe the LAI values after busco completeness in the revised manuscript.
>>> Thank you very much. We have included the LAI scores in the manuscript as suggested. <<<

4. The percentages of two largest subfamilies of LTR, Gypsy and Copia, were not presented in the supplementary TableS5.
>>> Supplementary table S5 has been updated to include the Gypsy and Copia LTR subfamilies. We have uploaded the revised supplementary file together with the revised manuscript. <<<

5. Two Eucalyptus genomes have been published (Nature 2014; Gigascience, 2020) and they were all not analysis the LTR insert time in detail. The insert times of all TE, Gypsy and Copia would highlighted this manuscript, especially the basic data have been presented with *.list in the LTR_harvest and LTR_retriever scan.
>>> We thank the reviewer for this comment. A detailed analysis of LTR insert times in Eucalyptus genomes will indeed be interesting. We are involved in a large-scale genome sequencing project with the US-Department of Energy - Joint Genome Institute (DOE-JGI) that will produce phased references for 10 additional eucalypt species, which would be a great opportunity to address this issue. Given that our present haplogenome manuscript is submitted for a Data Note, a detailed LTR insertion time analysis may be outside of the immediate scope. <<<

6. Did the special genes of each haplogenome classify? Which pathways or Go terms they enriched in?
>>> We are in the process of sequencing additional sets of trios for this species combination and adding RNA-seq data for those trees in order to address the issue of impact of SVs and sequence variants and to look at species specific-genes. That analysis benefits from having RNA sequencing data from the same F1 hybrid individuals for which genome assemblies are being produced. With multiple haplogenomes representing each of the two species, we will have more confidence to identify genes that are shared among individuals of the same species and absent in the other species. We did do a preliminary OrthoFinder analysis of the two haplogenomes in this manuscript and found that 833 (3260 genes) and 634 orthogroups (2332 genes) were uniquely detected in the E. grandis and E. urophylla haplogenome assemblies, respectively. Some of these may represent pan-genome variation within the two species and we will confirm authentic species-specific orthogroups and genes when we have multiple hapogenomes available for each species. <<<

7. Some SVs may be associated with the plant traits. The genes distributing in the regions of different SVs type should be furtherly identified and enriched with GO and KEGG.
>>> Thank you. This was also mentioned by Reviewer 1. We have briefly interrogated this in the "Structural variant analysis" section and refer the reviewer to Supplementary Figure 10. Given the diversity of SV types, large numbers of locations in the genome and large proportion of the genome included in SVs, we did not expect and indeed did not observe any informative significant genome-wide enrichments. A more detailed analysis of local enrichments around or within specific SVs will yield more insight into local gene enrichments possibly contributing to trait variation. <<<

8. "Syntenic gene pairs between the E. grandis and E. urophylla haplogenomes were identified using a python version of MCScan, JCVI v1.1.18. "Syntenic gene pairs in Figure 4 seemed only from JCVI, not using MCScan.
>>> Indeed, throughput the manuscript we use the output from the python version of MCScan, a module implemented within the JCVI utility libraries software package, not MCScanX. <<<

9. The reference cite should be consistent, such as Candotti et al in the section of Genome scaffolding should be revised.
>>> This has been addressed in the revised manuscript. <<<

10. Language should be improved and modified by academic editor.
>>> We have edited the manuscript for language, style and spelling. <<<

Close
