# Supplementary material for: Haplogenome assembly reveals structural variation in Eucalyptus interspecific hybrids
Source: Gigascience. 2023 Aug 26;12:giad064. doi: 10.1093/gigascience/giad064 (PMC10460159; doi:10.1093/gigascience/giad064)
Supplement: giad064_Response_to_Reviewer_Comments_Revision_1 [file giad064_response_to_reviewer_comments_revision_1.pdf]

Author's Response To Reviewer Comments

Close

1. The specific genes of each haplogenome have been preliminary analysis by OrthoFinder, it is best to present the KEGG and Go term enrichments of specific genes in two genome and described the results in the section of Genome annotation, the enrichment results were recommended to present as supplementary tables. This analysis would not compete interests with the following research about pan-genome analysis. If the authors recked this results were re-used in the prospective studies in the future, the genes without collinearity relationship between two haplogenomes with MCScanX analysis could be also considered as the specific genes.

>>> We have updated the GO enrichment analyses to include genes that did not show collinearity in the MCScan gene synteny analyses. We have also included KEGG pathway enrichment analyses for the genes contained within inverted and translocated gene blocks, as well as genes that did not show collinearity in the MCScan gene synteny analyses. The manuscript has been updated accordingly. Congruent with the close relationship of the two species, we do not see strong KEGG pathway enrichments in the genes that are non-collinear between the two genomes. Of course, some of those genes may individually contribute to interspecific differentiation, e.g. genes that have gained different expression regulation as a result of the structural variation between the two genomes. Such individual gene differences would not result in GO enrichments at the genome-wide level. <<<

2. The gff3 files uploaded in the 2023_02_15 folder are still not the typical gff3 format by lacking exon information. Although the sequences of coding sequences and proteins could be extracted from *.fasta files with the fixed gff3 files, 115 and 97 coding sequences contained the premature stop codons resulting these CDS could not be translated into protein sequences accurately in GRA or URO genome, respectively. The protein and CDS sequences were extracted with the comment:

```
gffread URO.fixed.gff3 -g uro_final_masked.fasta -x uro.cds
gffread URO.fixed.gff3 -g uro_final_masked.fasta -y uro.pep
```

These inaccurate genes should be corrected before upload the dataset to GigaDB. The ID of these genes were list in the following:

>>> To address this issue, we have removed all genes shorther than 30 amino acids resulting in 12 GRA and 9 URO genes being completely excluded from the final annotation. The remaining genes were truncated at the premature stop and the protein sequences were realigned to the genome using miniprot to update the gff3 file. The updated gff3 file is provided via the ftp drive. The original sequences are also provided (during the previous submission) and the manuscript updated with a list of truncated proteins. Some of the these are potentially authentic nonsense mutations in the two parental genomes and should be further investigated in the future starting with gene sequencing of other members of the two species to determine whether these are fixed genetic differences. <<<

Close
