# Supplementary material for: Haplogenome assembly reveals structural variation in Eucalyptus interspecific hybrids
Source: Gigascience. 2023 Aug 26;12:giad064. doi: 10.1093/gigascience/giad064 (PMC10460159; doi:10.1093/gigascience/giad064)
Supplement: giad064_Reviewer_1_Report_Original_Submission — Chao Bian -- 11/10/2022 Reviewed [file giad064_reviewer_1_report_original_submission.pdf]

Reviewer Report

Title: Haplogenome assembly reveals structural variation in interspecific Eucalyptus hybrids

Version: Original Submission **Date:** 11/10/2022

Reviewer name: Chao Bian

Reviewer Comments to Author:

This study, entitled "Haplogenome assembly reveals interspecific structural variation in Eucalyptus hybrids", has reported two haplotypes from Eucalyptus grandis and E. urophylla.Both genomes are of high quality and high completeness. Nevertheless, why not directly and separately sequenced the Eucalyptus grandis and E. urophylla, and separately assembled each genome? In this way, the authors will not perform so much assembling steps to distinguish haplogenome.On the other hand, the authors have written a large paragraph to show the SV and SNP between both Eucalyptus species. However, the author only shown the number of SVs and SNPs, but did not show any relationship between the SV and biological characters. Could some SVs and SNPs involved in or impacted some genes can interpret some biological difference between Eucalyptus grandis and Eucalyptus grandis?In my view, only showing the number of SVs and SNPs is indeed fruitless for wide interests of this study. Some biological stories should be reported in a genome study.Please provide new figures with higher resolution. These figures are too much unclear.Please use the novel version of BUSCO V5.2.2, and indicate the used library.What's the QUAST assessment result in this study?The English language of this paper needs to be largely polished. Too much spelling and mistakes were appeared in the manuscript.Some minor suggestions:The decimal places should be uniform, such as "(567 Mb and 545 Mb) to 97.9% BUSCO completion" and "scaffold N50 of 43.82 Mb and 42.45 Mb for the E. grandis and E. urophylla haplogenomes, respectively".In 'All scripts used in this study is available on github.', 'is' should be 'are'.The language of this sentence should be revised "Illumina short-reads were used for k-mer based genome size estimation was performed using Jellyfish v2.2.6 (Jellyfish, RRID:SCR_005491) [25] for 21-mers and visualised with GenomeScope v2.0"For scaffolding step, why the authors removed all contigs smaller than 3kb?'The predicted gene space was' should be 'The predicted gene spaces were'.For "a contig N50 of 3.91 Mb 1." and 'was greater than 88.0% 2', what're meaning of the last '1' and '2' in these sentences.In this sentence 'Approximately 3.3 Î¼g of HMW DNA from was used without', 'from' what?"a BUSCO completeness score of at least 95.3% was obtained for contigs anchored to one of the eleven chromosomes.", for one of the eleven chromosomes? Why contigs were only anchored to one chromosome?Revise 'markers each.,'."BUSCO completeness scores of 94.6% and 95.8% was obtained", 'was' should be 'were'."Although there is a greater number of local variants compared to SVs", 'there is' should be 'there are'."respectively, Supplementary Table S3)" revised to 'respectively, (Supplementary Table S3)'.'Mbp' revised to 'Mb'.'assemblies was' should be 'assemblies were'.

Level of Interest

Please indicate how interesting you found the manuscript: Choose an item.

Quality of Written English

Please indicate the quality of language in the manuscript: Choose an item.

Declaration of Competing Interests

Please complete a declaration of competing interests, considering the following questions:

- Have you in the past five years received reimbursements, fees, funding, or salary from an organisation that may in any way gain or lose financially from the publication of this manuscript, either now or in the future?
- Do you hold any stocks or shares in an organisation that may in any way gain or lose financially from the publication of this manuscript, either now or in the future?
- Do you hold or are you currently applying for any patents relating to the content of the manuscript?
- Have you received reimbursements, fees, funding, or salary from an organization that holds or has applied for patents relating to the content of the manuscript?
- Do you have any other financial competing interests?
- Do you have any non-financial competing interests in relation to this paper?

If you can answer no to all of the above, write 'I declare that I have no competing interests' below. If your reply is yes to any, please give details below.

I declare that I have no competing interests

I agree to the open peer review policy of the journal. I understand that my name will be included on my report to the authors and, if the manuscript is accepted for publication, my named report including any attachments I upload will be posted on the website along with the authors' responses. I agree for my report to be made available under an Open Access Creative Commons CC-BY license (http://creativecommons.org/licenses/by/4.0/). I understand that any comments which I do not wish to be included in my named report can be included as confidential comments to the editors, which will not be published.

Choose an item.

To further support our reviewers, we have joined with Publons, where you can gain additional credit to further highlight your hard work (see: https://publons.com/journal/530/gigascience). On publication of this paper, your review will be automatically added to Publons, you can then choose whether or not to claim your Publons credit. I understand this statement.

Yes Choose an item.
