# Supplementary material for: Haplogenome assembly reveals structural variation in Eucalyptus interspecific hybrids
Source: Gigascience. 2023 Aug 26;12:giad064. doi: 10.1093/gigascience/giad064 (PMC10460159; doi:10.1093/gigascience/giad064)
Supplement: giad064_Reviewer_2_Report_Original_Submission — Xupo Ding -- 11/16/2022 Reviewed [file giad064_reviewer_2_report_original_submission.pdf]

Reviewer Report

Title: Haplogenome assembly reveals structural variation in interspecific Eucalyptus hybrids

Version: Original Submission **Date:** 11/16/2022

Reviewer name: Xupo Ding

Reviewer Comments to Author:

1. The CDS and protein sequences could not extracted from the file of masked.fasta with gff3 file when verifying the accuracy of genes loci and related proteins. The extract software is gffread in cufflinks 2.1.1. Please confirm the final assembly file that would upload to GigaDB.2. Confirmed the accuracy of gene predication, especially for ks calculation.3. Before the repeat masked with the software of Repeatmasker, the final sequences were scanned with LTR_retriever and the LAI index have been generated in this folder. The LAI values were 20.55 and 18.06, which could be classified the haplogenome assembly as the reference or gold level, please describe the LAI values after busco completeness in the revised manuscript.4. The percentages of two largest subfamilies of LTR, Gypsy and Copia, were not presented in the supplementary TableS5.5. Two Eucalyptus genomes have been published (Nature 2014; Gigascience, 2020) and they were all not analysis the LTR insert time in detail. The insert times of all TE, Gypsy and Copia would highlighted this manuscript, especially the basic data have been presented with *.list in the LTR_harvest and LTR_retriever scan.6. Did the special genes of each haplogenome classify? Which pathways or Go terms they enriched in?7. Some SVs may be associated with the plant traits. The genes distributing in the regions of different SVs type should be furtherly identified and enriched with GO and KEGG.8. "Syntenic gene pairs between the E. grandis and E. urophylla haplogenomes were identified using a python version of MCScan, JCVI v1.1.18."Syntenic gene pairs in Figure 4 seemed only from JCVIï¼Œnot using MCScan.9. The reference cite should be consistent, such as Candotti et al in the section of Genome scaffolding should be revised.10. Language should be improved and modified by academic editor.

Level of Interest

Please indicate how interesting you found the manuscript: Choose an item.

Quality of Written English

Please indicate the quality of language in the manuscript: Choose an item.

Declaration of Competing Interests

Please complete a declaration of competing interests, considering the following questions:

- Have you in the past five years received reimbursements, fees, funding, or salary from an organisation that may in any way gain or lose financially from the publication of this manuscript, either now or in the future?
- Do you hold any stocks or shares in an organisation that may in any way gain or lose financially from the publication of this manuscript, either now or in the future?
- Do you hold or are you currently applying for any patents relating to the content of the manuscript?
- Have you received reimbursements, fees, funding, or salary from an organization that holds or has applied for patents relating to the content of the manuscript?
- Do you have any other financial competing interests?
- Do you have any non-financial competing interests in relation to this paper?

If you can answer no to all of the above, write 'I declare that I have no competing interests' below. If your reply is yes to any, please give details below.

I declare that I have no competing interests

I agree to the open peer review policy of the journal. I understand that my name will be included on my report to the authors and, if the manuscript is accepted for publication, my named report including any attachments I upload will be posted on the website along with the authors' responses. I agree for my report to be made available under an Open Access Creative Commons CC-BY license (http://creativecommons.org/licenses/by/4.0/). I understand that any comments which I do not wish to be included in my named report can be included as confidential comments to the editors, which will not be published.

Choose an item.

To further support our reviewers, we have joined with Publons, where you can gain additional credit to further highlight your hard work (see: https://publons.com/journal/530/gigascience). On publication of this paper, your review will be automatically added to Publons, you can then choose whether or not to claim your Publons credit. I understand this statement.

Yes Choose an item.
